# Upstream open reading frames dynamically modulate CLOCK protein translation to regulate circadian rhythms and sleep

**DOI:** 10.1371/journal.pbio.3003173

**Published:** 2025-05-12

**Authors:** Yuanqiang Sun, Ke Shui, Qinyu Li, Chenlu Liu, Wanting Jin, Jian-Quan Ni, Jian Lu, Luoying Zhang

**Affiliations:** 1 State Key Laboratory of Gene Function and Modulation Research, Center for Bioinformatics, School of Life Sciences, Peking University, Beijing, China; 2 College of Biomedicine and Health, College of Life Science and Technology, Huazhong Agricultural University, Wuhan, China; 3 Key Laboratory of Molecular Biophysics of Ministry of Education, College of Life Science and Technology, Huazhong University of Science and Technology, Wuhan, China; 4 Gene Regulatory Lab, School of Medicine, Tsinghua University, Beijing, China; 5 Beijing Advanced Center of RNA Biology (BEACON), Peking University, Beijing, China; 6 Hubei Province Key Laboratory of Oral and Maxillofacial Development and Regeneration, Wuhan, China; Fundacion Instituto Leloir, ARGENTINA

## Abstract

The circadian rhythm is an evolutionarily conserved mechanism with translational regulation increasingly recognized as pivotal in its modulation. In this study, we found that upstream open reading frames (uORFs) are enriched in *Drosophila* circadian rhythm genes, with particularly conserved uORFs present in core circadian clock genes. We demonstrate evidence that the uORFs of the core clock gene, *Clock* (*Clk*), rhythmically and substantially attenuate CLK protein translation in *Drosophila*, with pronounced suppression occurring during daylight hours. Eliminating *Clk* uORFs leads to increased CLK protein levels during the day and results in a shortened circadian cycle, along with a broad shift in clock gene expression rhythms. Notably, *Clk* uORF deletion also augments morning sleep by reducing dopaminergic activity. Beyond daily circadian adjustments, *Clk* uORFs play a role in modulating sleep patterns in response to seasonal daylight variations. Furthermore, the *Clk* uORFs act as an important regulator to shape the rhythmic expression of a vast array of genes and influence multifaceted physiological outcomes. Collectively, our research sheds light on the intricate ways uORFs dynamically adjust downstream coding sequences to acclimate to environmental shifts.

## Abstract 2

Oscillations of core clock genes, including Clock (Clk), are essential for the generation of circadian rhythms. This study reveals that upstream open reading frames are enriched in Drosophila circadian genes, and that they repress Clk protein translation and thereby influence circadian gene expression and behavior.

## Introduction

Circadian rhythms govern approximately 24-hr cycles of various biological processes through a transcription–translation feedback loop (TTFL), a mechanism that is evolutionarily conserved across diverse taxa [1–4]. Circadian systems drive gene expression rhythms in thousands of genes, regulating fundamental cellular activities as well as complex behaviors and diseases, such as cell proliferation, cell signaling, metabolism, rest/activity, cancer, and neurodegeneration [5–7]. While the core TTFL components are well-characterized, post-transcriptional regulatory mechanisms ensuring precise circadian timing remain incompletely understood, particularly in terms of translational regulation—a critical layer of control that enables rapid protein-level adjustments without the delays inherent in transcription.

*Drosophila*, with its genetic tractability and conservation of key circadian components, serves as an unparalleled model providing profound insights that resonate across species, including humans [4,8–11]. The *Drosophila* TTFL centers on transcriptional activators CLOCK (CLK) and CYCLE (CYC) driving *period* (*per*) and *timeless* (*tim*) expression, whose protein products feedback to inhibit CLK/CYC activity [12–14]. Crucially, circadian precision requires strict stoichiometric control of these components. The overexpression of CLK–CYC increases their activity at *per* and *tim* E-box enhancers, consequently shortening the circadian rhythms [15,16]. Recent studies have revealed that the precision of circadian rhythms is regulated by multi-layered mechanisms beyond transcriptional modulation, which include the regulation of protein synthesis and post-translational modifications. For instance, the translation of *tim* and *per* mRNAs is facilitated by TWENTY-FOUR (TYF) and its activator, ATAXIN2, by promoting an association with polyadenylate-binding protein [17–20]. Additionally, the synthesis of CLK and TIM proteins is inhibited by the microRNAs *bantam* [21] and *mir-276a* [22], respectively, with the overexpression of either miRNA leading to prolonged periods or arrhythmicity [21–23]. Post-translational modifications (PTMs) also affect the stability and subsequent degradation of PER and TIM [1]. While recent advances have significantly enhanced our understanding of the regulatory complexity of the classical TTFL, the field has not yet completely elucidated the intricate network of components and interactions that govern the fine-scale regulation of circadian rhythms, even in *Drosophila*, the quintessential model for circadian biology.

Recent studies underscore the pivotal role of translational regulation, particularly upstream open reading frames (uORFs), in controlling circadian rhythms in organisms with divergent circadian architectures. uORFs, the short ORFs with start codons located within the 5′ untranslated regions (5' UTRs) of eukaryotic mRNAs, are widely distributed in eukaryotic genomes [24]. uORFs play a key role in suppressing translation initiation of the downstream coding sequence (CDS) in an mRNA by either sequestering ribosomes or competing for binding to translation initiation complexes [25–29]. In *Neurospora*, the central circadian clock component, *frequency* (*frq*), has two isoforms—large (l-FRQ) and small (s-FRQ)—whose balance is determined by temperature-dependent splicing and is essential for the temperature compensation of circadian rhythms. Thermosensitive trapping of scanning ribosomes at the uORFs of l-FRQ modulates FRQ protein levels in response to ambient temperatures, thus calibrating the circadian clock [30]. Similarly, TIMING OF CAB EXPRESSION 1 (TOC1), a core component of the *Arabidopsis* circadian clock, is regulated by uORFs within its mRNA leader sequence [31]. Although *Neurospora* and *Arabidopsis* share similar design principles in circadian rhythms as humans and flies, the proteins involved differ significantly in both structure and conservation [32–34]. Recent studies have shown that uORFs in the mouse core clock gene *Period2* (*Per2*) play a role in temperature entrainment of the circadian clock [35] and in regulating sleep [36]. However, no significant differences in the period and amplitude of circadian rhythms were observed between wild-type and *Per2* uORF mutant mice [36]. In addition, extensive ribosomal binding to uORFs in rhythmically expressed mRNAs has been observed in mammalian cells, yet the functional impacts of these uORFs in circadian regulation have not been characterized [37,38]. In summary, although research into the translational regulation by uORFs is burgeoning, the complete spectrum of regulatory functions of uORFs in circadian rhythm control remains to be elucidated. Moreover, the potential impacts of uORF-mediated regulation on physiological and pathological processes linked to circadian rhythms are currently elusive, while their delineations can help reveal how circadian rhythms adapt to environmental pressures.

Here, we show that circadian rhythm-related genes are significantly enriched with ultra-conserved uORFs in *Drosophila*. These uORFs, which demonstrate translational activity in our previously published translatome data [39], may therefore play a crucial role in modulating circadian rhythms. We then conducted an in-depth exploration of conserved and potentially functional uORFs in *Clk* of *Drosophila melanogaster* and presented evidence that these uORFs substantially inhibit protein translation in vitro and in vivo. Knocking out (KO) *Clk* uORFs amplifies daytime CLK protein levels and shortens the circadian period of locomotor rhythm. Furthermore, *Clk-*uORF-KO mutants manifest increased morning sleep, likely stemming from diminished dopaminergic activity. These mutants also exhibit defects in modulating sleep duration in response to photoperiodic shifts. Molecular investigations underscore the requirement of *Clk* uORFs for photoperiod-driven regulation of CLK protein, highlighting their central role in both circadian and circannual orchestrations. Intriguingly, the mutants also display decreased fecundity and diminished resilience to starvation, indicating that the ablation of *Clk* uORFs has extensive physiological consequences beyond regulating circadian rhythms and sleep.

## Results

### Circadian rhythm genes are more likely to be regulated by uORFs

**Fig 1:**
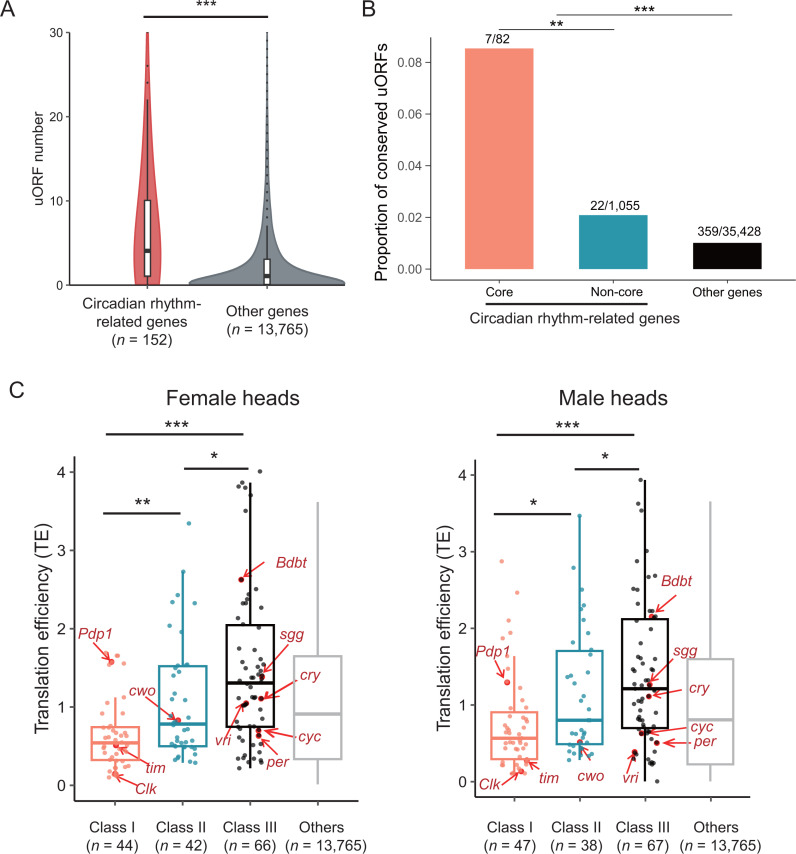
Circadian rhythm-related genes are more likely to be regulated by uORFs. **(A)** The distribution of uORF number in circadian rhythm-related genes and other genes in *Drosophila*. (The gene number (*n*) in each class are denoted at the bottom. Wilcoxon rank-sum test; *p < 0.05; ***p < 0.001). **(B)** Proportion of conserved uORFs in core clock genes, non-core circadian rhythm-related genes, and the other genes. Ratios reflect the number of conserved uORFs relative to the total count of uORFs for each category, depicted on each bar (Fisher’s exact test; **p < 0.01; ***p < 0.001). **(C)** The translational efficiency for different classes of circadian rhythm-related genes in male and female heads. Class I: genes with highly conserved uORFs (BLS ≥ 0.8) that are translated; Class II: genes with translated uORFs that are not highly conserved (BLS < 0.8); Class III: genes without translated uORFs. ‘Other’ encompasses all remaining genes expressed in heads that are not related to circadian rhythm. The gene counts of each class are denoted at the bottom of each bar. The 10 core clock genes are highlighted (Wilcoxon rank-sum test; *p < 0.05; **p < 0.01; ***p < 0.001). The data underlying this figure can be found in S1 Data.

To elucidate the role of uORFs in circadian rhythm regulation, we first performed a systematic analysis of uORFs distribution in *Drosophila* circadian genes. We annotated 36,590 putative canonical uORFs (starting with AUG and ending with a stop codon UAA/UAG/UGA) based on the 5' UTR annotation of 13,390 protein-coding genes in *D. melanogaster*, employing a method previously described [24,39] (S1 Table; Methods).To examine whether uORFs are more prevalent in genes associated with circadian rhythms, we compiled a list of circadian rhythm-related genes following the methods described in previous studies [40–45] by using the gene ontology (GO) term GO:0007623, which represents explicitly “circadian rhythm”. This list comprises 152 protein-coding genes considered as circadian rhythm-related genes for the subsequent analysis. When considering all of these circadian rhythm-related genes, including those without uORFs (treated as having zero uORFs), the number of uORFs per gene was significantly higher compared to other genes [*p* = 2.64 × 10^−23^, two-sided Wilcoxon rank-sum test (WRST); Fig 1A and S2 Table], demonstrating a strong enrichment of uORFs in genes involved in circadian rhythm. Since conserved uORFs are more likely to be functional [24,25,46], we then focused on uORFs highly conserved across *Drosophila* species. As the start codon (uATG) is the most pivotal and definitive feature of a canonical uORF [24,25], we investigated the conservation patterns of the uATGs based on multiple sequence alignments (MSA) of 23 *Drosophila* species. In total, we found 388 uATGs, within 250 genes, that are identical across the examined *Drosophila* species (S3 Table). These genes were significantly enriched for functions in neuronal axon genesis and transcriptional regulation, implicating the potential roles of uORFs in modulating these processes (S1A Fig). Notably, the circadian rhythm-related genes had a significantly higher proportion of highly conserved uATGs compared to other genes (29/1137 versus 359/35453; *p *= 2.3 × 10^−5^, Fisher’s exact test; Fig 1B). Among the 10 core clock genes (Table 1), 7 out of 82 uORFs had uATGs identical across *Drosophila* species, indicating higher uORF conservation compared to other circadian genes (7/82 versus 22/1055; *p *= 0.005, Fisher’s exact test; Fig 1B). These seven conserved uORFs were found in four genes, with *Pdp1* containing four, and *Clk*, *sgg*, and *cwo* each containing one (Table 1).

**Table 1:** uORFs in circadian rhythm-related genes.

Description	Circadian genes	Core genes	*Clk*	*sgg*	*cwo*	*Pdp1*	*tim*	*cyc*	*vri*	*cry*	*Bdbt*	*per*
Total uORFs	1,137	82	7	18	9	24	8	4	12	0	0	0
Conserved uORFs	29	7	1	1	1	4	0	0	0	0	0	0
**Male head**												
Transcribed uORF	888	67	6	15	8	24	6	3	5	0	0	0
Translated uORFs	692	52	5	13	6	15	5	3	5	0	0	0
Conserved translated uORFs	25	7	1	1	1	4	0	0	0	0	0	0
**Female head**												
Transcribed uORF	879	63	6	15	8	24	5	3	2	0	0	0
Translated uORFs	714	49	6	13	5	16	5	3	1	0	0	0
Conserved translated uORFs	25	6	1	1	0	4	0	0	0	0	0	0

The number of total uORFs or conserved uORFs in the circadian rhythm-related genes and 10 core clock genes, as well as each core clock gene, is shown. The number of transcribed uORFs (uORF with mRNA RPKM > 0.1) and translated uORFs (uORFs with mRNA RPKM > 0.1 and translation efficiency > 0.1) in the heads of male and female flies is shown. Conserved uORFs refer to the uATGs of uORFs that are present in all 23 *Drosophila* species. RPKM, reads per kilobase of transcript per million mapped reads.

In addition to comparing the proportion of conserved uORFs across gene categories, we also quantitatively assessed the conservation of uATGs by calculating their branch length score (BLS) across 23 *Drosophila* species as previously described [47]. A larger BLS value indicates a higher degree of conservation. uATGs in circadian rhythm-related genes exhibited significantly higher BLS values than the other genes (*p* = 8.58 × 10^−16^, WRST; S1B Fig), with core clock genes displaying even higher conservation (core versus non-core clock genes, *p *= 0.016; core clock genes versus other non-clock genes, *p *= 3.21 × 10^−6^; WRST; S1B Fig). As clock genes often have multiple isoforms with distinct 5' UTRs [48–50], we also restricted our analysis to the most abundant isoform per gene (estimated by kallisto [51]) and observed similar enrichment and conservation patterns (enrichment: *p *= 1.44 × 10^−14^, WRST, S2A Fig; conservation: core clock genes versus other non-clock genes, *p *= 0.04; non-core clock genes versus other non-clock genes, *p *= 2.96 × 10^−12^; WRST, S2B Fig). These findings suggest that circadian rhythm-related genes, particularly core clock genes, are more likely to be regulated by evolutionarily conserved uORFs.

Besides conservation, translation activity is also an indicator of the functional roles of uORFs. We analyzed previously published mRNA-Seq and Ribo-Seq data from *D. melanogaster* heads to quantify the translation efficiency of uORFs and CDSs [39]. Translational efficiency refers to the rate at which ribosomes translate mRNA into proteins, typically quantified by comparing the abundance of ribosome-protected mRNA fragments (RPF) to total mRNA levels (Methods). Among the uORFs expressed in male and female heads (mRNA RPKM > 0.1), about 80% (692/888 in males, 714/879 in females) showed evidence of translation (translational efficiency > 0.1, Table 1). Notably, 25 of the 29 highly conserved uORFs exhibited translation signals in both sexes. For core clock genes, about 78% of the expressed uORFs (52/67 in males and 49/63 in females) showed translation signals. These data suggest a potential regulatory role of uORFs in the translation of circadian rhythm-related genes.

To further assess the impact of uORFs on translation efficiency of the downstream CDS, we categorized the expressed circadian rhythm-related genes (149 in male and 147 female heads) into three groups based on uORF conservation and translation: Class I [genes with translated uORFs that are highly conserved uORFs (BLS ≥ 0.8)], Class II [genes with translated uORFs that are less conserved (BLS < 0.8)], and Class III (genes without translated uORFs). A statistically significant trend in the translational efficiency of CDS was observed across these classes: Class I < Class II < Class III (Fig 1C). These results suggest that the translation of uORFs, particularly the evolutionarily conserved ones, suppress the translation of downstream CDS. Notably, four core clock genes—*Clk*, *cyc*, *per*, and *tim*—showed reduced translation efficiency compared to the genomic average, with *Clk*, a Class I gene, exhibiting the lowest translation efficiency relative to other clock genes, pointing to a significant regulatory role of its uORFs on translation (Fig 1C).

### uORFs suppress CLK and CYC translation in vitro and in vivo

**Fig 2:**
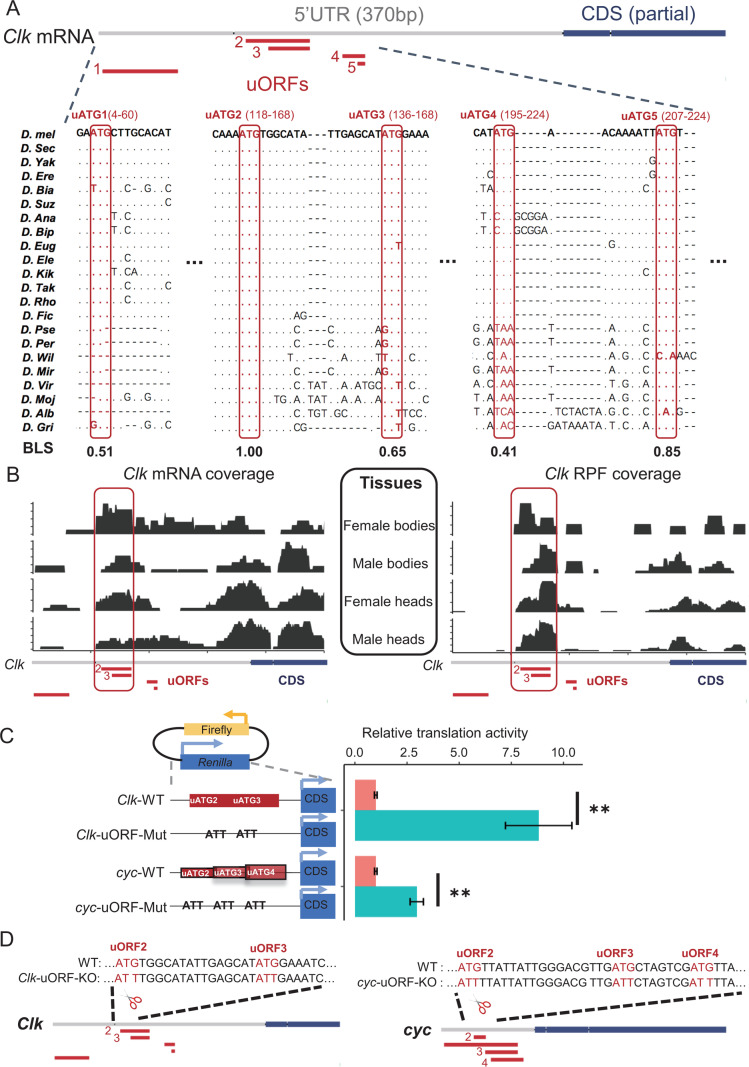
Regulation of CLK and CYC translation by uORFs in *Drosophila.* **(A)** Multiple sequence alignment (MSA) of *Clk* uORFs among 23 *Drosophila* species. The start codons (uATGs) of uORFs are highlighted by red boxes. The position schemes of uORFs and partial CDSs are denoted above the MSA, with red and blue colors, respectively. The start and end positions of each uORF (separated by “–”) in the 5' UTR are given in the parentheses above each uATG. The BLS score of each uATG was denoted below MSA. **(B)** The mRNA reads coverage (left) and ribosome-protected footprints (RPF) coverage (right) of uORFs of *Clk* mRNA from bodies and heads of both sexes. The position schemes of uORFs and partial CDSs were denoted at the bottom. **(C)** Relative translation efficiencies measured by dual-luciferase assays conducted on WT and uORF-mutated 5' UTRs of *Clk* and *cyc* genes in *Drosophila* S2 cells, with reporter constructs depicted top left. For the uORF mutants, the start codons of *Clk* uORF2 and uORF3, as well as *cyc* uORF2, uORF3, and uORF4, were changed from ATGs to ATTs. Relative translation efficiencies were calculated by normalizing the luminescence intensity to the mRNA level and the relative translation efficiencies in WT normalized to 1. Six replicates were conducted for each experiment. Data represent mean ± SEM (Two-tailed Student *t* test; **p < 0.01). **(D)** Diagrams depicting WT and two uORF knockout strains (*Clk*-uORF-KO and *cyc*-uORF-KO), created by using CRISPR-Cas9. For the uORF mutants, the start codons of *Clk* uORF2 and uORF3, as well as *cyc* uORF2, uORF3, and uORF4, were simultaneously changed from ATGs to ATTs. Below each sequence, uORFs (red boxes) and partial CDSs (blue boxes) are shown. The data underlying this figure can be found in S1 Data.

Given that *Clk* is the only core clock gene harboring highly conserved and translated uORFs that appear to strongly suppress its CDS translation, we focused on *Clk* uORFs. The 5' UTR of *D. melanogaster Clk* mRNA contains five canonical uORFs, with the second (uORF2) and third (uORF3) being in-frame and overlapping (Fig 2A). Among these, the start codon of uORF2 (uATG2) is the most conserved, identical across all 23 *Drosophila* species (BLS = 1) (Figs 2A and S1C). As CYC is the binding partner of CLK, we also analyzed *cyc* gene for comparison. The 5' UTR of *D. melanogaster cyc* mRNA contains four canonical uORFs, but their start codons are generally less conserved than those of *Clk*, with none being identical across the 23 *Drosophila* species (S3A Fig). We observed abundant mRNA expression and ribosome occupancy for uORF2 and uORF3 of *Clk* in both male and female heads and bodies (Fig 2B). As for *cyc*, although mRNA expression was observed for all four uORFs, uORF2 likely had the most pronounced ribosome occupancy (S3B Fig).

To explore the regulatory impact of uORFs on CDS translation, we performed a dual-luciferase reporter assay in vitro using wild-type (WT) and mutated 5' UTRs of *Clk* and *cyc* mRNAs, where the start codons for uORF2 and uORF3 of *Clk* and uORF2, uORF3, and uORF4 of *cyc* were mutated from ATGs to ATTs (Fig 2C). In *Drosophila* S2 cells, in addition to luminescence intensity, we also measured the mRNA level for each sample to exclude the influence of mRNA expression or degradation (S4 Fig). We observed that the relative translation output, calculated by normalizing the luminescence intensity to the mRNA level, was much higher for constructs with uATG-mutated 5' UTRs compared to WT controls (*Clk*^*WT*^ versus *Clk*^*mutant*^: *p *= 0.0022; *cyc*^*WT*^ versus *cyc*^*mutant*^: *p *= 0.0014; WRST) (Fig 2C). These results confirmed the repressive function of these uORFs on the translation of CDSs in *Clk* and *cyc* mRNAs.

**Fig 3:**
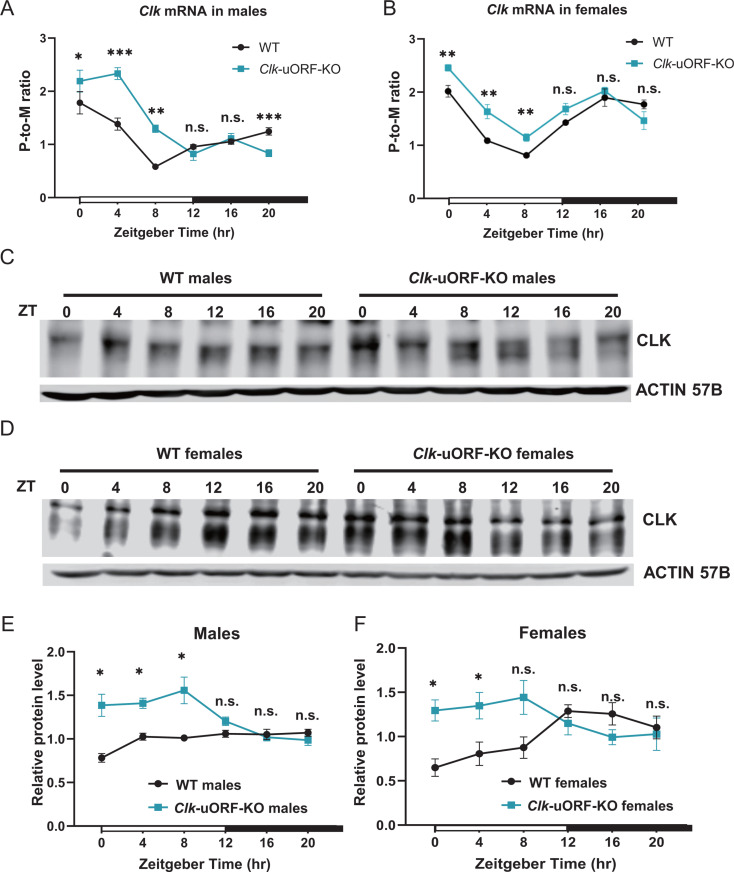
*Clk* uORFs suppress CLK protein translation in fly heads in vivo. **(A)** P-to-M ratios of *Clk* mRNA from whole heads of male *Clk*-uORF-KO compared to that in male WT flies, sampled at indicated Zeitgeber times at 4-hr intervals. Data are expressed as mean ± SEM (*n* = 4; Wilcoxon signed-rank test; *p < 0.05; **p < 0.01; ***p < 0.001; n.s., *p *> 0.05). **(B)** P-to-M ratios of *Clk* mRNA from whole heads of female *Clk*-uORF-KO compared to that in female WT flies, sampled at indicated Zeitgeber times at 4-hr intervals. Data are expressed as mean ± SEM (*n* = 6; Wilcoxon signed-rank test; **p < 0.01; n.s., *p *> 0.05). **(C)** Representative Western blots of CLK protein from whole-head protein extracts of male flies, sampled at indicated Zeitgeber times at 4-hr intervals. ACTIN 57B served as the loading control. **(D)** Representative Western blots of CLK protein from whole-head protein extracts of female flies, sampled at indicated Zeitgeber times at 4-hr intervals. ACTIN 57B served as the loading control. **(E)** Quantification of CLK abundance relative to ACTIN 57B from **(C)**, with the average intensity for WT normalized to 1 at each Zeitgeber time point. Data are presented as mean ± SEM (*n* = 4; Two-tailed Mann–Whitney *U* test; *p < 0.05; n.s., *p *> 0.05). **(F)** Quantification of CLK abundance relative to ACTIN 57B from **(D)**, with the average intensity for WT normalized to 1 at each Zeitgeber time point. Data are presented as mean ± SEM (*n* = 4; Two-tailed Mann–Whitney *U* test; *p < 0.05; n.s., *p *> 0.05). The data underlying this figure can be found in S1 Data.

To investigate the function of *Clk* and *cyc* uORFs in vivo, we knocked out *Clk* uORF2 and uORF3 simultaneously in *D. melanogaster* by mutating their start codon ATGs to ATTs, and generated a homozygous knock-out line (*Clk-*uORF-KO) (Fig 2D). Similarly, we mutated the ATG start codons of uORF2–4 to ATTs in *cyc* and generated a homozygous knock-out line (*cyc-*uORF-KO) (Fig 2D). We carried out ribosome fractionation followed by quantitative PCR (qPCR) to assess the translational status of *Clk* and *cyc* mRNAs in flies maintained under 12-hr light/12-hr dark (12L12D) cycles. This technique separates mRNAs based on the number of bound ribosomes and enables the calculation of the polysome-to-monosome (P-to-M) ratio, which reflects translation efficiency (S5A Fig). A larger P-to-M ratio means more mRNAs are enriched in the polysome fractions and bound by more ribosomes, thus indicating higher translation efficiency [52–54]. We conducted experiments on heads from *Clk*-uORF-KO, *cyc*-uORF-KO, and WT flies at six Zeitgeber Time (ZT) points, starting from ZT0 (the onset of light period) to ZT20 at 4-hr intervals (S6 Fig). In male heads, the P-to-M ratio of *Clk* mRNA was significantly higher in *Clk*-uORF-KO flies at ZT0, ZT4, and ZT8, but showed no significant differences at ZT12 and ZT16, with a decrease at ZT20 (Fig 3A). The pattern in *Clk*-uORF-KO females mirrored that in males (Fig 3B). Conversely, the P-to-M ratio for *cyc* mRNA in *cyc*-uORF-KO flies was significantly higher only at ZT16 (S5B Fig). Overall, these patterns indicate *Clk* uORFs exert substantial repression on translation, especially during the light phase, while *cyc* uORFs exert modest effects, prompting a focus on *Clk* uORFs in subsequent analyses.

Next, we measured CLK protein abundance in heads at various Zeitgeber time points using immunoblotting (Fig 3C and 3D). CLK levels were significantly higher in male *Clk-*uORF-KO flies compared to WT during the morning hours (ZT0, ZT4, and ZT8; Fig 3E), with a similar pattern observed in females (Fig 3F). To determine whether this increase resulted from enhanced translation rather than reduced protein degradation, we treated fly heads with the proteasome inhibitor MG132. Notably, *Clk-*uORF-KO flies still exhibited elevated CLK protein levels (S7A–S7D Fig). Furthermore, no significant differences in CLK ubiquitination were detected between WT and *Clk*-uORF-KO flies (S7E and S7F Fig). These results support that uORFs repress CDS translation in *Clk* mRNAs rather than affecting protein degradation.

Given the well-established role of CLK phosphorylation rhythms in the cycling of CLK/CYC-driven transcription [55], we conducted additional experiments to profile CLK phosphorylation status. While we observed rhythmic CLK phosphorylation in both WT and *Clk*-uORF-KO flies, no significant differences were detected between the two genotypes (S8 Fig), suggesting *Clk* uORFs do not affect CLK phosphorylation status. We also assessed CLK abundance on the first day of constant darkness and found that CLK levels were significantly elevated during the earlier part of the subjective day (S9A–S9D Fig), mirroring the pattern under light/dark cycles, suggesting the translational repression mediated by *Clk* uORFs is independent of light. Furthermore, CLK level remained elevated in *Clk*-uORF-KO flies lacking *per* and *tim* (S9E–S9H Fig), indicating that the increased CLK level during the day in *Clk*-uORF-KO is not caused by the altered circadian phase. Together, these data demonstrate that *Clk* uORFs downregulated CLK protein translation during the daytime, with pronounced effects in the early hours.

Previous studies have demonstrated that the biogenesis of translation machinery is controlled by oscillating transcription and/or translation activities [7,38], with peak translation of several translational initiation factors occurring predominantly at midday in *Drosophila* [56]. Our previous proteomic data [57] further revealed that 24 proteins in the GO term “translation” (GO: 0006412) category exhibit rhythmic expression, most of which are ribosomal proteins and translational factors, with 19 peaking during the subjective day (S10 Fig). This rhythmicity of translation machinery components may provide instructive signals driving *Clk* translation, overriding uORF-mediated translational regulation at night. Thus, uORFs may play a “permissive” role in regulating *Clk* translation, influenced by “instructive” signals from the translational machinery.

### *Clk* uORFs modulate the pace of the circadian clock

To quantitatively assess the effects of *Clk*-uORF-KO on circadian rhythms, we utilized a mathematical model previously established for simulating the *Drosophila* molecular clock [58]. This model employs nonlinear ordinary differential equations to model the regulatory weights among mRNA and protein molecules within the circadian network, making it applicable for predicting the consequence of increasing *Clk* translation. By incrementing the regulatory weight from *Clk* mRNA to CLK protein from the baseline to higher values, we incorporated the anticipated enhancement in *Clk* translation due to the uORF KO into our model (S11A Fig).

The simulation indicated an immediate increase in CLK protein levels correlating with a higher regulatory weight from *Clk* mRNA to protein, mimicking the *Clk*-uORF-KO effect (S11B Fig). This, in turn, led to elevated levels of *per* and *tim* mRNA and protein (S11B and S11C Fig). In the simulations, we used the intervals between peaks of *tim* mRNA as a measure of circadian period length. The mean ± SEM of period length were 23.926 ± 0.003 hrs, 20.967 ± 0.078 hrs, 18.956 ± 0.061 hrs, and 17.494 ± 0.043 hrs for regulatory weights from *Clk* mRNA to CLK protein set at 0.026 (baseline), 0.027, 0.028, and 0.030, respectively (S11D and S11E Fig and S4 Table). These findings demonstrated a shortening of the circadian period as the regulatory weight increases, suggesting that enhanced *Clk* translation—resulting from the disruption of *Clk* uORFs—compresses the circadian period.

**Fig 4:**
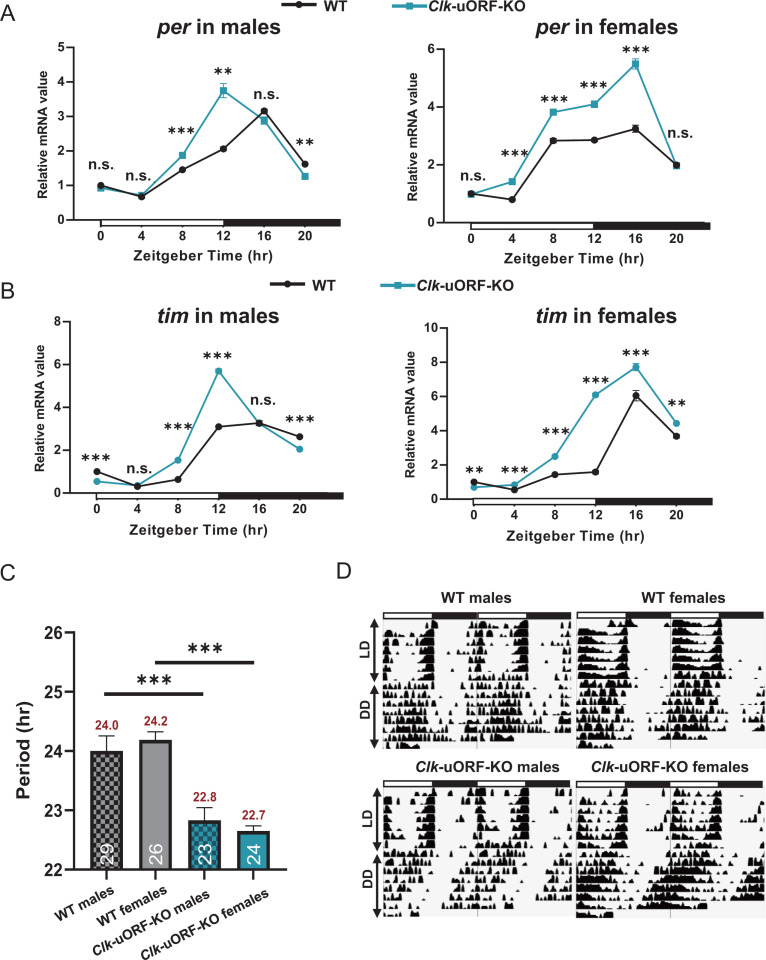
Experimental analysis of *Clk* uORFs on circadian rhythms. **(A and B)** RT-qPCR measured mRNA levels of *per* **(A)** and *tim* **(B)** from total RNA extracts of male and female heads, sampled at specified Zeitgeber time points (ZT). WT intensity at ZT0 is normalized to 1. Data are reported as the mean ± SEM (*n* = 4; Two-tailed Mann–Whitney *U* test; *p < 0.05; n.s., *p *> 0.05). **(C)** Period length of locomotor rhythms of *Clk*-uORF-KO flies and WT flies under constant darkness conditions (DD). The period lengths (hr) and the number of flies tested (*n*) are displayed above and below each bar, respectively. Data are reported as the mean ± SEM (Two-tailed Student *t* tes*t*; ***p < 0.001). **(D)** Representative double-plotted actograms of the locomotor activity of the indicated flies, monitored under LD followed by DD. White and black bars represent light and dark phases, respectively. The data underlying this figure can be found in S1 Data.

To corroborate the simulation outcomes, we measured the mRNA level of *per* and *tim*, observing significant elevation in both male and female *Clk-*uORF-KO fly heads (Fig 4A and 4B). As our simulations predicted, disrupting *Clk* uORFs reduced the period length of over one hour for the locomotor rhythm of *Clk*-uORF-KO flies. Specifically, the period length of WT males was 24.0 ± 0.25 hr, *Clk*-uORF-KO males 22.8 ± 0.21 hr, WT females 24.2 ± 0.14 hr, and *Clk*-uORF-KO females 22.7 ± 0.09 hr (Fig 4C and 4D and S5 Table). These empirical results, together with our simulation data, indicate that *Clk* uORFs modulated CLK translation, thereby impacting the pace of the molecular clock through the regulation of downstream genes such as *per* and *tim.*

### *Clk* uORFs promote morning wakefulness by tuning up the dopaminergic tone

**Fig 5:**
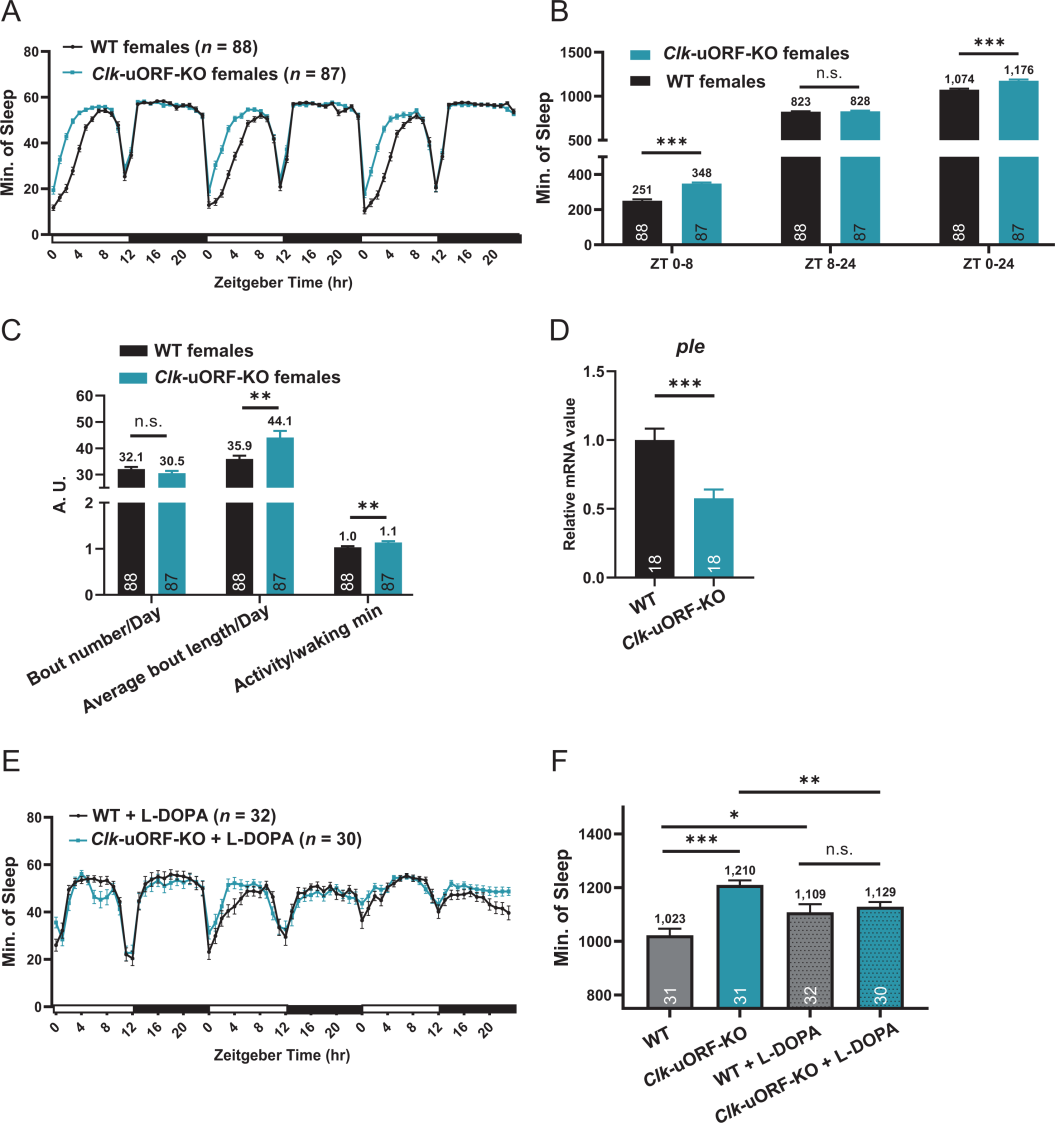
*Clk* uORFs regulate sleep/wakefulness. **(A)** Sleep profile of female *Clk-*uORF-KO and WT flies under LD conditions. **(B)** Sleep duration during ZT0–8, ZT8–24, and ZT0–24 of female *Clk-*uORF-KO and WT flies under LD condition. **(C)** Daily sleep bout number, average bout length, and average waking activity of female *Clk-*uORF-KO and WT flies under LD condition. **(D)** The relative mRNA abundance of *ple* determined by RT-qPCR using whole-head total RNA extracts collected from female flies under LD conditions. The average value of WT is set to 1. **(E)** Sleep profile of female *Clk-*uORF-KO and WT flies treated with L-DOPA under LD condition. **(F)** Daily sleep duration of female *Clk-*uORF-KO versus WT flies treated with L-DOPA. Sleep is monitored using the *Drosophila* activity monitoring system (DAMS) which is an infrared based detection system. The numbers of flies tested are shown in the brackets of legend or the bottom of histograms. Data are presented as the mean ± SEM. Asterisks indicate statistical significance (Two-tailed Student *t* test; *p < 0.05; **p < 0.01; ***p < 0.001; n.s., *p *> 0.05). ZT, Zeitgeber Time. The data underlying this figure can be found in S1 Data.

Given that the sleep-wake cycle is a key manifestation of circadian rhythms and *Clk* mutants exhibit significantly reduced sleep [59], we examined the impact of knocking out *Clk* uORFs on sleep under 12L12D cycles. Deletion of *Clk* uORFs significantly lengthened sleep duration, with a more pronounced effect in females (9.5% increase; Figs 5A and S12A) compared to males (4.0% increase; S12B and S12C Fig). A closer examination demonstrated that in *Clk*-uORF-KO female flies, sleep duration was extended by 38.65% during the first 8 hrs of the light phase, indicating that *Clk* uORFs facilitate morning wakefulness (Fig 5B). This increase in sleep duration was attributed to longer average sleep bouts, suggesting enhanced sleep continuity upon *Clk* uORF deletion (Fig 5C). Furthermore, waking activity levels were higher in female *Clk*-uORF-KO flies, ruling out impaired motor function as the cause of the prolonged sleep (Fig 5C). In contrast, the sleep alterations in *Clk*-uORF-KO males were less pronounced than in *Clk*-uORF-KO females (S12D Fig). We further validated the sleep phenotype in *Clk*-uORF-KO females using a video monitoring system we previously established [60], confirming a comparable increase in sleep during the morning hours (S12E and S12F Fig). Due to the more pronounced sleep phenotype in females, all subsequent sleep-related studies were conducted in female flies.

Given that CLK suppresses the expression of the gene encoding tyrosine hydroxylase (*ple*), which in turn diminishes dopamine signaling and activity levels [61], we investigated whether dopamine signaling mediates the effects of *Clk* uORFs on sleep. Our qPCR assays revealed a notable decrease in *ple* mRNA in female *Clk*-uORF-KO heads compared to WT (Fig 5D), aligning with the hypothesis that elevated CLK protein levels—resulting from *Clk* uORF disruption—downregulates *ple* expression. To further explore this, we treated female flies with dopamine precursor L-DOPA, which facilitates dopamine synthesis [62]. Interestingly, this treatment reversed the prolonged sleep duration in female *Clk-*uORF-KO flies (Fig 5E and 5F). Thus, *Clk* uORFs appeared to promote morning alertness by modulating dopaminergic signaling.

### *Clk* uORFs modulate sleep/wakefulness in adaptation to seasonal photoperiodic changes by regulating CLK protein translation in a photoperiod-dependent manner

**Fig 6:**
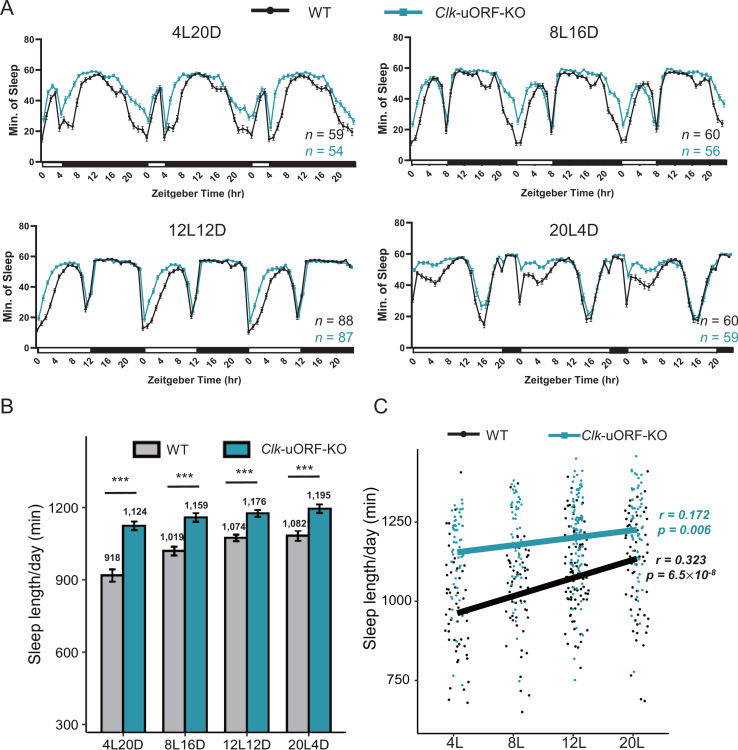
*Clk* uORFs promote wakefulness in adaptation to seasonal photoperiod changes. **(A)** Sleep profile of female *Clk-*uORF-KO and WT flies under 4-hr L:20-hr D (4L20D), 8-hr L:16-hr D (8L16D), 12-hr L:12-hr D (12L12D), and 20-hr L:4-hr D (20L4D) condition. The numbers of tested flies (*n*) are shown in the bottom-right. **(B)** Daily sleep duration (min) of female *Clk-*uORF-KO and WT flies under different photoperiods. Data are presented as the mean ± SEM. Asterisks indicate statistical significance (Two-tailed Student *t* test; ***p < 0.001). **(C)** The correlation between daily sleep duration (min) and photoperiod length in female *Clk-*uORF-KO and WT flies (Pearson’s correlation). Sleep is monitored using the DAMS which is an infrared based detection system. The data underlying this figure can be found in S1 Data.

Given the critical role of the circadian clock in seasonal adaptation [63], we hypothesized that uORFs might regulate *Clk* translation on a circannual basis. To test this hypothesis, we subjected *Clk-*uORF-KO and WT female flies to conditions mimicking seasonal changes. Because the most important seasonal signal for the circadian clock is the shortening/lengthening of photoperiod [63], we monitored locomotor rhythm and sleep under photoperiods of different lengths, ranging from 4 hrs of light and 20 hrs of dark (4L20D) to 20 hrs of light and 4 hrs of dark (20L4D). While *Clk* uORFs did not influence the timing of locomotor activity rhythm across photoperiods (S13 Fig), sleep duration exhibited a photoperiod-dependent pattern (Fig 6A). The difference in sleep duration between *Clk*-uORF-KO and WT females increased as photoperiods shortened (Fig 6B). Under 4L20D, *Clk*-uORF-KO females exhibited a 22.44% increase in sleep duration relative to WT (1,124 ± 18 min versus 918 ± 26 min), whereas this difference was reduced to 10.44% under 20L4D (1,195 ± 18 min versus 1,082 ± 21 min). Additionally, in WT females, sleep duration positively correlated with photoperiod length (Pearson’s *r* = 0.323, *p* = 6.5 × 10^−8^), but this correlation was weaker in *Clk*-uORF-KO females (Pearson’s *r* = 0.172, *p* = 0.006) (Fig 6C). Collectively, these findings suggest that *Clk* uORFs modulate sleep responses to seasonal changes, likely through translational regulation that fine-tunes sleep duration in response to photoperiod length.

**Fig 7:**
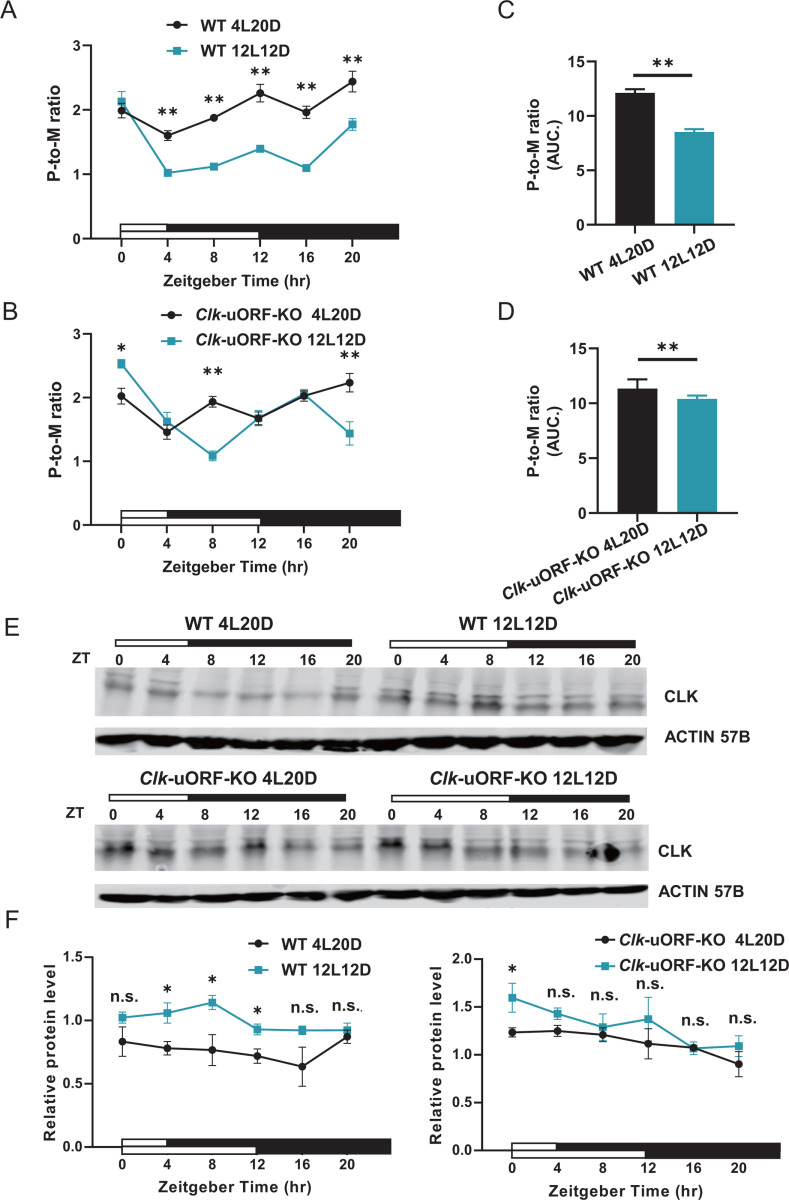
*Clk* uORFs suppress CLK protein translation in a photoperiod-dependent manner. **(A and B)** P-to-M ratios of *Clk* mRNA from whole-head extracts collected from female WT **(A)** and *Clk-*uORF-KO **(B)** flies at indicated time points under 4L20D and 12L12D conditions. Data are presented as the mean ± SEM (*n* = 6; Wilcoxon signed-rank test; **p < 0.01). **(C and D)** AUC analysis of daily P-to-M ratio in **(A)** and **(B)** of female WT **(C)** and *Clk-*uORF-KO **(D)** flies under indicated photoperiod. **(E)** Representative Western blots for CLK protein from whole-head total protein extracts collected from female WT (upper) and *Clk-*uORF-KO (lower) flies at indicated time points under 4L20D (left) and 12L12D (right). ACTIN 57B is used as a loading control. **(F)** The relative CLK abundance normalized to ACTIN 57B in (**E)**. The average value of WT under 12L12D is set to 1. Data are presented as the mean ± SEM. Asterisks indicate statistical significance (*n* = 4; Two-tailed Mann–Whitney *U* test for unpaired comparisons; *p < 0.05; n.s., *p *> 0.05). ZT, Zeitgeber Time. The data underlying this figure can be found in S1 Data.

Given the observed behavioral changes, we hypothesized that *Clk* uORFs mediate translational regulation in a photoperiod-dependent manner. To test this, we performed ribosome fractionation followed by qPCR to assess *Clk* CDS translation under 4L20D and 12L12D light-dark cycles. In WT female heads, *Clk* CDS translation efficiency (P-to-M ratios) was significantly lower under 12L12D compared to 4L20D at five of six time points (except ZT0) (Fig 7A); however, in *Clk*-uORF-KO, a significant reduction was observed only at ZT8 and ZT20, while ZT0 displayed an opposite trend compared to WT (Fig 7B). Consistently, overall CLK protein synthesis, measured as the area under the curve (AUC), was significantly reduced (approximately 30%) in WT under 12L12D compared to 4L20D (Fig 7C), whereas this reduction was minimal (approximately 8%) in *Clk-*uORF-KO flies (Fig 7D). These results demonstrate that the loss of *Clk* uORFs diminished translational regulation in response to changes in photoperiod length. Supporting this notion, a previous study identified 12 genes involved in translational regulation that exhibit photoperiod-dependent expression (16L8D versus 8L16D) [64]. We tested these genes and confirmed that 3 (*RpS4*, *RpL34a*, and *RpL14*) were differentially expressed under 4L20D relative to 12L12D (S14 Fig), implying that ribosomal proteins and other translational factors participate in adapting to photoperiod variation.

Despite higher *Clk* mRNA translation under 4L20D compared to 12L12D in WT flies, western blot analysis paradoxically revealed lower CLK protein levels under 4L20D—particularly from ZT4 to ZT12 (Fig 7E and 7F). In *Clk*-uORF-KO mutants, however, this reduction was only significant at ZT0 (Fig 7E and 7F). Moreover, the AUC analysis demonstrated that photoperiod changes significantly altered the total daily CLK level in WT but not in *Clk*-uORF-KO heads (S15 Fig). qPCR analysis showed that *Clk* mRNA levels were lower under 4L20D than 12L12D in both WT and *Clk-*uORF-KO (S16 Fig), which could contribute to the reduction of CLK protein level under 4L20D.

Together, these findings suggest that *Drosophila* integrate photoperiodic cues at multiple regulatory levels to facilitate seasonal adjustments in sleep/wakefulness via *Clk*, with *Clk* uORFs playing an essential role in this process.

### *Clk* uORFs influence the temporal transcriptomic landscape

CLK modulates the cyclical expression of many genes in *Drosophila* [64]. To investigate the impact of *Clk* uORFs on global gene expression rhythms, we performed mRNA sequencing on WT and *Clk*-uORF-KO fly heads collected at four-hr intervals under 12L12D cycles (three biological replicates per time point). Compared to WT females, *Clk*-uORF-KO females exhibited 625–1,422 differentially expressed genes (DEGs) per time point, while *Clk*-uORF-KO males showed 461–2,825 DEGs per time point. We observed a significant upregulation of *tim* and *per* expression in both male and female mutants (S17 Fig), consistent with the expression profiles detected by qPCR (Fig 4A and 4B).

**Fig 8:**
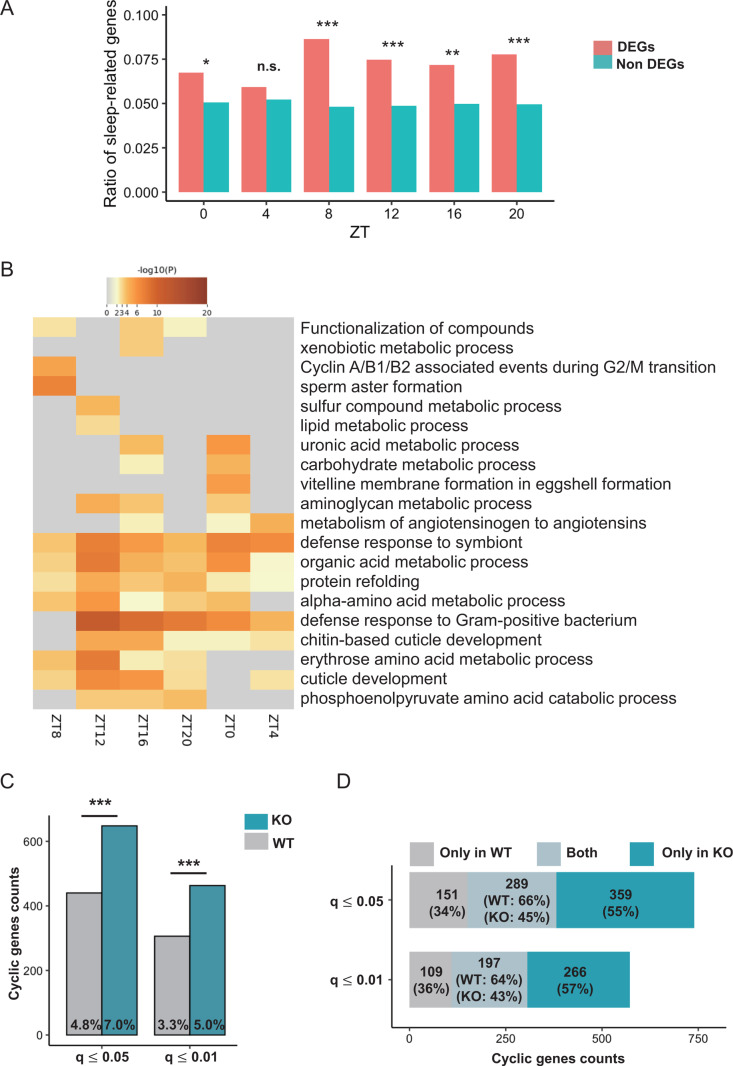
*Clk* uORFs modulate the temporal pattern of transcriptome expression landscape in female fly heads. **(A)** The ratio of sleep-related genes among differentially expressed genes (DEGs) and non-DEGs at each time point in female heads. Fisher’s exact tests were applied for comparisons at each time point. n.s., *p *> 0.05; *p < 0.05; **p < 0.01; ***p < 0.001. **(B)** Heatmap showing the GO terms enriched in DEGs for each time point. The heatmap cells were colored by their *p*-values, while gray cells indicate lack of enrichment for that term in the corresponding gene sets. **(C)** Number of cycling genes from whole-heads RNA-seq data of female WT and *Clk*-uORF-KO flies were determined by MetaCycle under two *q*-value cutoffs (JTK and ARS, FDR [*q* value] ≤ 0.05 or 0.01). The percentages of cycling genes out of a total of 9,202 expressed genes were indicated at the bottom of each bar. Fisher’s exact test; ***p < 0.001. **(D)** Number of shared cycling genes between WT and *Clk*-uORF-KO mutants in females under two *q*-value cutoffs (*q* value ≤ 0.05, or 0.01). The percentage is also indicated below the gene counts. KO, *Clk*-uORF-KO mutants. The data underlying this figure can be found in S1 Data.

Given the pronounced sleep alterations in *Clk*-uORF-KO mutants, we examined whether these DEGs were enriched for sleep-associated genes. We curated a list of sleep-related genes from the gene ontology term “sleep” (GO:0030431) and previous literature, including genes associated with sleep [60] and sleep latency [65], yielding 535 unique sleep-related genes (S6 Table). In females, we found a significantly higher proportion of sleep-related genes among DEGs compared to non-DEGs at 5 out of the 6 time points analyzed (*p *= 0.035, 0.45, 1.01 × 10^−5^, 4.30 × 10^−4^, 4.79 × 10^−3^, 7.39 × 10^−4^ for ZT0, 4, 8, 12, 16 and 20, respectively; Fisher’s exact test; Fig 8A), suggesting that *Clk* uORF deletion profoundly affects sleep-related gene expression. In contrast, this enrichment was weaker in males, reaching statistical significance only at ZT0 (*p* = 1.47 × 10^−7^) and ZT16 (*p* = 0.027; S18A Fig), consistent with the less pronounced sleep alterations in males. Moreover, gene ontology (GO) analysis revealed that DEGs in *Clk-*uORF-KO females were primarily enriched in metabolic pathways (Fig 8B). In males, DEGs were also enriched in metabolism pathways, though these enrichments were sparse across time points (S18B Fig). Together, these findings suggest that *Clk* uORFs broadly influence the expression of numerous genes involved in diverse biological processes beyond circadian rhythms and sleep.

To assess the impact of *Clk* uORFs on gene expression rhythms, we identified cycling genes with rhythmic expression using ARSER [66] and JTK_CYCLE [67] algorithms implemented in MetaCycle [68]. To ensure robust identification of rhythmic genes, we applied stringent false discovery rate (FDR) thresholds, requiring *q* values to be below 0.05 or 0.01 in both algorithms (S7 Table; Methods). Additionally, we incorporated an amplitude threshold (max/min fold-change ≥ 1.5) to account for both statistical and biological significance, consistent with previous studies [69]. Applying *q*-value thresholds of ≤0.05 or ≤0.01, we found that *Clk*-uORF-KO females consistently exhibited significantly more cycling genes than WT females (*p* = 8.70 × 10^−11^ and 8.14 × 10^−9^, respectively; Fisher’s exact test; Fig 8C). In contrast, *Clk*-uORF-KO males exhibited a slight increase in cycling genes compared to WT males, but this was not statistically significant (S18C Fig). Only 40 to 60% of cycling genes overlapped between WT and *Clk*-uORF-KO flies, a trend consistent across different *q*-value cutoffs (Figs 8D and S18D). Using *q* ≤ 0.05, we identified 440 rhythmic genes in WT females and 648 in *Clk*-uORF-KO females, with 289 shared genes (Fig 8D). In males, 472 genes were rhythmic in WT and 512 in *Clk*-uORF-KO, with 230 shared genes (S18D Fig). The circadian rhythm-related genes we compiled earlier were significantly enriched among cycling genes in WT (*p* = 0.0008 for females, *p* = 0.002 for males; Fisher’s exact test; S19A Fig), while this enrichment was reduced in *Clk*-uORF-KO (*p* = 0.014 for females, *p* = 0.048 for males; S19A Fig), further supporting that the removal of *Clk* uORFs disrupted the rhythmic expression of many circadian rhythm-related genes. Moreover, knocking out *Clk* uORFs increased the relative oscillation amplitude (rAMP), with a more pronounced effect in females than in males (*p* = 4.74 × 10^−15^ in females versus *p *= 1.21 × 10^−5^ in males; Wilcoxon signed-rank test; S19B Fig).

Cross-sex comparisons revealed that only approximately 50% of cycling genes were shared between WT male and female heads (S20A Fig), consistent with prior findings in *Drosophila* [70], mice [71], and humans [72,73]. This baseline sex-specificity extended to *Clk-*uORF-KO mutants, where only approximately 50% of cycling genes overlapped between male and female heads (S20B Fig), and a few genes that lost or gained rhythmicity in mutants were shared across sexes (S20C and S20D Fig). Nevertheless, GO analysis of these genes that lost or gained expression rhythmicity showed some similarities between males and females, with enrichment in metabolism pathways, though specific terms varied (S20E Fig). Overall, these findings indicate that *Clk* uORF deletion disrupted rhythmic expression in a large set of genes while inducing rhythmicity in hundreds of others, in a sex-dependent manner.

### Knocking out *Clk* uORFs impairs fecundity and starvation resistance

Transcriptome analysis revealed that numerous genes with altered expression and/or rhythmicity were enriched in several essential physiological pathways, primarily converging on metabolic processes. Since metabolism is fundamental to all physiological functions, we hypothesized that its disruption could ultimately manifest in *Drosophila* adaptation and fitness. Indeed, the *Clk*-uORF-KO flies showed significantly faster mortality rates under starvation (a metabolic stress) compared to WT flies for both sexes (*p* = 8.8 × 10^−13^ for females and *p *= 2.4 × 10^−9^ for males; log-rank test; S21 Fig). In addition, reproduction is intimately related with metabolic state [74,75]. We found that mated *Clk*-uORF-KO females laid significantly fewer eggs than their WT counterparts during an eight-day monitoring duration, with the total egg production of mutants markedly less (*p *< 0.01; S22A Fig). This pattern persisted in *Clk*-uORF-KO virgin females, which also laid fewer eggs than WT in both segmented (2-days) and pooled (10-days) measurements (S22B Fig). Accordingly, *Clk-*uORF-KO flies produced significantly fewer offspring than WT counterparts at 25°C (*p* = 1.5 × 10^−5^; WRST; S22C Fig). Even at 29°C, when both mutants and WT flies exhibited reduced offspring production compared to 25°C, the offspring counts for *Clk-*uORF-KO are still significantly fewer than that of WT (*p* = 0.00029, WRST; S22C Fig). Together, these results indicate that *Clk* uORFs profoundly affect fecundity and starvation resistance, with implications for fitness beyond their established roles in circadian rhythms and sleep regulation.

## Discussion

The evolutionary conservation of circadian mechanisms across species underscores their fundamental importance in regulating a wide range of physiological processes. While previous studies, including our own [57], have observed a low correlation between the temporal variation patterns of the transcriptome and proteome, suggesting a dominant role for translational and post-translational regulations in determining daily protein abundance variations, the precise mechanisms underlying these regulatory processes remain largely unexplored. This present study highlights the pivotal role of translational regulation, mediated explicitly by uORFs, in fine-tuning these rhythms. By generating *Clk*-uORF-KO flies, we demonstrated that the removal of these regulatory elements results in a compression of the circadian period by over one hour, a magnitude of change comparable to that observed when *Clk* is overexpressed using the UAS/GAL4 system [16]. Beyond circadian rhythms and sleep, *Clk-*uORF-KO flies show diminished fecundity and reduced starvation resilience, suggesting that *Clk* uORFs play essential roles in shaping the rhythmic expression of a vast array of genes and influencing multifaceted physiological outcomes. Moreover, our study uncovers a novel function of uORFs in modulating sleep/wake cycles in response to photoperiodic changes, a mechanism that has not been previously described. These findings advance our understanding of the complex regulatory networks governing circadian rhythms and beyond, providing new avenues for neurobiological research in this field.

**Fig 9:**
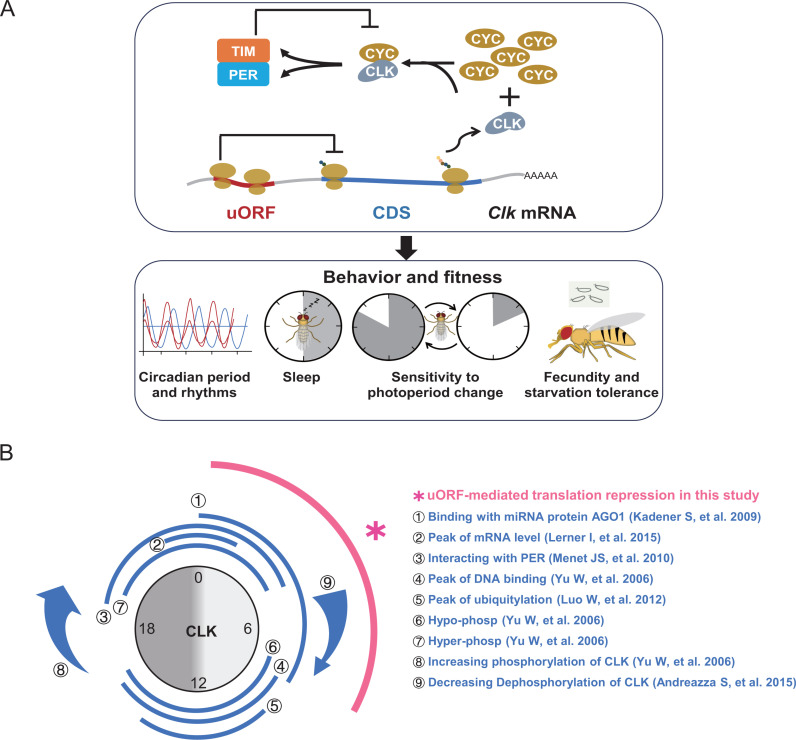
Model demonstrating the regulatory role of *Clk* uORFs within multi-layered mechanisms that control *Clk* expression and function. **(A)** Model illustrating the regulatory role of *Clk* uORFs on CLK protein translation and subsequent effects on behavior and fitness. CLK and CYC form heterodimers that activate the transcription of *per* and *tim*. PER and TIM proteins bind together and inhibit CLK–CYC activity, thus repressing their own transcription, creating the critical negative feedback loop. Note that CLK availability is a limiting factor for CLK–CYC heterodimer formation. The *Clk* uORFs are pivotal in regulating CLK stoichiometry, thus ensuring precise circadian timing. Deletion of *Clk* uORFs leads to increased *Clk* mRNA translation, enhancing CLK–CYC complex formation in the feedback loop and resulting in faster circadian cycles, which in turn affects the behaviors and fitness of flies. The diagram simplifies the concept by showing only one uORF on the *Clk* mRNA. **(B)** The regulatory mechanisms controlling *Clk* expression and function throughout the day summarized from previous studies [21,55,78–81] and this study. The uORF-mediated translational repression revealed in this study is highlighted in pink.

Within the *Drosophila* circadian clock, CYC is notably more abundant than CLK, with CLK being the limiting component in the formation of the CLK–CYC complex [76]. Our previous deep-sequencing data [39] confirm this stoichiometric relationship, with *cyc* exhibiting considerably higher abundance than *Clk* in both the transcriptome and translatome data from fly heads (S23 Fig). These findings support the notion that CLK availability dictates the abundance of the CLK–CYC heterodimer [76,77]. We propose a model in which *Clk* uORFs regulate CLK stoichiometry to ensure precise circadian rhythms, ultimately influencing physiology and fitness (Fig 9A). The removal of *Clk* uORFs leads to increased translation of *Clk* mRNA, elevating the level of the CLK–CYC complex within the core feedback loop and resulting in accelerated circadian cycles.

Previous studies have shown that CLK expression is achieved through diverse mechanisms, with different mechanisms dominant at various time points during the circadian rhythm period, such as *Clk* transcription [78], regulation by microRNAs [21], interaction with PER [79], binding DNA [55], ubiquitination [80], phosphorylation [55], and dephosphorylation [81]. Our study adds another layer to this complex regulatory network, showing that *Clk* uORFs suppress *Clk* mRNA translation primarily during the daytime (Fig 9B). Ribosomal proteins, along with other translational regulatory factors, exhibit rhythmic expression (S10 Fig). This could potentially contribute to the circadian regulation of *Clk* translation, possibly via uORF-mediated translational control. While further studies are needed to validate these findings, our results provide new insights into the design principles underlying circadian rhythm regulation.

The relationship between CLK overexpression and circadian period length in *Drosophila* has yielded inconsistent findings in the literature. Specifically, behavioral period length appears not significantly affected by *Clk* overexpression under the control of *per* enhancer sequences [77], whereas studies using the UAS/GAL4 system or *Clk*’s endogenous transcriptional regulatory elements have documented a shortened period length [15,16]. Our study contributes to this discussion by demonstrating that eliminating *Clk* uORFs shortens the circadian period by over an hour, reinforcing the notion that increased CLK levels correlate with a reduced period length. Of note, in this study, we backcrossed the *Clk* uORF-KO mutant strains with wild-type Canton-S strain for nine generations to minimize the potential influence of genetic background on phenotypes. Additionally, to further exclude background influences, we backcrossed the mutant flies with the isogenic *w*^*1118*^ strain for seven generations. The phenotypes of shortened period length, as well as increased sleep duration, remained consistent in this genetic background (S24 Fig). Furthermore, heterozygous mutants exhibit an intermediate increase in sleep duration compared to homozygotes and WT flies (S25 Fig). These findings confirm that CLK overexpression by deleting *Clk* uORFs shortens the circadian period and results in longer sleep duration, independent of genetic background.

To determine whether the shortened circadian period and increased sleep duration in the *Clk*-uORF-KO flies are caused by developmental defects, we performed immunostaining for pigment dispersing factor (PDF), a neuropeptide essential for regulating circadian rhythms in *Drosophila*. PDF is expressed in a subset of clock neurons called the small ventral lateral neurons (s-LNv), which send axonal projections to the dorsal region of the fly brain for maintaining proper circadian rhythms. Previous studies have shown that *Clk*^*jrk*^ mutants lacked the PDF+ dorsal projections from the s-LNv and displayed aberrant lateral projections from the large ventral lateral neurons (l-LNvs) [82]. In contrast, we found the PDF+ dorsal projections of the s-LNvs and l-LNvs in *Clk*-uORF-KO flies are comparable to that of WT (S26 Fig), suggesting that the altered circadian period and sleep duration are unlikely to stem from developmental defects in these key pacemaker neurons. However, we cannot exclude the possibility that developmental defects in other cells and tissues may contribute to the phenotypes observed in our study, such as altered starvation resistance and fecundity. Addressing this question would require tissue- or time-specific *Clk* uORF deletion, an approach that remains technically challenging and warrants further investigation beyond the scope of this study.

We observed widespread alterations in gene expression profiles in *Clk*-uORF-KO mutants under 12L12D cycles, with enrichment of sleep-related genes among differentially expressed genes, a pattern more pronounced in females than males. We also found that *Clk*-uORF-KO females exhibit significantly more cycling genes than WT females, whereas males showed only a slight increase. These findings suggest that increased CLK protein levels following *Clk* uORF deletion substantially rewire gene expression networks, with more pronounced molecular changes in females than males, consistent with the stronger sleep phenotype observed in female mutants. Additionally, genes with altered expression levels or rhythmicity were significantly enriched in metabolic pathways, suggesting broader physiological implications beyond circadian and sleep regulation. Given the intricate interplay between circadian regulation, metabolism, and adaptive fitness, it is not surprising that *Clk* uORF deletion also compromised fecundity and starvation resistance. However, these molecular changes may not be reflected in the locomotor rhythm and sleep phenotypes shown here in our study. Further experiments are needed to delineate the physiological consequences of the reorganization of gene regulatory networks following *Clk* uORF deletion.

Our study reveals that *Drosophila* circadian rhythm-related genes harbor more uORFs than the remaining genes, with these uORFs being more evolutionarily conserved across *Drosophila* species. Similarly, bioinformatic analyses indicated that in humans, uORFs are significantly enriched in circadian rhythm-related genes and are more conserved across mammalian species than other genes (S27 Fig). These patterns suggest that uORFs might be crucial in regulating circadian rhythms with functional implications for human health. Intriguingly, human individuals with seasonal affective disorder (SAD, commonly known as winter depression) report a winter increase in sleep, ranging from 30 min to 2 hr longer in duration compared to controls, while the underlying mechanism is yet unknown [83]. The extended sleep phenotype caused by knocking out *Clk* uORFs is most striking under a short photoperiod, reminiscent of the winter hypersomnolence in SAD. Notably, while we observed a stronger sleep phenotype in female flies, SAD is also more predominant in women [84]. From a mechanistic perspective, our results strongly suggest that *Clk* uORFs promote wakefulness by increasing tyrosine hydroxylase expression, thus up-regulating the dopaminergic tone. Similarly, the mammalian CLOCK protein has been shown to repress the transcription of *Tyrosine hydroxylase*, while *Clock* mutation leads to decreased sleep and depression-like behaviors in mice due to elevated dopamine signaling [85,86]. Recent single-cell RNA-seq analysis revealed minimal overlap between CLK^+^ neurons and dopaminergic neurons [87], indicating that CLK likely does not regulate *ple* in a cell-autonomous manner. Circadian clock neurons have been shown to project to protocerebral anterior medial (PAM) dopaminergic neurons and modulate their susceptibility to degeneration [88]. As PAM neurons are known to be involved in sleep/wake regulation, it is possible that CLK uORFs-mediated regulation might influence *ple* expression via this neural circuity. Taken together, our findings may provide some insights for understanding the pathological mechanism of SAD, and further studies are required in this direction.

To summarize, here we observed an enrichment of uORFs in circadian rhythm genes and further identified functional uORFs in *Clk* that repress the translation of CLK protein in both circadian and circannual manners. These regulations slow down the pace of the clock, promote morning wakefulness in adaption to seasonal photoperiod changes, and modulate global gene expression. This additional layer of complexity in the circadian regulatory network highlights the intricate mechanisms that have evolved to ensure the precise and robust control of biological rhythms in response to environmental cues. These findings, situated within the broader evolutionary, clinical, and interdisciplinary contexts, underscore the pivotal role of uORFs in shaping biological processes and potential therapeutic avenues.

## Methods

### Fly strains and cell line

All flies were reared on standard cornmeal-yeast-sucrose medium and kept in light/dark (LD) cycles at 25°C except for the specific experimental design denoted in the text. Food was accessible *ad libitum*. Flies aged 3 to 5-days were used for experiments. *{nos-Cas9}attP40* fly stocks used for embryo injection were from the Tsinghua Fly Center. The tool fly stocks (*y sc v* and *Dr, e/TM3, Sb*) used for mutant screening were obtained from the Tsinghua Fly Center*. Clk-*uORF-KO and *cyc*-uORF-KO mutants were generated and backcrossed to the Canton-S strain for nine generations to minimize the influence of genetic background. Throughout this study, homozygous *Clk-*uORF-KO or *cyc*-uORF-KO mutants were used, and the Canton-S strain served as the WT control flies unless otherwise stated. In addition, we backcrossed the *Clk-*uORF-KO mutant flies with the isogenic *w*^*1118*^ strain (BDSC:5905) for seven generations, and compared the resulting homozygous *Clk-*uORF-KO flies with the isogenic *w*^*1118*^ strain. This comparison ensured that the observed phenotypic effects in the mutants were not due to differences in genetic background. *Clk*^*Jrk*^ (BDSC:80927) were obtained from Bloomington *Drosophila* Stock Center (BDSC). Isogenic *Clk-*uORF-KO flies (*w*^*1118*^ strain) were crossed with isogenic *per*^0^ (BDSC:80917) and *tim*^*01*^(BDSC:80922) to generated homozygous triple knock-out flies (*per*^0^;*tim*^*01*^;*Clk-*uORF-KO). *Drosophila* S2 cells used in this study were purchased from Life Technologies Corp (http://www.lifetechnologies.com).

### The identification of uORFs

We downloaded the 100-way vertebrate genome alignments (maf) based on human (hg38) and 27-way insect alignments based on *D. melanogaster* (dm6), and the corresponding phylogenetic trees from UCSC Genome Browser (genome.ucsc.edu). We used the Galaxy platform [89] to parse the multiple sequence alignments of 5' UTRs in vertebrates or in *Drosophila*.

Leveraging the genome annotation of *D. melanogaster* (FlyBase r6.04, https://flybase.org/), the 5' UTR sequence of each annotated transcript of *D. melanogaster* and its corresponding sequences in other 22 *Drosophila* were extracted from the genome alignments. The start codons of putative uORFs were identified by scanning all the AUG triplets (uAUGs) within the 5' UTRs of *D. melanogaster*. uATG triplets that overlapped with any annotated CDS region were excluded, resulting in a total of 78,458 uORFs. After removing overlaps and merging uORFs with identical uATG genomic coordinates, 36,590 non-redundant uORFs remained. Similarly, the start codon of each uORF (uATG) was identified in humans and other vertebrate species based on the genome alignments.

### Branch length score (BLS) calculation of uAUGs

Each uAUG in *D. melanogaster* was determined the presence or absence in the orthologous sites in the other species based on multiple genome alignments. For each uAUG, we calculated the sum of the branch lengths of the subtree composed of the species in which the uAUG was present in the orthologous sites (B_*sum*_) and then calculated the BLS value by dividing B_*sum*_ by the total branch lengths for the phylogenetic tree of the 27-insect species. Similarly, the BLS was calculated for the start codon of each human uORF across 100 vertebrate species.

### Processing of ribosome profiling (Ribo-seq) data

The Ribo-seq data and matched RNA-seq data of *D. melanogaster* were obtained and processed from the previous study [39]. In brief, after removing 3' adaptors and quality controls, the NGS reads were mapped to the reference genome of *D. melanogaster* (FlyBase, r6.04) using STAR [90]. In each sample, we assigned a mapped RPF (27–34 nt in length) to its P-site and calculated the reads per kilobase per million (RPKM) values for a feature (CDS or uORF) with the mRNA or RPF data as: RPKM = 10^9^ × *n*/(*L × N*), where *n* is the number of reads that align to the feature, *L* is the length (nucleotides) of that feature, and library size *N* is the total number of reads uniquely mapped to the transcriptome. For uORFs that overlapped with CDSs, only the non-overlapping regions of the uORFs were used to calculate RPKM. The translation efficiency for a feature (CDS or uORF) was calculated as the ratio of RPF RPKM over mRNA RPKM [91].

### Generation of uORF-KO strain

We searched for possible sgRNA target sites near the start codon of the uORFs of *Clk* and *cyc* genes using the Benchling website (https://www.benchling.com/crispr/) to design optimal single guide RNA (sgRNA) sequences with high specificity and low off-target effects. Two pairs of sgRNAs were used for each gene. We then synthesized single-stranded complementary DNAs (ssDNAs) and annealed them to obtain double-stranded DNA (dsDNA), which served as the template for sgRNA expression. The dsDNA was then ligated into the BbsI-digested pU6B vector. To achieve single base editing (ATG to ATT) by CRISPR/Cas9-mediated Homology-Directed Repair (HDR), a plasmid DNA donor with about 2 kilobases flanking the targeted site was constructed following a previous protocol [92]. The donors and pU6B-sgRNA plasmid were co-injected into the embryos of transgenic Cas9 flies collected within one hour of laying at the Tsinghua Fly Center as described in *Ni* and colleagues [93]. The injected embryos were kept at 25°C and 60% humidity until adulthood (G0). The G0 adult flies hatched from the injected embryos were individually crossed with another strain (*y sc v*) to increase the number of offspring. Then, the F1 progeny were crossed with flies carrying an appropriate balancer (*Dr, e/TM3, Sb*). After F2 spawning, the F1 individuals were screened for mutations of interest by genotyping. The F2 progeny whose parents showed positive genotyping results were then screened for the *y*^−^ allele to separate the chromosome carrying *nos-Cas9*. The screened F2 males were crossed with flies containing the same balancer as mentioned above. After F2 genotyping, the progeny (F3) of positive F2 individuals carrying the same mutation were crossed individually to generate homozygous mutants in the F4 generation. The original homozygous mutants were backcrossed with Canton S flies for 9 generations or with *w*^*1118*^ for 7 generations, respectively. The template sequences of the sgRNA uATG-KO and primers used for genotyping are listed in S8 Table.

### uORF mutation sequencing validation

The mutant fly was homogenized with DNA sequencing extract buffer [10 mM Tris-HCl at pH 8.0, 1 mM EDTA at pH 8.0, 25 mM NaCl, and 0.02% proteinase K (CW2584, CWBIO)] and incubated at 37°C for 30 min. PCR was performed with Taq Plus MasterMix (CW2849, CWbiotech). The PCR reaction was performed as follows: 94°C for 2 min followed by 94°C for 10 s, 57°C for 15 s, and 72°C for 1 min 20 s for 35 cycles. The primers used are listed in S8 Table. PCR products were sequenced using commercial Sanger sequencing (AuGCT Co.).

### Cell culture and transfection

S2 cells were cultured in Schneider’s *Drosophila* Medium (Sigma) containing 10% heat-inactivated fetal bovine serum (FBS), 100 U/ml penicillin, and 100 μg/ml streptomycin (Thermo Fisher) at 25°C for 24 hr to reach 2–4 × 10^6^ cells/ml before further treatments. Plasmid transfection was conducted with Lipofectamine 3000 (L3000001, Thermo Fisher) according to the supplier’s protocol.

### Dual-luciferase assay

The wild-type (WT) 5' UTR of *Clk* and *cyc* was cloned from cDNA by PCR, and uATG mutations were introduced into 5' UTR by the amplification primers. The PCR primers used for this purpose are listed in S8 Table. The WT and mutated 5' UTR sequences were ligated into a linearized reporter plasmid, a modified psiCHECK-2 vector (Promega) with ORF codons of *Rluc* and *Fluc* optimized for *D. melanogaster*. The entire sequences of all the plasmids were validated by Sanger sequencing. The *Renilla* luciferase activity associated with WT or uORF-mutated 5' UTRs was measured according to the manual of the Dual-Luciferase Reporter Assay System (Promega) 32 hr after transfection and was normalized to the activity of firefly luciferase. The matched RT-qPCR for each dual-luciferase assay was performed with PowerUp SYBR Green Master Mix (Thermo Fisher) after reverse-transcribing RNA into cDNA using PrimeScript II first Strand cDNA Synthesis Kit (Takara). The qPCR primers used for this purpose are listed in S8 Table.

### Fly heads collection

Flies were collected within 3–7 days of eclosion and entrained in 12-hr L: 12-hr D (12L12D) at 25°C for three days. After that, flies were collected and frozen at −80°C. Frozen flies were vortexed for 10 s to separate the head from the body on dry ice.

### Ribosome fraction analysis

The heads obtained from WT and mutant flies were collected and homogenized with lysis buffer, the lysates were clarified by centrifugation at 4°C and 20,000 *g* for 10 min, and the supernatants were applied to sucrose gradient ultracentrifugation (see S1 Text for details). The RNA in the monosome and polysome fractions was extracted separately using TRIzol reagent (Life Technologies, Inc.) and chloroform (Beijing Chemical Works) following the manufacturer’s instructions and were reverse transcribed into cDNA using the PrimeScript II first Strand cDNA Synthesis Kit (Takara). RT-qPCR analysis of *Clk* and *cyc* cDNA was performed using PowerUp SYBR Green Master Mix (Thermo Fisher) following the manufacturer’s instructions. The primer sequences employed for RT-qPCR are listed in S8 Table. For each sample, the ratio of *Clk* and *cyc* mRNA abundance in the polysome fraction to that in the monosome fraction was calculated as the P-to-M ratio. Six biological replicates were performed for each sample.

### Western blot

Proteins were extracted from fly heads and cultured cells using SDS lysis buffer [50 mM Tris-HCl (pH 7.6), 150 mM NaCl, 1% Triton X-100, 0.5% SDS, 1 mM EDTA (pH 8.0), 2 mM DTT, 1× proteinase and phosphatase inhibitors (4693116001 and 04906845001, Roche)]. After homogenization, protein lysates were centrifuged at 12,000 *g* for 15 min at 4°C, then mixed with the 4× loading buffer [0.2 M Tris-HCl (pH 6.8), 8% SDS, 6 mM bromophenol blue, 4.3 M glycerol and 0.4 M DTT] and incubated at 95°C for 5 min. Equal amounts of protein were loaded into each well on 6% SDS–PAGE gels and then transferred to nitrocellulose membranes for 2 hr at 90 V. Membranes were incubated with primary antibody at 4°C overnight, followed by secondary antibody at room temperature for 1 hr. The primary antibodies used were as follows: guinea pig anti-CLK (1:1000, gift from Dr. Joanna Chiu) and rabbit anti-ACTIN 57B (1:5000, AC026, ABclonal, CN). Donkey secondary antibodies (1:10000 dilution) were conjugated either with IRDye 680 or IRDye 800 (926–68072 and 926–32213, LI-COR Biosciences, US). Bands were visualized and quantified with an Odyssey Infrared Imaging System (LI-COR Biosciences).

### Immunoprecipitation

For immunoprecipitation of fly heads, 150 mg fly heads were homogenized with 1 ml PBS buffer and then centrifuged at 5,000 *g* for 10 min at 4°C. Pellets were suspended with 500 μl IP buffer (50 mM Tris-HCl at pH 7.4, 150 mM NaCl, 1% NP-40, 0.1% SDS, 2 mM EDTA at pH 8.0, 0.15 mM spermine, 0.5 mM spermidine, 330 nM TSA, 10 mM Nicotinamide, 1× proteinase and phosphatase inhibitors). Homogenates were then sonicated for 18 × 5 s at 10% power and centrifuged at 17,000 *g* for 5 min at 4°C. Twenty microliters of 450 μl supernatant was used as input, and the remaining was divided into two parts. Five micrograms of CLK antibody or guinea pig normal IgG were added to the two parts. The samples were incubated at 4°C overnight, and then 50 μl of Protein A magnetic beads (Bio-Rad) were added, followed by 4 hr incubation at 4°C. Beads were washed with IP buffer for 3 × 10 min, and protein precipitates were eluted by SDS–PAGE loading buffer for Western blotting.

### Model simulation for the outcome of enhanced *Clk* translation

We adopted a previously published mathematical model of the *Drosophila* circadian clock [58]. This system relies on a nonlinear, autonomous, first-order system of ordinary differential equations to simulate the regulatory weights among mRNAs or protein molecules in the molecular clock network. Details of the simulation can be found in the (see S1 Text for details).

### Total RNA extraction and RT-qPCR

RNA was extracted from frozen fly heads maintained at −80°C. Fifty fly heads per sample were homogenized in Total RNA Isolation (TRIzol) Reagent (15596026, Invitrogen) by using a homogenizer. After mixing with trichloromethane, homogenates were centrifuged at 12,000 *g*, and the suspension was precipitated with 75% ethanol. After air drying, total RNA was reverse-transcribed into cDNA, and genomic DNA was removed with One-step cDNA Synthesis SuperMix (AT311, Transgen). Quantitative real-time PCR was performed with One-Step RT-PCR SuperMix (AT141, Transgen). The PCR reaction was performed as follows: 45°C for 5 min; 94°C for 2 min; 94°C for 5 s, 60°C for 15 s, 72°C for 20 s for 40 cycles (Applied Biosystems). The ΔΔCT method was used for quantification. *Beta-Actin* was used as an internal control. The primers used are listed in S8 Table.

### Locomotor activity monitoring and analysis

Locomotor activity levels of adult flies were monitored by *Drosophila* activity monitoring system (DAM system, TriKinetics) for 7 days of LD cycle at 25°C followed by 7 days of DD at 25°C. Flies were loaded into activity tubes containing 5% sucrose and 2% agar. The protocol of the DAM system was previously described [94]. For DD rhythmicity, chi-squared periodogram analyses were performed by Clocklab (Actimetrics). Rhythmic flies were defined as those in which the chi-squared power was ≥10 above the significance line. Period calculations considered only rhythmic flies. Dead flies were defined by 0 activity on DD7 and removed from the analysis.

### DAMS-based sleep measurement and analysis

*Drosophila* activity monitor system (Trikinetics) was used to record fly sleep. Flies were loaded into activity tubes containing 5% sucrose and 2% agar and entrained under LD at 25°C for 3 days, and then their activities in the next 3 days under LD conditions were recorded. Sleep is defined as 5 min consecutive inactivity. Sleep was analyzed with SleepMat following previously published protocol [95].

### Video tracking assay

Video capture and object tracking analysis of fruit flies were performed as previously described [60]. In brief, 3–5 days-old female flies were entrained for 1.5–2 days in 12-well plates lined with fly food containing 2% agar, 5% sucrose, and 0.1% propanoic acid, and sleep was monitored for 24.5 hr. Daily sleep was quantified by the video-to-location (VTL) method.

### PDF immunofluorescence and microscopy

Adult female flies were entrained for 3 days at 25°C and anesthetized with CO_2_. Brains were dissected in PBS buffer containing 3.7% formaldehyde. After fixation at room temperature for 30 min, the brains were rinsed for 10 min twice in PBS and incubated in PBS with 1% Triton for 30 min at room temperature. The brains were then incubated in 5% donkey serum diluted in PBT (PBS with 0.5% Triton) for 30 min at room temperature, followed by incubation in 1:50 mouse anti-PDF (DHSB, DF C7) antisera in PBT containing 5% donkey serum at 4°C. After PBT rinses for six times, the brains were incubated with 1:500 donkey anti-mouse AlexaFluor 647 (Thermo Fisher) for PDF immunostaining in PBT overnight at 4°C. After final rinses in PBT, brains were mounted in 80% glycerol diluted in PBS. PDF-labeled specimens were photographed with × 20 lens by Olympus FV3000 laser scanning confocal microscope (Olympus). The microscope, laser, and filter settings for a given experiment were held constant.

### Sholl analysis of the sLNv axonal arbor

The complexity of neuronal axon terminal projections was analyzed using the Sholl Analysis plugin in Image J. The center was defined as the inflection point of axonal curvature, with the maximum radius set to the farthest distal point of the axon. Concentric circles were drawn at 5 μm increments, and the number of intersections between the axon and the first 10 concentric circles was quantified. The average number of intersections across experimental groups was calculated to evaluate axonal branching complexity.

### Analysis of transcriptomic circadian rhythms in WT and mutant

We collected heads from *Clk-*uORF-KO mutants, and WT flies raised under LD cycles at 25°C at ZT 0, 4, 8, 12, 16, and 20. Library construction and sequencing with PE150 were conducted by Annoroad on the Illumina Nova6000 platform. Three biological replicates were sequenced for each time point. The clean data were mapped to the reference genome of *D. melanogaster* (FlyBase, r6.04) using STAR [90]. Reads mapped to the exons of each gene were tabulated with htseq-count [96]. Then the fragments per kilobase per million (FPKM) value was calculated for each gene as: FPKM = 10^9^ × *n*/(*L* × *N*), where *n* is the raw reads count that aligns to the gene, *L* is the merged length (nucleotides) of all exons of the gene, and library size *N* is the total number of reads uniquely mapped to the transcriptome. The genes with median FPKM ≥ 1 across six-time points were considered expressed genes and were further analyzed for rhythmic expression.

For a sample, we used DESeq2 [97] to determine the size factors of the library and normalized the mRNA read counts by dividing the raw counts with the corresponding size factors. Differential gene expression analysis was performed using DESeq2. We assumed genes with *p.* adjust < 0.05 as differentially expressed genes between WT and mutants. The normalized read counts were used to determine the circadian cycling using MetaCycle [68]. We used the two algorithms implemented in Metacycle, ARSER (ARS) [66] and JTK_CYCLE (JTK) [67], to identify cyclic genes. Significant cyclic genes were initially determined under two stringent levels of false discovery rate (FDR, *q* value) thresholds: *q* ≤ 0.05 and 0.01 in both JTK and ARSER, to evaluate the potential bias due to the selection of different *q*-value cutoffs. For the simplicity of the following analysis, a gene was determined to be significantly cycling if it had *q* ≤ 0.05 in both JTK and ARSER and a max/min fold-change ≥1.5, a criterion by a previous publication for rhythmicity detection [69]. We used the relative amplitude (rAMP) calculated by the MetaCycle to compare the amplitude of overlapped cyclic genes between WT and mutant.

### GO-based enrichment

Gene ontology (GO) enrichment analyses of the DEGs and genes that lost/gained rhythmic expression were performed by the Metascape platform (www.metascape.org) with a setting of the threshold at *p*. adjust < 0.05 [98].

### Drug treatment

For pharmacological experiments, 5 mg/ml L-DOPA (BBI Life Sciences) was mixed in the fly food (5% sucrose and 2% agar), and the flies were fed with or without L-DOPA food during sleep monitoring under LD at 25°C for 6 days.

### Egg numbers quantification

Newly hatched virgins were picked out and allowed to mature for two days in separate vials. They were then mated by placing one virgin female with three male flies for two days. Subsequently, 10 female parents from each group were transferred to a fresh dish containing a grape juice-based medium overlaid with yeast. This procedure was replicated in five separate dishes for each strain.

After one day of egg laying, the number of eggs deposited was meticulously documented. Following this assessment, the female parents were relocated to a new dish. This egg quantification process was conducted over eight days. For the virgin female, egg counts were performed every two days, extending over 10 days.

### Quantification of offspring number per female fly

Newly hatched virgins were picked out and allowed to mature for two days in individual vials. They were then mated by placing one virgin female with three male flies for two days. After that, each female parent was transferred to a new vial, and the number of offspring produced in 10 days was measured at 25 and 29°C. All assays were performed with 20 females per genotype.

### Measurement of starvation resistance in adult flies

We selected 3–5-day-old adult males and females and placed them in a starvation medium (1.5% agar), with 10 flies per vial and 10 vials for both males and females from each strain. We observed every 6 hrs or 12 hrs to count the number of deaths under starvation conditions until all flies starved to death. The survival curves were plotted using the ggsurvplot package in R.

### Quantification and statistical analysis

Quantifications in all data graphs represent the mean of at least four biological replicates, and error bars represent the standard error of the mean (SEM). Two-tailed Mann–Whitney *U* test, Wilcoxon rank-sum test, Wilcoxon signed-rank test, log-rank test, and two-tailed Student *t* test was used to calculate statistical significance. All statistical analysis was carried out using *Statskingdom* or R language. The number of biological replicates (*n*) and significant *p* values were noted in each statistical analysis in corresponding figure legends.

## Supporting information

S1 Fig The conservation of uATGs in clock genes. **(A)** GO-based enrichment analysis for genes containing uORFs conserved across 23 *Drosophila* species. **(B)** Empirical cumulative distribution function (ECDF) of the BLSs for uATGs in core clock genes, non-core clock genes and other non-clock genes, respectively. The ECDF curve represents the cumulative probability distribution of BLS values. A curve shifted to the left indicates smaller BLS values (lower conservation). The gene number in each class is denoted in parentheses. Wilcoxon rank-sum test; *p < 0.05; ***p < 0.001. **(C)** The BLS distribution of all uORFs, *Clk* uORFs and *cyc* uORFs. The five uORFs of *Clk* are marked as red dots, with the corresponding numbers labeled on the right side. The four uORFs of *cyc* are marked as blue dots, with the corresponding numbers labeled on the left side. The knocked-out uATGs are in boxes. Underlying data for this figure can be found in S1 Data.
(PDF)

S2 Fig The enrichment and conservation of uORFs in circadian rhythm-related genes when only the most abundant isoform was considered for each gene. **(A)** The distribution of uORF number in circadian rhythm-related genes and other genes in *Drosophila*. The gene number (*n*) in each class are denoted at the bottom. **(B)** ECDF of the BLSs for uATGs in core circadian clock genes, non-core clock genes and other genes, respectively. The gene number in each class is denoted in the parentheses. Notably, only the most abundant isoform for each gene is considered in the analysis. Wilcoxon rank-sum test; n.s., *p *> 0.05; *p < 0.05; ***p < 0.001. Underlying data for this figure can be found in S1 Data.
(PDF)

S3 Fig The conservation and translation of *cyc* uORFs in *D. melanogaster.* **(A)** MSA of *cyc* uORFs among 23 *Drosophila* species. The start codons (uATGs) of uORFs are highlighted by red boxes. The position schemes of uORFs and partial CDSs are denoted above the MSA, with red and blue colors respectively. The start and ending positions of each uORF (separated by “hyphen”) are given in the parenthesis above each uATG. The BLS of each uATG were denoted below MSA. **(B)** The mRNA reads coverage (left) and ribosome-protected footprints (RPF) coverage (right) of uORFs of *cyc* mRNA from the heads and bodies. The position schemes of uORFs and partial CDSs are denoted at the bottom. Underlying data for this figure can be found in S1 Data.
(PDF)

S4 Fig The luminescence intensity and mRNA abundance of dual-luciferase assay. **(A and B)** The luminescence intensity **(A)** and mRNA abundance **(B)** under the control of 5' UTR containing mutant uORFs (Mut) and WT 5' UTR of *Clk* or *cyc*, respectively. The relative mRNA abundance is measured by RT-qPCR. Data represent mean ± SEM (*n* = 6). In **(A)**, *p *= 0.0036 for *Clk* and *p *= 0.0022 for *cyc*. In **(B)**, *p *= 0.87 for *Clk* and *p *= 0.22 for *cyc*. Two-tailed Student *t* test; n.s., *p *> 0.05; **p < 0.01. Underlying data for this figure can be found in S1 Data.
(PDF)

S5 Fig Diagram of P-to-M ratio measurement. **(A)** Diagram illustrating the separation of monosomes and polysomes in a sucrose density gradient (10−45%). The P-to-M ratio is the ratio of mRNA abundance in the polysome fraction to that in the monosome fraction. (**B**) P-to-M ratios of *cyc* mRNA from whole heads of male *Clk*-uORF-KO compared to that in male WT flies, sampled at indicated Zeitgeber times at 4-hr intervals. Data are expressed as mean ± SEM (*n* = 6; Wilcoxon signed-rank test; *p < 0.05; **p < 0.01; ***p < 0.001; n.s., *p *> 0.05). Underlying data for this figure can be found in S1 Data.
(PDF)

S6 Fig Ribosome profiles measured by sucrose gradients. **(A and B)** Ribosome profiles of fly heads from WT, *Clk*-uORF-KO flies **(A)** and *cyc*-uORF-KO flies **(B)** at the indicated time points, measured by sucrose gradient. Underlying data for this figure can be found in S1 Data.
(PDF)

S7 Fig Knocking out *Clk* uORFs does not alter CLK degradation. **(A and C)** Representative Western blots of CLK protein from whole-head protein extracts of female and male flies fed with proteasome inhibitor MG132 (200 µM) for indicated hours prior to collection. ACTIN 57B served as the loading control. **(B and D)** Quantification of CLK abundance normalized to ACTIN 57B from **(A and C)**, with the average intensity for WT and *Clk*-uORF-KO normalized to 1 at 0 hr. Data are presented as mean ± SEM (*n* = 4; Two-tailed Mann–Whitney *U* test. *p < 0.05). **(E and F)** Immunoprecipitation assays were performed using protein extracts from female **(E)** and male **(F)** fly heads with anti-CLK antibody or IgG control. Precipitates were blotted with the indicated antibodies. IP, immunoprecipitation; IB, immunoblotting. Underlying data for this figure can be found in S1 Data.
(PDF)

S8 Fig The phosphorylation status of CLK protein by western blot of extracts from heads of WT, *Clk*-uORF-KO mutant and *Clk*^*Jrk*^ mutant. **(A** and **C)** Fly heads from female **(A)** and male **(C)** of WT and mutant were collected at the indicated times (ZT), and *Clk*^*Jrk*^ was collected at ZT20. Here, *Clk*^*Jrk*^ served as a negative control to validate the specificity of the CLK antibody. Hyper-phosphorylated CLK (hyper phos-CLK) and hypo-phosphorylated CLK (hypo phos-CLK) are indicated on the left, respectively. The nonspecific (N.S.) band is also labeled with an arrow on the right. ACTIN 57B served as the loading control. **(B** and **D)** Quantification of hyper- and hypo-phosphorylated CLK abundance relative to total CLK from **(A** and **C)**, with the average intensity for WT hyper phos-CLK normalized to 1. Underlying data for this figure can be found in S1 Data.
(PDF)

S9 Fig *Clk* uORFs suppress CLK protein translation in fly heads independent of light and circadian clock. **(A–D)** Representative Western blots and quantification of CLK protein on the first day of constant dark for males **(A, B)** and females **(C, D)**, sampled at indicated circadian time (CT) at 4-hr intervals. The average intensity for WT was normalized to 1. **(E–H)** Representative Western blots and quantification of CLK protein in male **(E, F)** and female **(G, H)** *per*^*01*^; *tim*^*01*^ flies, sampled at indicated Zeitgeber time at 4-hr intervals. The average intensity for *per*^*01*^; *tim*^*01*^ was normalized to 1. The analysis included four replicates. Data are presented as mean ± SEM (*n* = 4; Two-tailed Mann–Whitney *U* test. *p < 0.05). Underlying data for this figure can be found in S1 Data.
(PDF)

S10 Fig Genes related to ribosomal proteins and translational factors exhibit rhythmic expression at the protein level. Hierarchical clustering of translation-related proteins (GO: 0006412) expressed rhythmically at the protein level under DD condition in our previous proteomic data. Underlying data for this figure can be found in S1 Data.
(PDF)

S11 Fig Model simulating the effect of knocking out *Clk* uORF on circadian rhythm. **(A)** Diagram of the *Drosophila* circadian clock molecular network, adapted from a previous study. mRNA molecules are indicated in italicized lowercase, while protein molecules are in uppercase. Red arrows represent activation, while blue lines indicate suppression. The bold red arrow from *Clk* mRNA to CLK protein signifies the enhanced translation activity due to *Clk* uORF knockout. The green arrow with an “X” indicates that CRY protein promotes the degradation of TIM. **(B and C)** The simulated relative protein **(B)** and mRNA **(C)** abundance levels of CLK/*Clk*, PER/*per*, and TIM/*tim* along simulation time (hr) upon *Clk*-uORF-KO **(A)**. The time point of increasing regulatory weight from *Clk* mRNA to CLK protein is marked by the red arrow labeled with *Clk*-uORF-KO. **(D)** The distribution of period length (hr) under different *Clk* mRNA to CLK protein regulatory weights across approximately 400 simulation cycles. The original regulatory weight setting of 0.026 in the original model acts as the baseline, emulating the presence of WT uORF. After 200 cycles the regulatory weight is increased from 0.0026 to several arbitrary higher levels (0.027, 0.028, and 0.029) which is denoted by the red arrow, and continued for another 200 simulation cycles. **(E)** The statistics of circadian period length (hr) for simulations in **(D)**. Data are measured in 200 simulation cycles and are reported as the mean ± SEM (Two-tailed Student *t* test. ***p < 0.001). Underlying data for this figure can be found in S1 Data.
(PDF)

S12 Fig Knocking out *Clk* uORF does not substantially alter sleep in males. **(A)** Sleep duration during daytime (L), nighttime (D), and the entire day (LD) of female *Clk-*uORF-KO and WT flies. It matches with Fig 5A and 5B. **(B)** Sleep profile of male *Clk-*uORF-KO and WT flies under LD conditions. **(C)** Sleep duration during daytime (L), nighttime (D), and the entire day (LD) of male *Clk-*uORF-KO and WT flies under. **(D)** Daily sleep bout number, average bout length, and waking activity of male *Clk-*uORF-KO and WT flies under LD condition. Sleep is monitored using the DAMS in **(A–D)** which is an infrared based detection system. **(E)** Sleep profile of female *Clk-*uORF-KO and WT flies monitored using video system under LD conditions. **(F)** Daily sleep duration of female *Clk-*uORF-KO and WT flies under LD monitored using video system. Data are presented as the mean ± SEM. The numbers of flies tested are shown in the brackets of legend or the bottom of histograms. Asterisks indicate statistical significance (Two-tailed Student *t* test. *p < 0.05; ***p < 0.001; n.s., *p *> 0.05). Underlying data for this figure can be found in S1 Data.
(PDF)

S13 Fig Knocking out *Clk* uORFs does not alter the adaptation of locomotor rhythm to seasonal photoperiod changes. Locomotor activity profiles of female WT and *Clk*-uORF-KO female flies under 4-hr L:20-hr D (4L20D), 8-hr L:16-hr D (8L16D), 12-hr L:12-hr D (12L12D) and 20-hr L:4-hr D (20L4D) conditions. The numbers of flies tested (*n*) are shown at the top-right of plots. Data are presented as the mean ± SEM. Underlying data for this figure can be found in S1 Data.
(PDF)

S14 Fig Photoperiod-dependent expression of translational regulation genes. qRT-PCR measured mRNA levels of indicated translation related genes in total RNA extracts from female WT fly heads, sampled at specified Zeitgeber time points under 4L20D or 12L12D. The average intensity of WT under 12L12D is normalized to 1. Data are reported as the mean ± SEM (*n* = 6; Two-tailed Mann–Whitney *U* test. *p < 0.05; **p < 0.01). Underlying data for this figure can be found in S1 Data.
(PDF)

S15 Fig AUC analysis of daily CLK protein level under different photoperiods. AUC analysis of daily CLK protein level of female WT (left) and *Clk-*uORF-KO (right) flies under different photoperiods shown in Fig 7F. Data are presented as the mean ± SEM. Asterisks indicate statistical significance (*n* = 4; Two-tailed Mann–Whitney *U* test for unpaired comparisons. *p < 0.05; n.s., *p *> 0.05). Underlying data for this figure can be found in S1 Data.
(PDF)

S16 Fig Photoperiodic regulation of *Clk* mRNA level. The relative *Clk* mRNA level measured by RT-qPCR under different photoperiod length at each time point. The average value of WT under 12L12D is set to 1. Data are presented as the mean ± SEM (*n* = 4; Two-tailed Mann–Whitney *U* test for unpaired comparisons. *p < 0.05). Underlying data for this figure can be found in S1 Data.
(PDF)

S17 Fig RNA-Seq measured mRNA levels of *per* and *tim* from total RNA extracts of male and female heads, sampled at specified Zeitgeber time points (ZT). Data are reported as the mean ± SEM (*n* = 3; Wald test; ***p < 0.001; n.s., *p *> 0.05). Underlying data for this figure can be found in S1 Data.
(PDF)

S18 Fig *Clk* uORFs modulate the temporal pattern of transcriptome expression landscape in male fly heads. (A) The ratio of sleep-related genes among differentially expressed genes (DEGs) and non-DEGs at each time point in male WT and *Clk-*uORF-KO. Fisher’s exact tests were applied for comparisons at each ZT. n.s., *p *> 0.05; *p < 0.05; ***p < 0.001. **(B)** Heatmap showing the GO terms enriched in male DEGs for each ZT. The heatmap cells were colored by their *p*-values, while gray cells indicate lack of enrichment for that term in the corresponding gene sets. **(C)** The number of cycling genes from whole-head RNA-seq data of male WT and *Clk*-uORF-KO flies were determined by MetaCycle under two *q*-value cutoffs (JTK and ARS, FDR [*q* value] ≤ 0.05 or 0.01). The percentage of cycling genes out of a total of 9,202 expressed genes is indicated at the bottom of each bar. Fisher’s exact tests; n.s., *p *> 0.05**. (D)** The number of cycling genes shared between male WT and *Clk*-uORF-KO under two *q*-value cutoffs (*q* value ≤ 0.05, or 0.01). The percentage is also indicated below the gene number. Underlying data for this figure can be found in S1 Data.
(PDF)

S19 Fig *Clk* uORFs modulate the rhythmic expression of circadian rhythm-related genes. **(A)** The ratio of circadian rhythm-related genes among cycling genes and non-cycling genes in females and male WT and *Clk*-uORF-KO. The number of circadian rhythm-related genes and cycling genes or non-cycling genes are denoted at the bottom of each bar. Fisher’s exact tests were applied for comparisons at each ZT. *p < 0.05; **p < 0.01; ***p < 0.001. **(B)** The distribution of relative amplitudes (rAMP, log_10_ transformed) for cycling genes in the heads of WT and KO flies (*n* = 289, *p *= 4.74 × 10^−15^ for females; *n* = 230, *p *= 1.21 × 10^−5^ for males; Wilcoxon signed-rank test). Underlying data for this figure can be found in S1 Data.
(PDF)

S20 Fig *Clk* uORFs modulate the temporal transcriptomic profile in a sex-dependent manner. **(A)** The overlap of cycling genes in WT female and WT male. **(B)** The overlap of cycling genes in *Clk*-uORF-KO female and *Clk*-uORF-KO male. **(C)** The overlap of genes that gain rhythmic expression in female and male *Clk*-uORF-KO. **(D)** The overlap of genes that loss rhythmic expression in female and male *Clk*-uORF-KO. **(E)** Heatmap showing the GO terms enriched among genes that gained/lost rhythmic expression in females and males. The heatmap cells were colored by their *p*-values, while gray cells indicate lack of enrichment for that term in the corresponding gene sets.
(PDF)

S21 Fig Knocking out *Clk* uORFs reduces starvation resistance. Survival curves of female and male flies under starvation condition (*n* = 200; log-rank test; ***p < 0.001). Underlying data for this figure can be found in S1 Data.
(PDF)

S22 Fig Knocking out *Clk* uORFs impairs fecundity. **(A)** The average 1-day egg number (left) and summed 8-day number (right) laid by a mated female of *Clk-*uORF-KO and WT over 8 consecutive days. Data are presented as the mean ± SEM (*n *= 50; Wilcoxon rank-sum test; **p < 0.01; n.s., *p *> 0.05). **(B)** The average 2-day egg number (left) and summed 10-day number (right) laid by a virgin of *Clk-*uORF-KO and WT over 10 consecutive days. Data are presented as the mean ± SEM (*n *= 50; Wilcoxon rank-sum test; *p < 0.05; ***p < 0.001; n.s., *p *> 0.05). **(C)** The offspring number per female parent of *Clk-*uORF-KO and WT over 10 days at 25°C (left) and 29°C (right) (*n *= 20; Wilcoxon rank-sum test; ***p < 0.001). Underlying data for this figure can be found in S1 Data.
(PDF)

S23 Fig Differential expression of *cyc* and *Clk* in *Drosophila* heads, with *cyc* showing higher abundance across sequencing methods. In female heads, *Clk* exhibits RPKM values of 12.73 in mRNA-Seq and 2.82 in Ribo-Seq, while *cyc* has values of 22.72 and 16.12, respectively. In male heads, *Clk*’s RPKM values are 12.83 in mRNA-Seq and 2.70 in Ribo-Seq, compared to *cyc*’s 26.35 and 6.41, respectively. Analysis was based on our previously published transcriptome and translatome data [39].
(PDF)

S24 Fig The circadian period and sleep of *Clk*-uORF-KO mutants on the isogenic *w*^*1118*^ background. **(A)** Period length of locomotor rhythms of *Clk*-uORF-KO flies and *w*^*1118*^ flies under DD. The period lengths (hr) and the number of flies tested (*n*) are displayed above and below each bar, respectively. **(B)** Sleep duration over two consecutive days of female *Clk*-uORF-KO and *w*^*1118*^ flies under LD. The sleep duration (min) of each day and the number of flies tested (*n*) are displayed above and below each bar, respectively. **(C)** Sleep profile of female *Clk*-uORF-KO and *w*^*1118*^ flies under LD conditions. Data are presented as the mean ± SEM. Asterisks indicate statistical significance (Two-tailed Student *t* test. ***p < 0.001). Underlying data for this figure can be found in S1 Data.
(PDF)

S25 Fig Sleep profile of heterozygous and homozygous *Clk-uORF* KO *versus w^1118^* flies. **(A)** Sleep profile of female *w*^*1118*^, heterozygous and homozygous *Clk*-uORF-KO under LD conditions. **(B)** Sleep duration over two consecutive days of indicated flies in **(A)**. The sleep duration (min) of each day and the number of flies tested (*n*) are displayed above and below each bar, respectively. Data are presented as the mean ± SEM. Asterisks indicate statistical significance (Two-tailed Student *t* test. ***p < 0.001). Underlying data for this figure can be found in S1 Data.
(PDF)

S26 Fig Knocking out *Clk* uORFs does not alter PDF neuron morphology. **(A and B)** Representative example of s-LNvs (1, 2, 3 and 4) and l-LNvs (1′, 2′, 3′ and 4′) soma **(A)** and the morphology of PDF projections **(B)** in female WT and *Clk*-uORF-KO. The scale bars are represented respectively. **(C)** Sholl analysis of the complexity of the s-LNvs axonal arbor stained by PDF antibody. Concentric circles separated by 5 μm were centered at the point of intersection, where the projections of the s-LNvs intersect. **(D)** Quantification of the axonal cross with concentric circles in **(C)**. Data are reported as the mean ± SEM (*n* = 6; Two-tailed Mann–Whitney *U* test for unpaired comparisons. n.s., *p *> 0.05). Underlying data for this figure can be found in S1 Data.
(PDF)

S27 Fig The distribution and conservation of uORFs in circadian rhythm-related genes in human. **(A)** The distribution of uORF number in circadian rhythm-related genes and other genes in humans. The gene number (*n*) in each class are denoted at the bottom (Wilcoxon rank-sum test, *p *= 0.033). **(B)** ECDF of the BLSs for uATGs in core clock genes, non-core clock genes and other genes, respectively. The gene number in each class is denoted in the parentheses (Wilcoxon rank-sum test, *p* = 1.92 × 10^−5^ for non-core clock genes versus other genes; *p* = 0.08 for core clock gene versus non-core clock genes; *p* = 0.00046 for core clock gene versus other genes. n.s., *p *> 0.05; *p < 0.05; ***p < 0.001). Underlying data for this figure can be found in S1 Data.
(PDF)

S1 Text Supplemental materials and methods. (DOCX)

S1 Table The putative canonical uORFs in *D. melanogaster.* (XLSX)

S2 Table The uORFs in the circadian rhythm-related genes (GO:0007623). (XLSX)

S3 Table Highly conserved uORFs across 23 *Drosophila* species. (XLSX)

S4 Table The median, mean, and standard error of period length under different regulatory weight. (XLSX)

S5 Table Free-running locomotor rhythm of *Clk*-uORF-KO flies. (XLSX)

S6 Table Sleep-related genes. (CSV)

S7 Table Expression rhythmicity inferred from RNA-seq data by MetaCycle. (XLSX)

S8 Table Key resources table. (XLSX)

S1 Data Collection of numerical source data for Figs 1A, 1B, 1C, 2C, 3A, 3B, 3E, 3F, 4A, 4B, 4C, 5A, 5B, 5C, 5D, 5E, 5F, 6A, 6B, 6C, 7A, 7B, 7C, 7D, 7F, 8A, 8C, 8D, S1C, S2A, S2B, S4A, S4B, S5A, S5B, S6A, S6B, S7B, S7D, S8D, S9B, S9D, S9F, S9H, S10, S11D, S11E, S12A, S12B, S12C, S12D, S12E, S12F, S13, S14, S15, S16, S17, S18A, S18C, S18D, S19A, S19B, S21, S22A, S22B, S22C, S24A, S24B, S24C, S25A, S25B, S26D, S27A and S27B. (XLSX)

S1 Raw Images Uncropped blots for immunoblotting data. (PDF)

## Abbreviations

**:**
BLS: branch length score
CDS: coding sequence
DAMS: *Drosophila* activity monitoring system
DEGs: differentially expressed genes
KO: knockout
MSA: multiple sequence alignment
PAM: protocerebral anterior medial
PDF: pigment dispersing factor
PTM: post-translational modification
P-to-M: polysome-to-monosome
RPF: ribosome-protected footprints
SAD: seasonal affective disorder
TOC1: timing of CAB expression 1
TTFL: transcription–translation feedback loop
uORFs: upstream open reading frames;
WRST: Wilcoxon rank-sum test;
ZT,: Zeitgeber time.
