# Supplementary figures and images for: Upstream open reading frames dynamically modulate CLOCK protein translation to regulate circadian rhythms and sleep
Source: PLoS Biol. 2025 May 12;23(5):e3003173. doi: 10.1371/journal.pbio.3003173 (PMC12121920; doi:10.1371/journal.pbio.3003173)

A

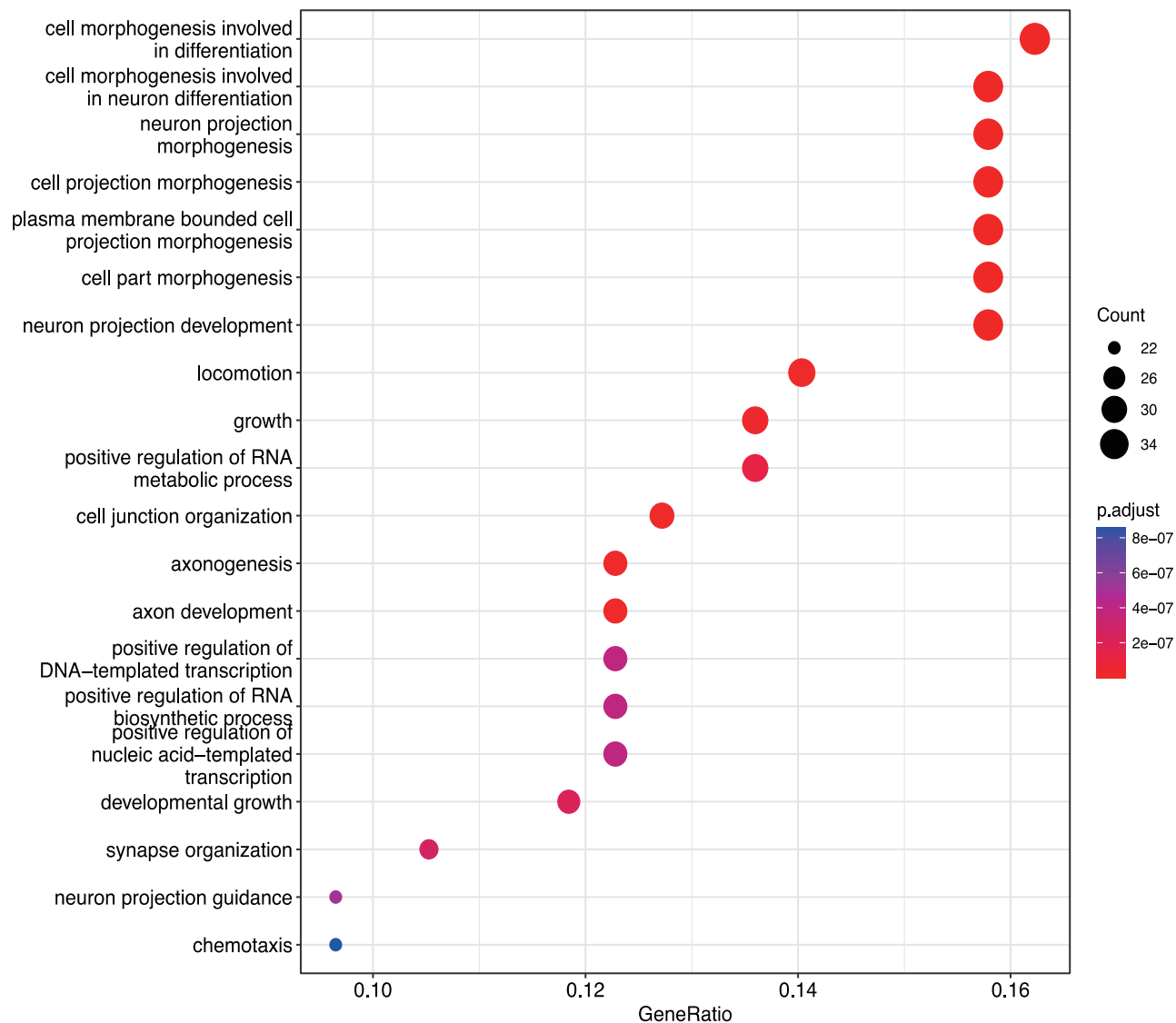

B

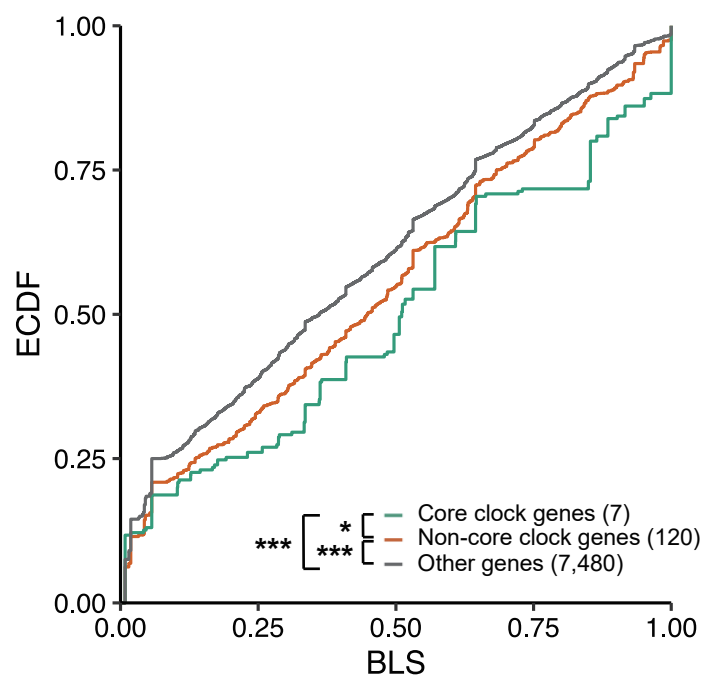

C

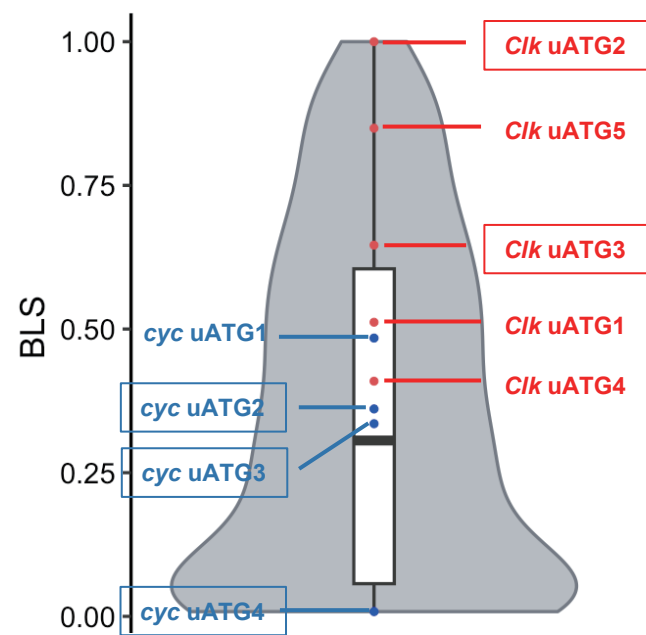

Supplement: S1 Fig — (A) GO-based enrichment analysis for genes containing uORFs conserved across 23 Drosophila species. (B) Empirical cumulative distribution function (ECDF) of the BLSs for uATGs in core clock genes, non-core clock genes and other non-clock genes, respectively. The ECDF curve represents the cumulative probability distribution of BLS values. A curve shifted to the left indicates smaller BLS values (lower conservation). The gene number in each class is denoted in parentheses. Wilcoxon rank-sum test; *p < 0.05; ***p < 0.001. (C) The BLS distribution of all uORFs, Clk uORFs and cyc uORFs. The five uORFs of Clk are marked as red dots, with the corresponding numbers labeled on the right side. The four uORFs of cyc are marked as blue dots, with the corresponding numbers labeled on the left side. The knocked-out uATGs are in boxes. Underlying data for this figure can be found in S1 Data. (PDF) [file pbio.3003173.s001.pdf]

**A**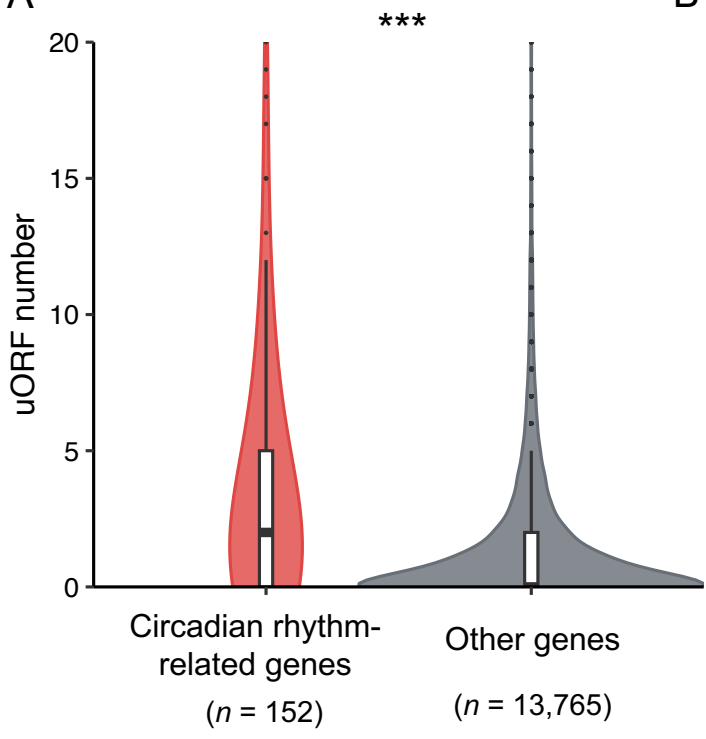**B**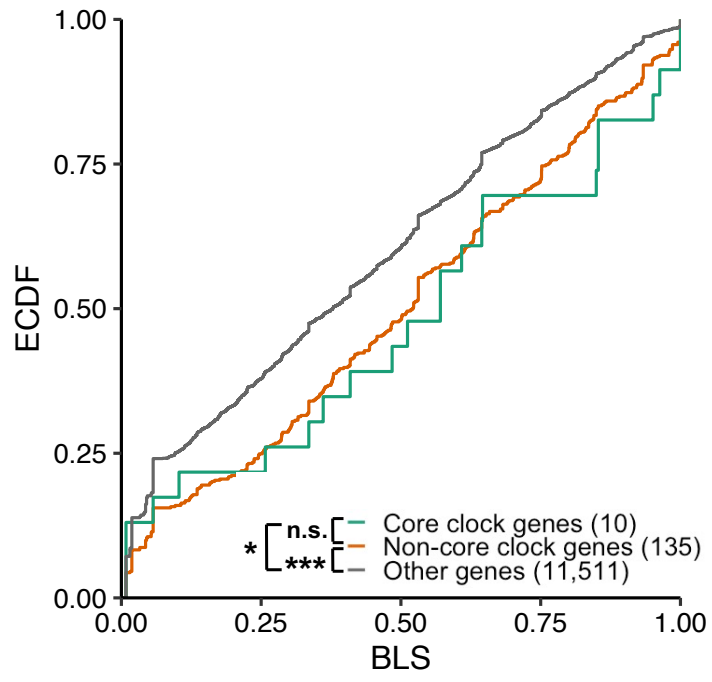

Supplement: S2 Fig — (A) The distribution of uORF number in circadian rhythm-related genes and other genes in Drosophila. The gene number (n) in each class are denoted at the bottom. (B) ECDF of the BLSs for uATGs in core circadian clock genes, non-core clock genes and other genes, respectively. The gene number in each class is denoted in the parentheses. Notably, only the most abundant isoform for each gene is considered in the analysis. Wilcoxon rank-sum test; n.s., p > 0.05; *p < 0.05; ***p < 0.001. Underlying data for this figure can be found in S1 Data. (PDF) [file pbio.3003173.s002.pdf]

A

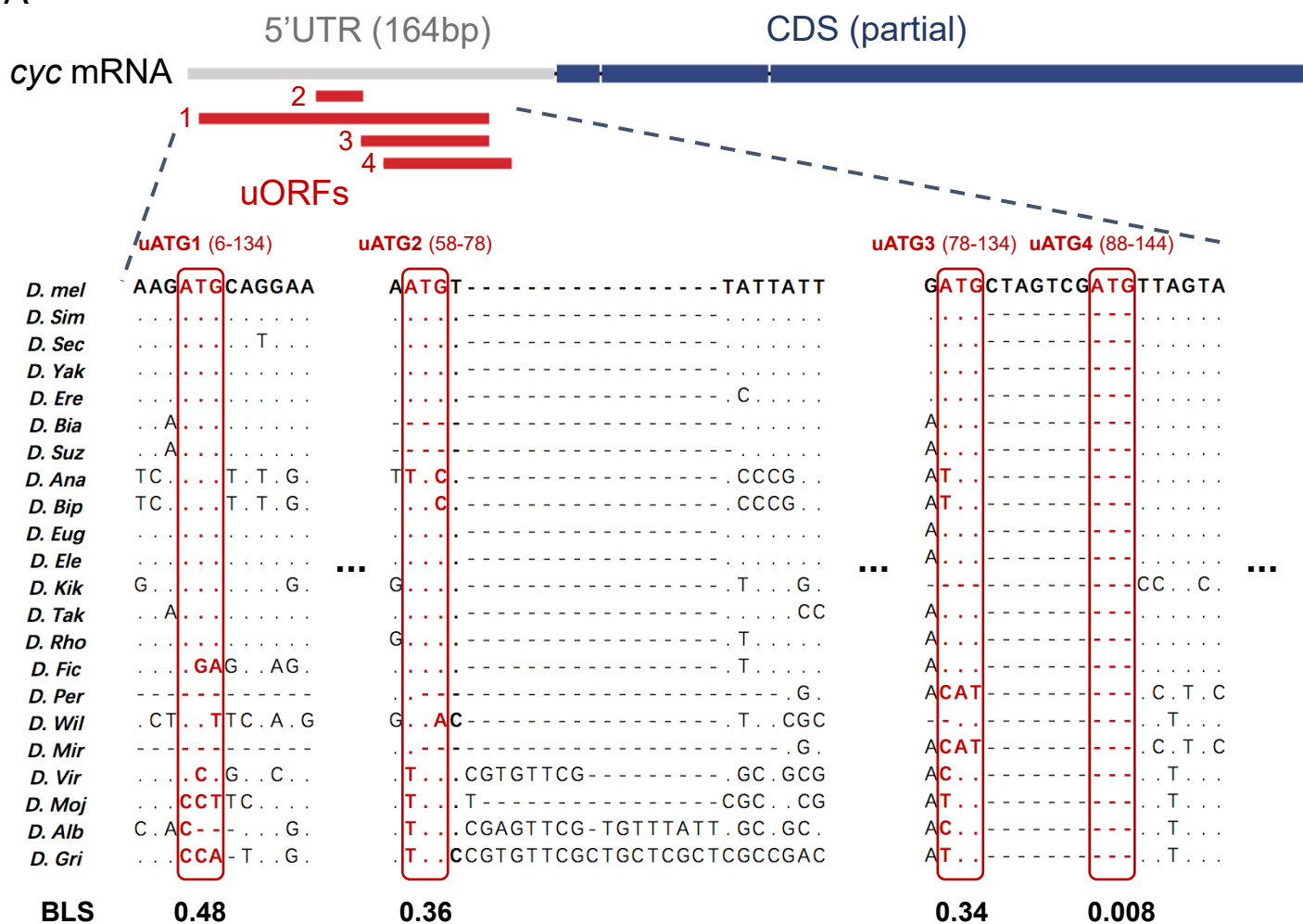

B

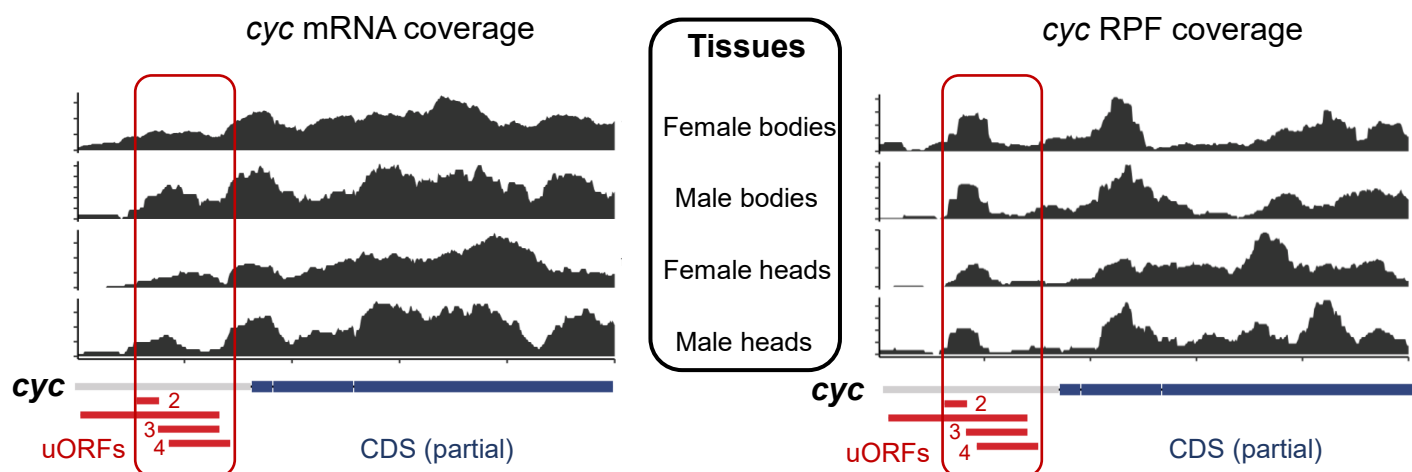

Supplement: S3 Fig — (A) MSA of cyc uORFs among 23 Drosophila species. The start codons (uATGs) of uORFs are highlighted by red boxes. The position schemes of uORFs and partial CDSs are denoted above the MSA, with red and blue colors respectively. The start and ending positions of each uORF (separated by “hyphen”) are given in the parenthesis above each uATG. The BLS of each uATG were denoted below MSA. (B) The mRNA reads coverage (left) and ribosome-protected footprints (RPF) coverage (right) of uORFs of cyc mRNA from the heads and bodies. The position schemes of uORFs and partial CDSs are denoted at the bottom. Underlying data for this figure can be found in S1 Data. (PDF) [file pbio.3003173.s003.pdf]

A

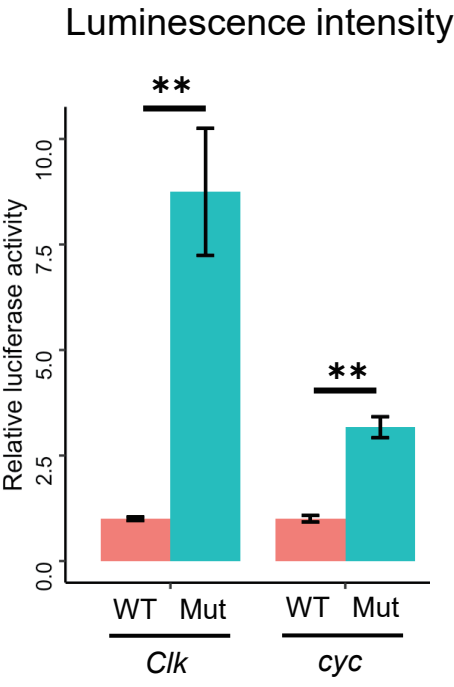

B

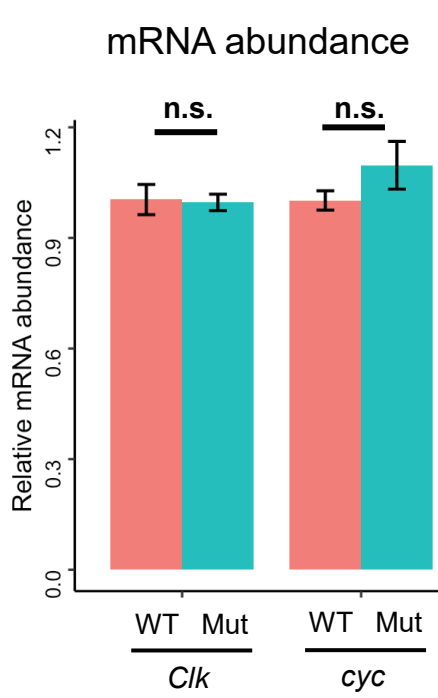

Supplement: S4 Fig — (A and B) The luminescence intensity (A) and mRNA abundance (B) under the control of 5' UTR containing mutant uORFs (Mut) and WT 5' UTR of Clk or cyc, respectively. The relative mRNA abundance is measured by RT-qPCR. Data represent mean ± SEM (n = 6). In (A), p = 0.0036 for Clk and p = 0.0022 for cyc. In (B), p = 0.87 for Clk and p = 0.22 for cyc. Two-tailed Student t test; n.s., p > 0.05; **p < 0.01. Underlying data for this figure can be found in S1 Data. (PDF) [file pbio.3003173.s004.pdf]

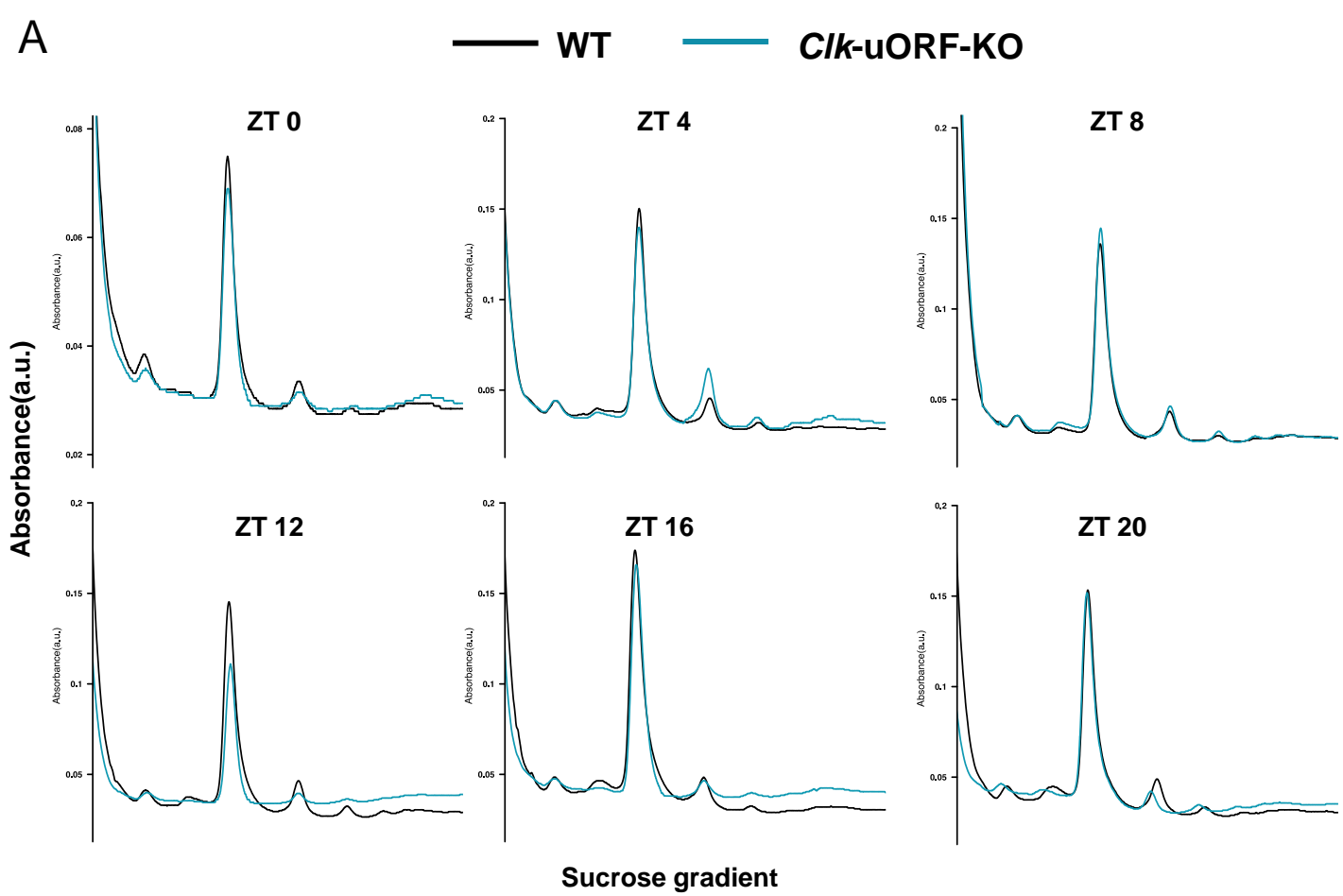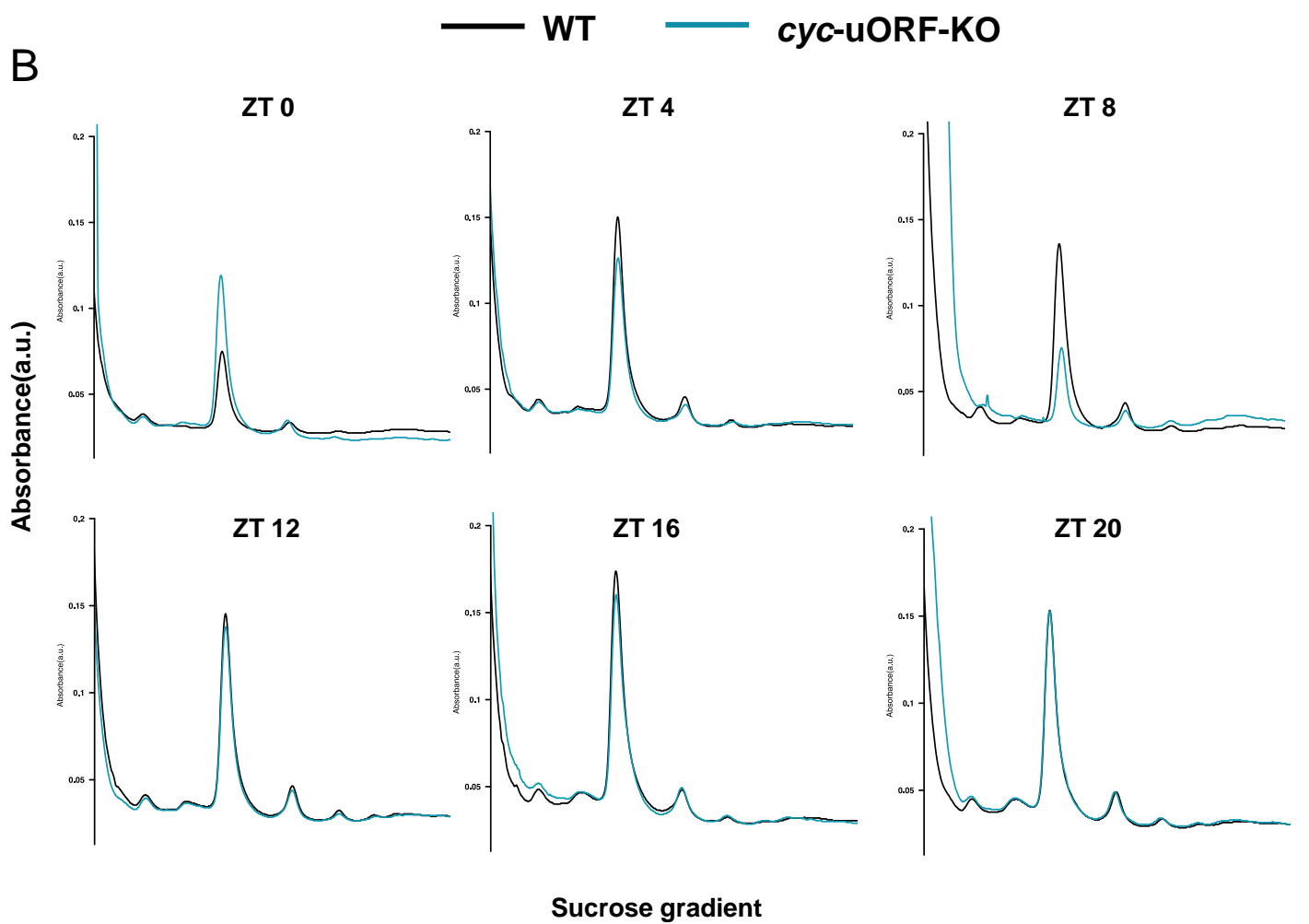

Supplement: S6 Fig — (A and B) Ribosome profiles of fly heads from WT, Clk-uORF-KO flies (A) and cyc-uORF-KO flies (B) at the indicated time points, measured by sucrose gradient. Underlying data for this figure can be found in S1 Data. (PDF) [file pbio.3003173.s006.pdf]

A

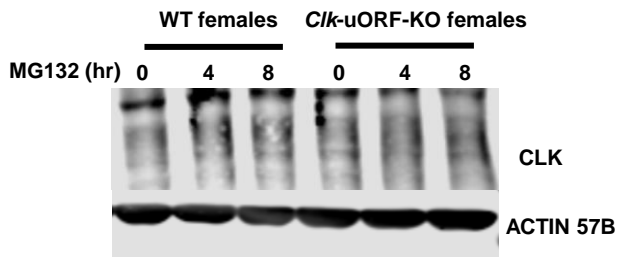

B

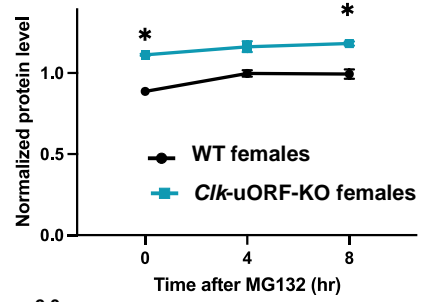

C

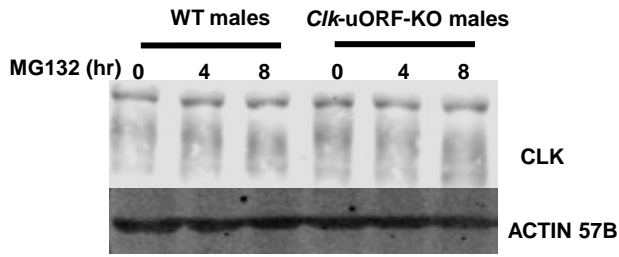

D

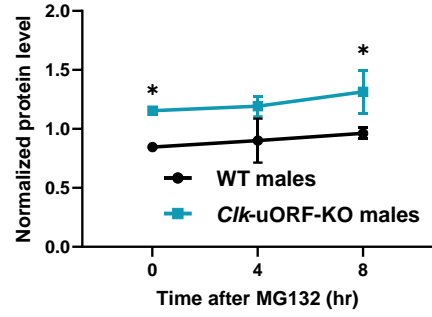

E

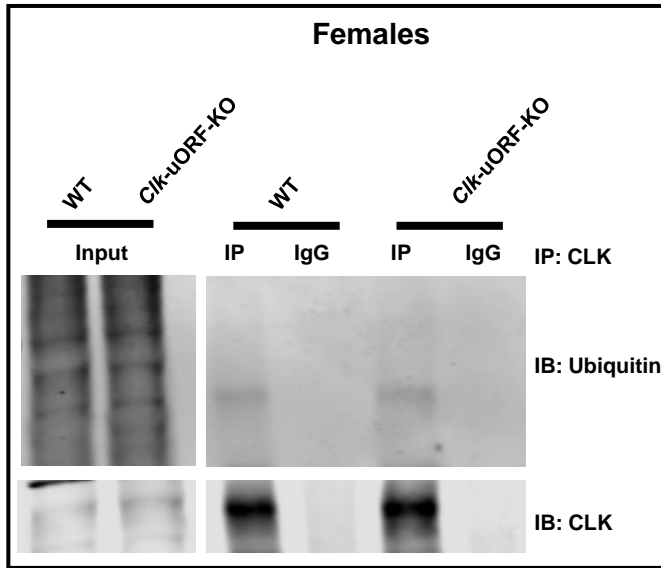

F

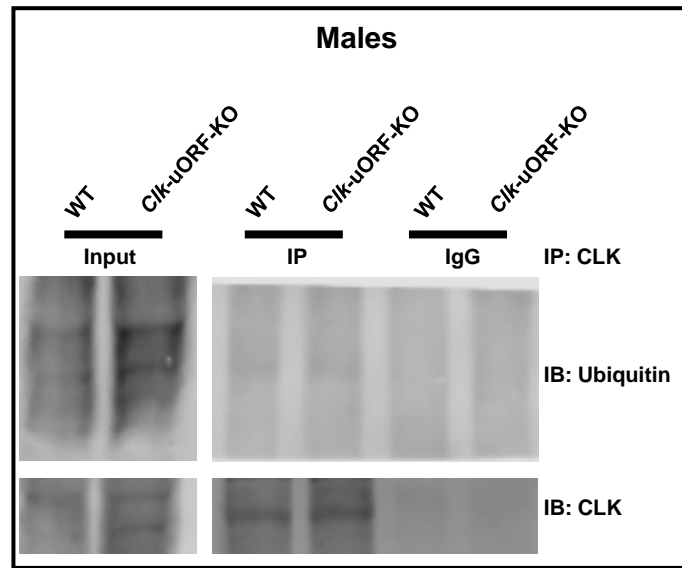

Supplement: S7 Fig — (A and C) Representative Western blots of CLK protein from whole-head protein extracts of female and male flies fed with proteasome inhibitor MG132 (200 µM) for indicated hours prior to collection. ACTIN 57B served as the loading control. (B and D) Quantification of CLK abundance normalized to ACTIN 57B from (A and C), with the average intensity for WT and Clk-uORF-KO normalized to 1 at 0 hr. Data are presented as mean ± SEM (n = 4; Two-tailed Mann–Whitney U test. *p < 0.05). (E and F) Immunoprecipitation assays were performed using protein extracts from female (E) and male (F) fly heads with anti-CLK antibody or IgG control. Precipitates were blotted with the indicated antibodies. IP, immunoprecipitation; IB, immunoblotting. Underlying data for this figure can be found in S1 Data. (PDF) [file pbio.3003173.s007.pdf]

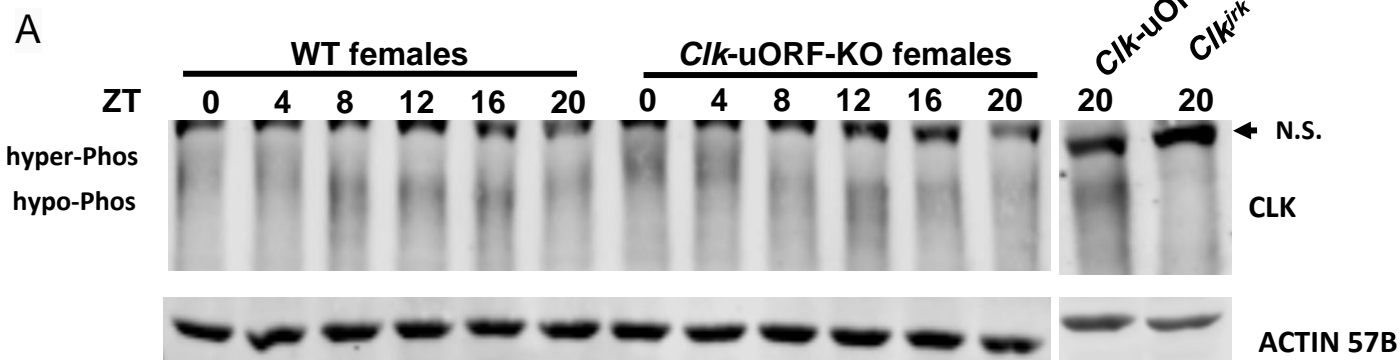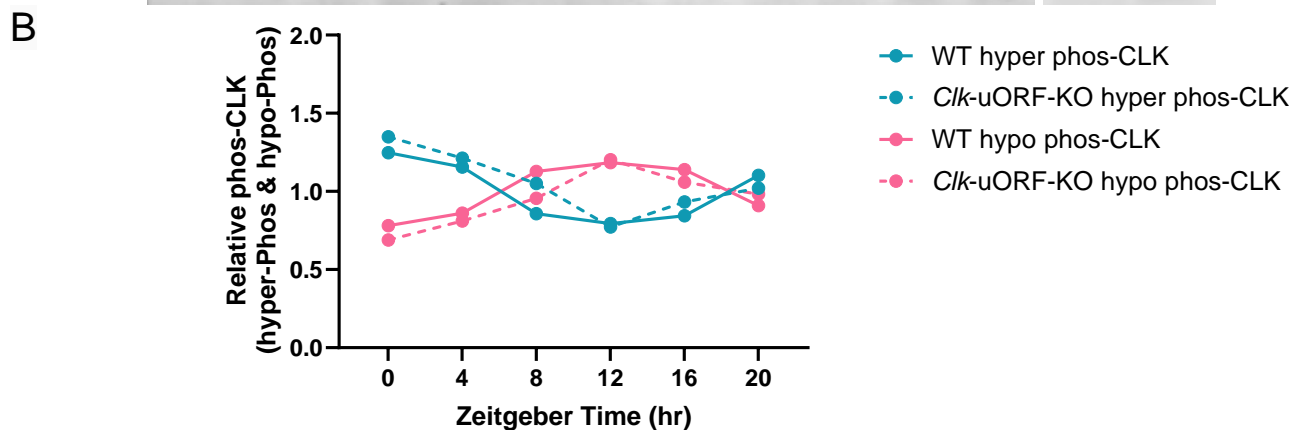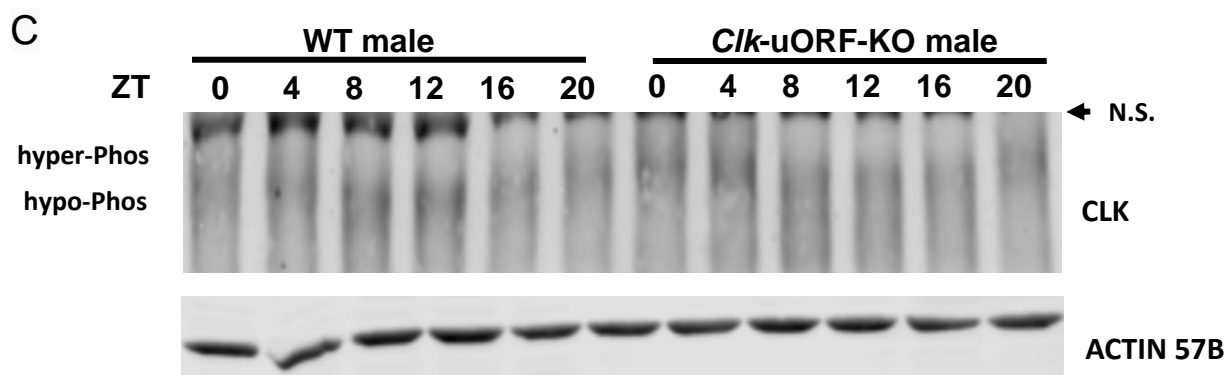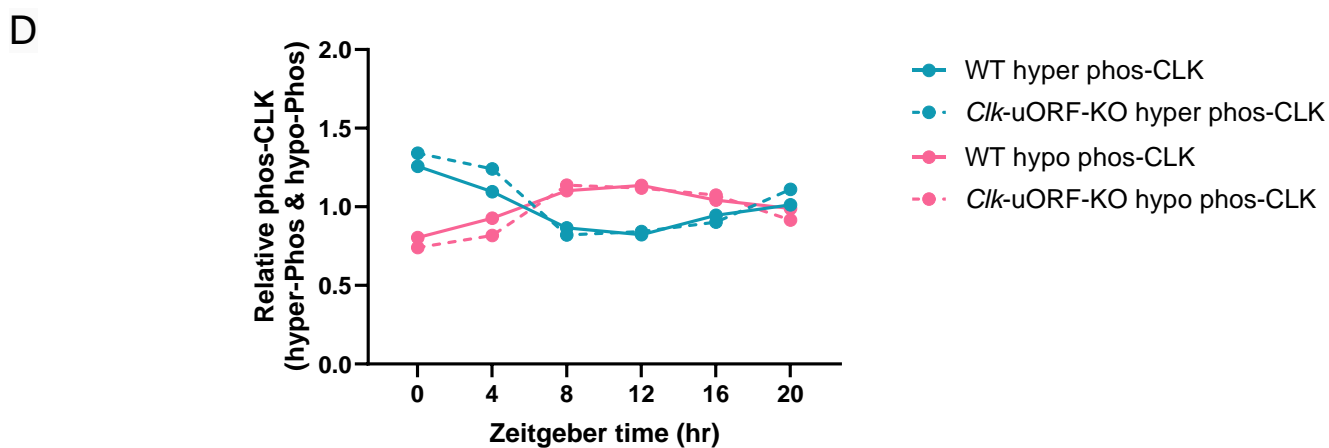

Supplement: S8 Fig — (A and C) Fly heads from female (A) and male (C) of WT and mutant were collected at the indicated times (ZT), and ClkJrk was collected at ZT20. Here, ClkJrk served as a negative control to validate the specificity of the CLK antibody. Hyper-phosphorylated CLK (hyper phos-CLK) and hypo-phosphorylated CLK (hypo phos-CLK) are indicated on the left, respectively. The nonspecific (N.S.) band is also labeled with an arrow on the right. ACTIN 57B served as the loading control. (B and D) Quantification of hyper- and hypo-phosphorylated CLK abundance relative to total CLK from (A and C), with the average intensity for WT hyper phos-CLK normalized to 1. Underlying data for this figure can be found in S1 Data. (PDF) [file pbio.3003173.s008.pdf]

A

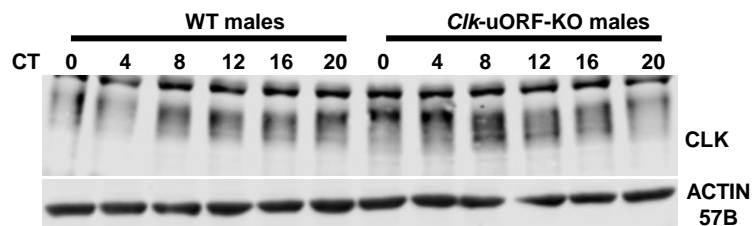

B

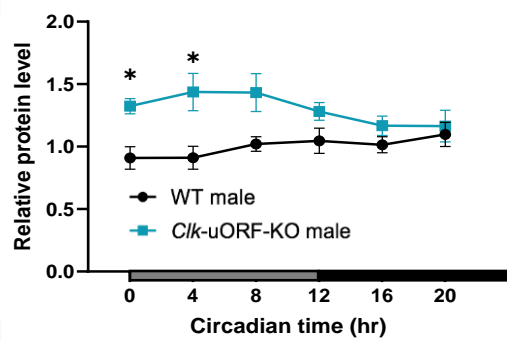

C

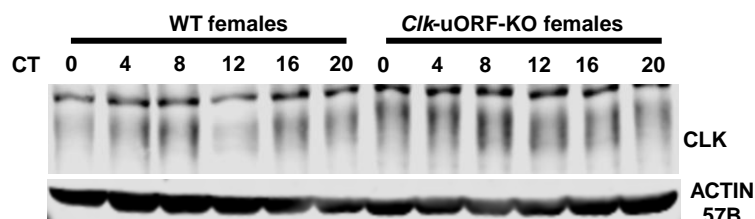

D

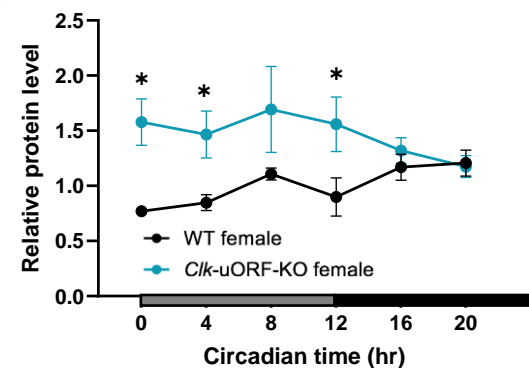

E

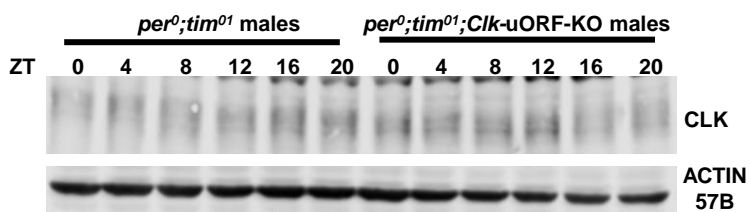

F

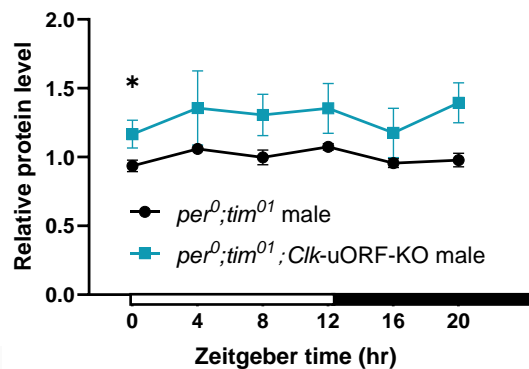

G

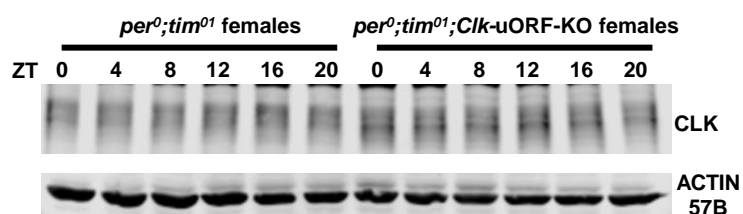

H

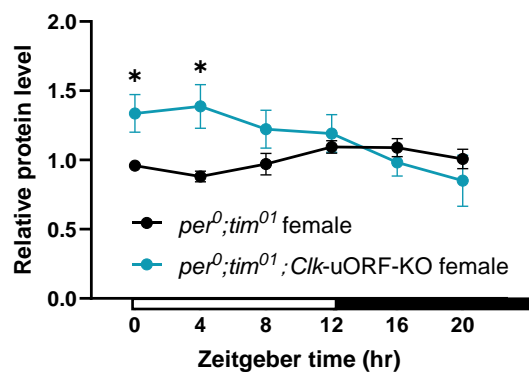

Supplement: S9 Fig — (A–D) Representative Western blots and quantification of CLK protein on the first day of constant dark for males (A, B) and females (C, D), sampled at indicated circadian time (CT) at 4-hr intervals. The average intensity for WT was normalized to 1. (E–H) Representative Western blots and quantification of CLK protein in male (E, F) and female (G, H) per01; tim01 flies, sampled at indicated Zeitgeber time at 4-hr intervals. The average intensity for per01; tim01 was normalized to 1. The analysis included four replicates. Data are presented as mean ± SEM (n = 4; Two-tailed Mann–Whitney U test. *p < 0.05). Underlying data for this figure can be found in S1 Data. (PDF) [file pbio.3003173.s009.pdf]

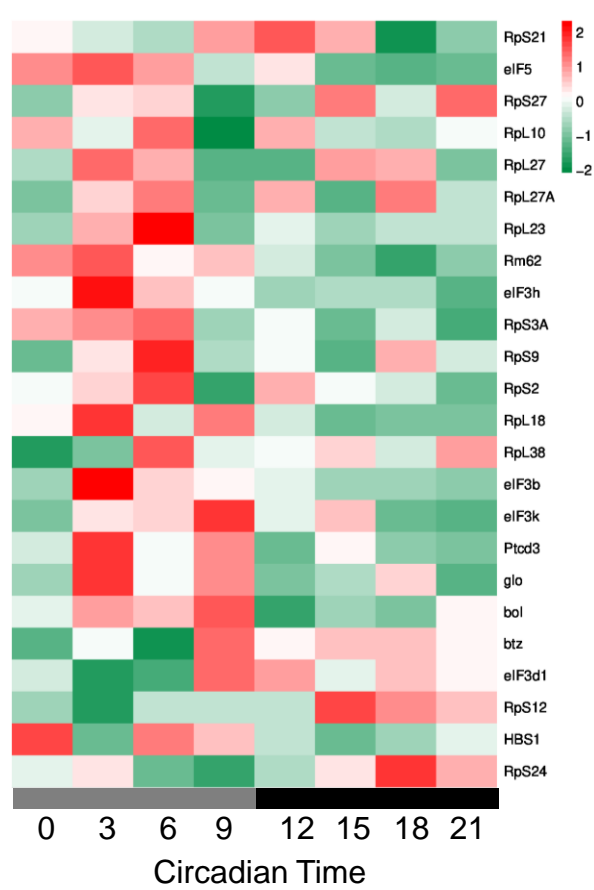

Supplement: S10 Fig — Hierarchical clustering of translation-related proteins (GO: 0006412) expressed rhythmically at the protein level under DD condition in our previous proteomic data. Underlying data for this figure can be found in S1 Data. (PDF) [file pbio.3003173.s010.pdf]

# A

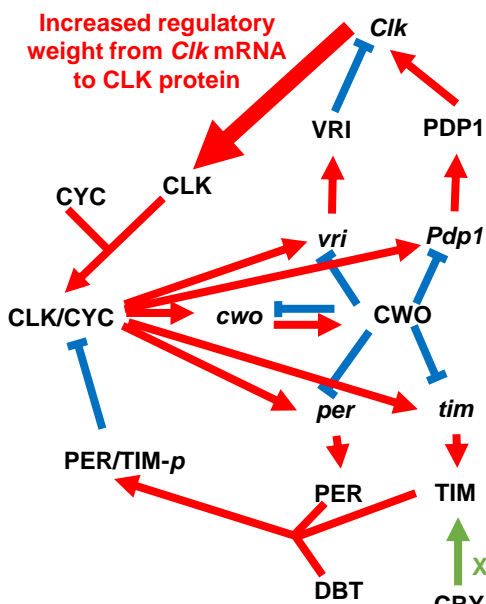

B

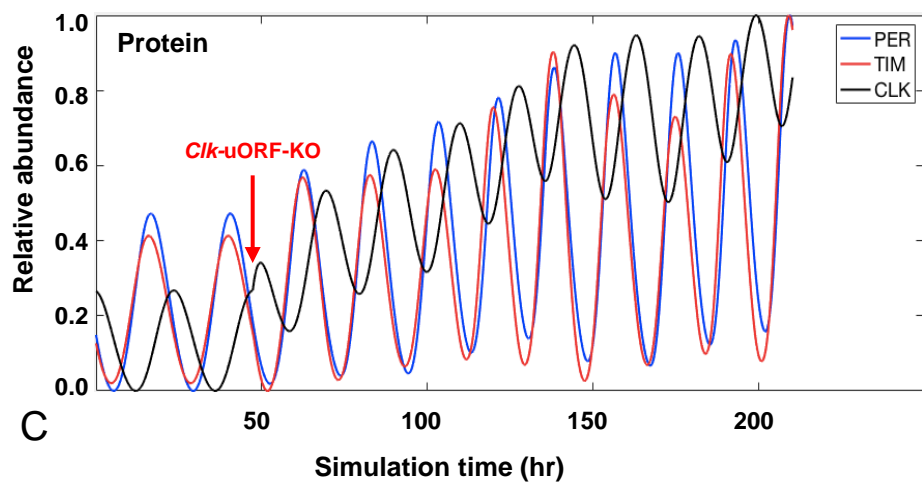

Q

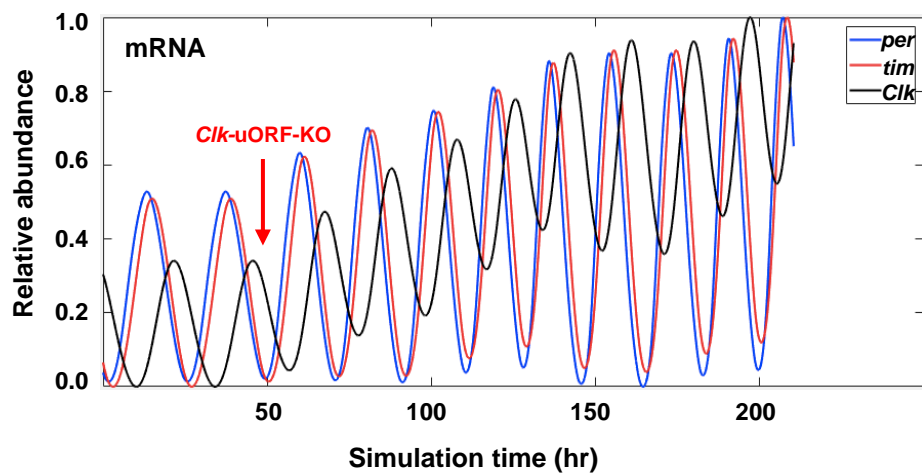

D

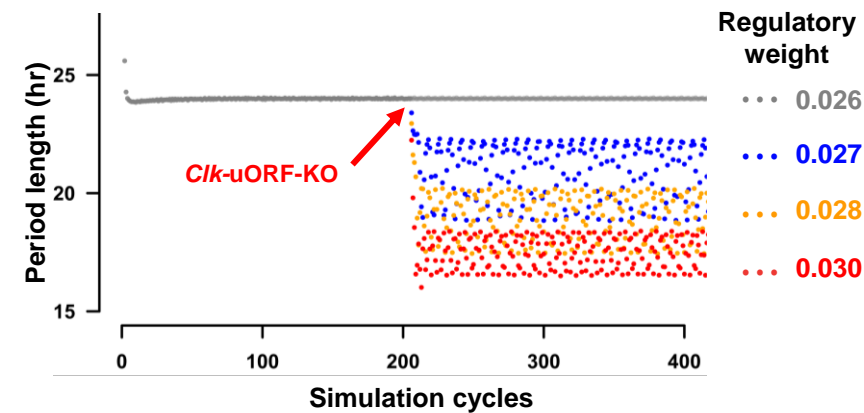

E

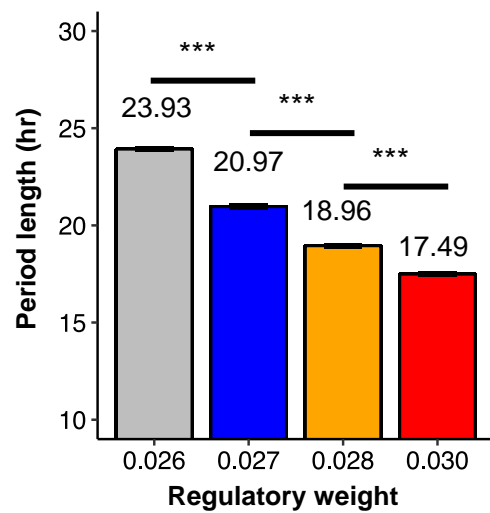

Supplement: S11 Fig — (A) Diagram of the Drosophila circadian clock molecular network, adapted from a previous study. mRNA molecules are indicated in italicized lowercase, while protein molecules are in uppercase. Red arrows represent activation, while blue lines indicate suppression. The bold red arrow from Clk mRNA to CLK protein signifies the enhanced translation activity due to Clk uORF knockout. The green arrow with an “X” indicates that CRY protein promotes the degradation of TIM. (B and C) The simulated relative protein (B) and mRNA (C) abundance levels of CLK/Clk, PER/per, and TIM/tim along simulation time (hr) upon Clk-uORF-KO (A). The time point of increasing regulatory weight from Clk mRNA to CLK protein is marked by the red arrow labeled with Clk-uORF-KO. (D) The distribution of period length (hr) under different Clk mRNA to CLK protein regulatory weights across approximately 400 simulation cycles. The original regulatory weight setting of 0.026 in the original model acts as the baseline, emulating the presence of WT uORF. After 200 cycles the regulatory weight is increased from 0.0026 to several arbitrary higher levels (0.027, 0.028, and 0.029) which is denoted by the red arrow, and continued for another 200 simulation cycles. (E) The statistics of circadian period length (hr) for simulations in (D). Data are measured in 200 simulation cycles and are reported as the mean ± SEM (Two-tailed Student t test. ***p < 0.001). Underlying data for this figure can be found in S1 Data. (PDF) [file pbio.3003173.s011.pdf]

A

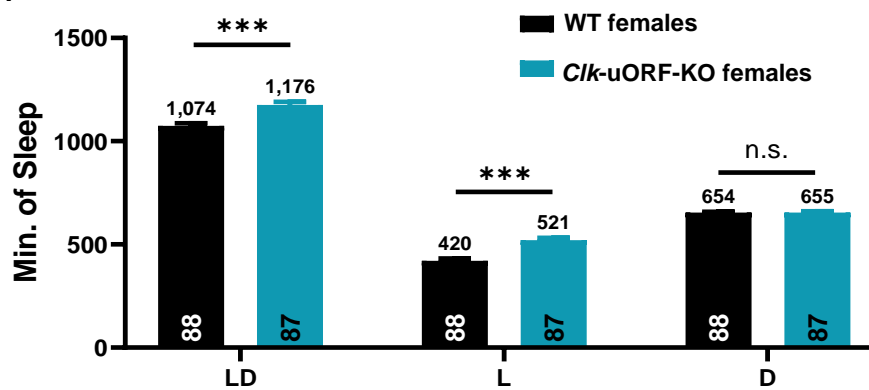

B

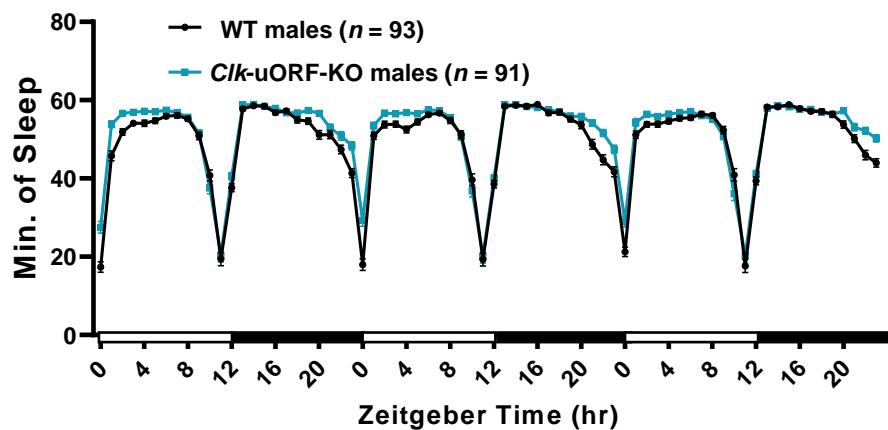

C

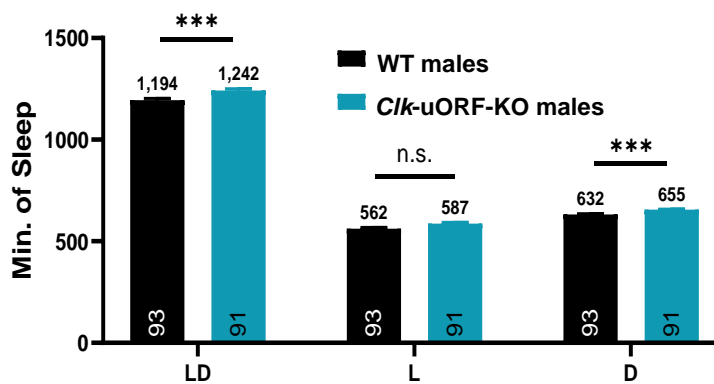

D

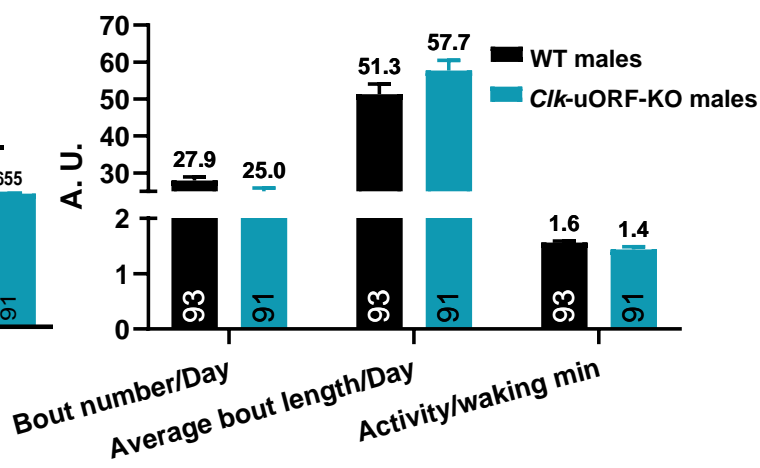

E

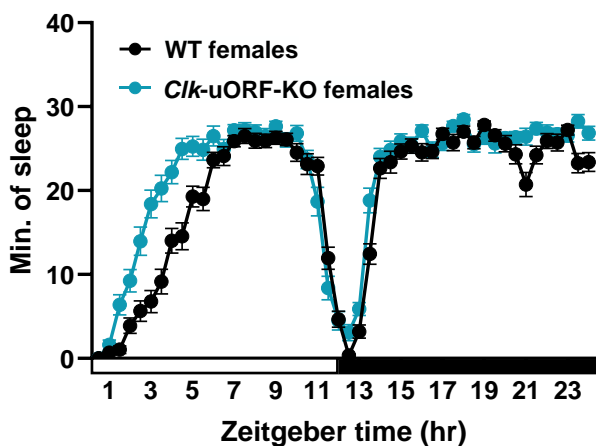

F

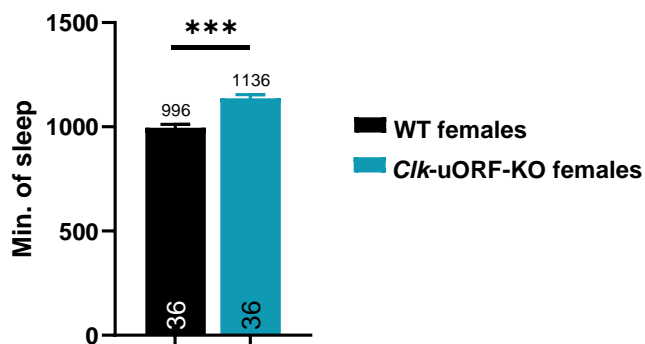

Supplement: S12 Fig — (A) Sleep duration during daytime (L), nighttime (D), and the entire day (LD) of female Clk-uORF-KO and WT flies. It matches with Fig 5A and 5B. (B) Sleep profile of male Clk-uORF-KO and WT flies under LD conditions. (C) Sleep duration during daytime (L), nighttime (D), and the entire day (LD) of male Clk-uORF-KO and WT flies under. (D) Daily sleep bout number, average bout length, and waking activity of male Clk-uORF-KO and WT flies under LD condition. Sleep is monitored using the DAMS in (A–D) which is an infrared based detection system. (E) Sleep profile of female Clk-uORF-KO and WT flies monitored using video system under LD conditions. (F) Daily sleep duration of female Clk-uORF-KO and WT flies under LD monitored using video system. Data are presented as the mean ± SEM. The numbers of flies tested are shown in the brackets of legend or the bottom of histograms. Asterisks indicate statistical significance (Two-tailed Student t test. *p < 0.05; ***p < 0.001; n.s., p > 0.05). Underlying data for this figure can be found in S1 Data. (PDF) [file pbio.3003173.s012.pdf]

● WT females

■ *Clk-uORF-KO* females

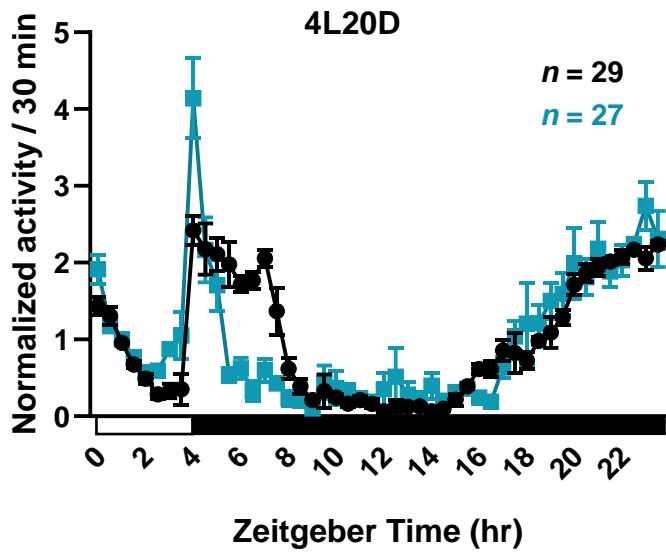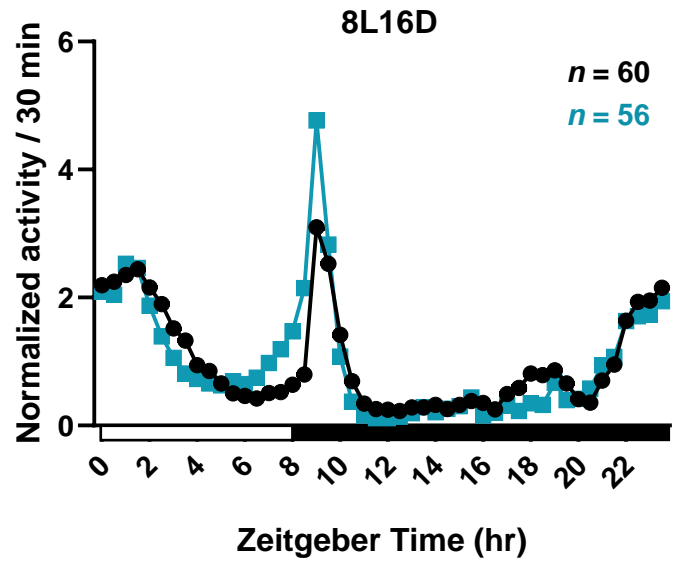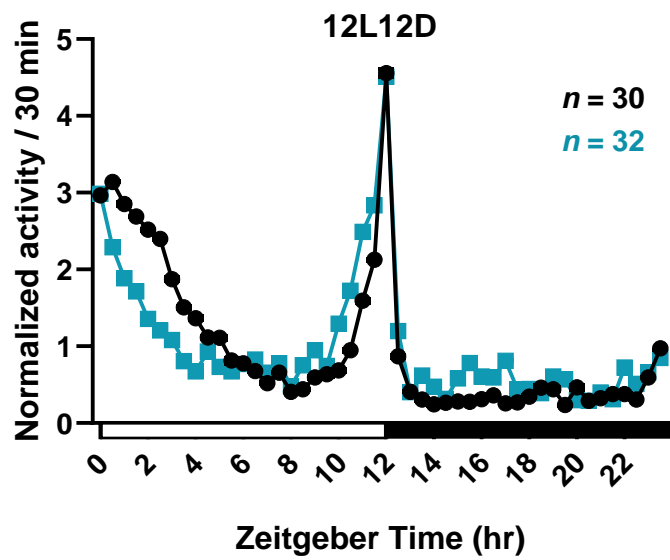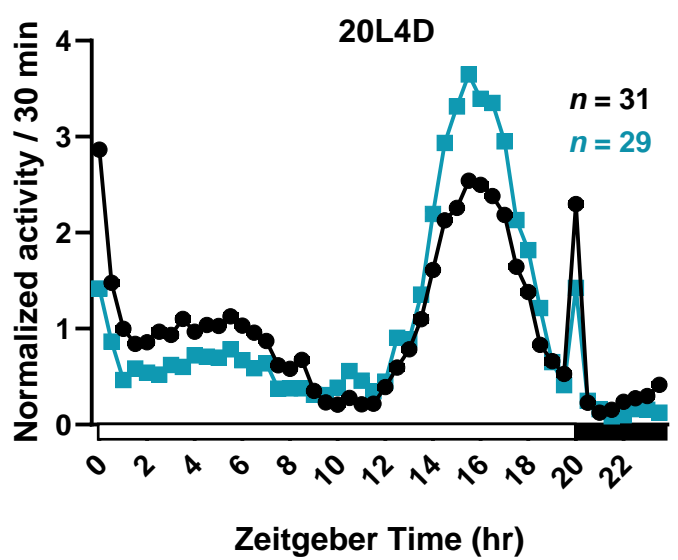

Supplement: S13 Fig — Locomotor activity profiles of female WT and Clk-uORF-KO female flies under 4-hr L:20-hr D (4L20D), 8-hr L:16-hr D (8L16D), 12-hr L:12-hr D (12L12D) and 20-hr L:4-hr D (20L4D) conditions. The numbers of flies tested (n) are shown at the top-right of plots. Data are presented as the mean ± SEM. Underlying data for this figure can be found in S1 Data. (PDF) [file pbio.3003173.s013.pdf]

*RpS4*

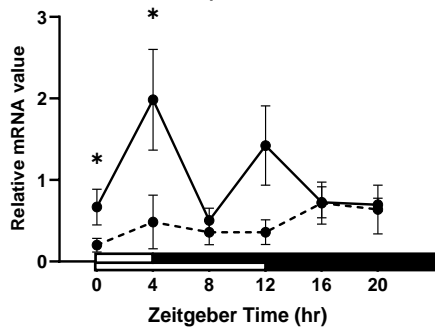

*RpL34a*

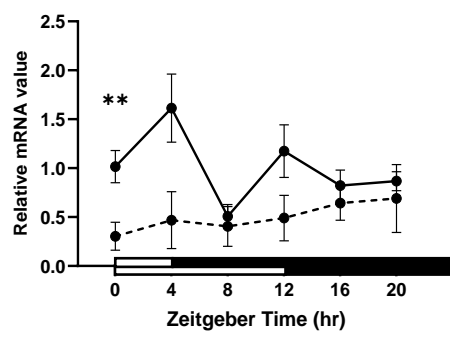

*RpL14*

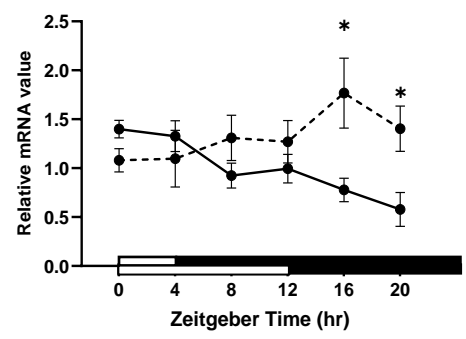

Supplement: S14 Fig — qRT-PCR measured mRNA levels of indicated translation related genes in total RNA extracts from female WT fly heads, sampled at specified Zeitgeber time points under 4L20D or 12L12D. The average intensity of WT under 12L12D is normalized to 1. Data are reported as the mean ± SEM (n = 6; Two-tailed Mann–Whitney U test. *p < 0.05; **p < 0.01). Underlying data for this figure can be found in S1 Data. (PDF) [file pbio.3003173.s014.pdf]

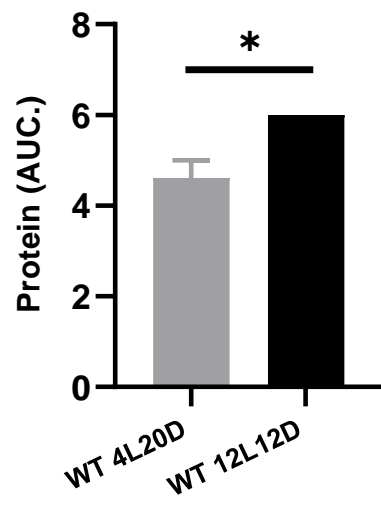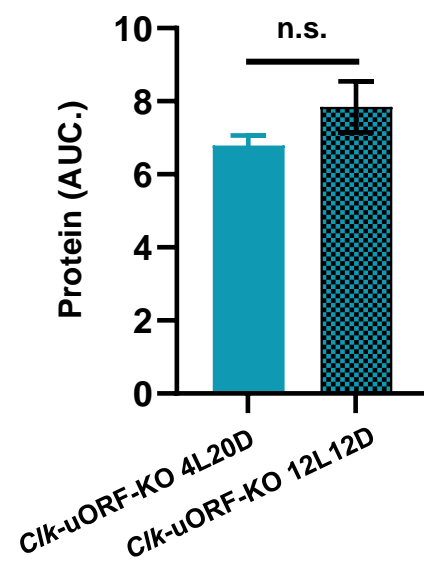

Supplement: S15 Fig — AUC analysis of daily CLK protein level of female WT (left) and Clk-uORF-KO (right) flies under different photoperiods shown in Fig 7F. Data are presented as the mean ± SEM. Asterisks indicate statistical significance (n = 4; Two-tailed Mann–Whitney U test for unpaired comparisons. *p < 0.05; n.s., p > 0.05). Underlying data for this figure can be found in S1 Data. (PDF) [file pbio.3003173.s015.pdf]

WT female

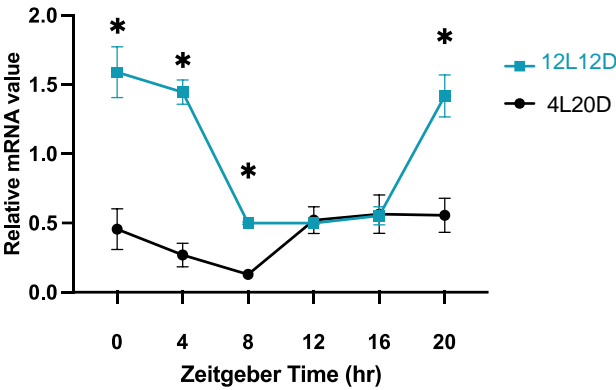

*Clk*-uORF-KO female

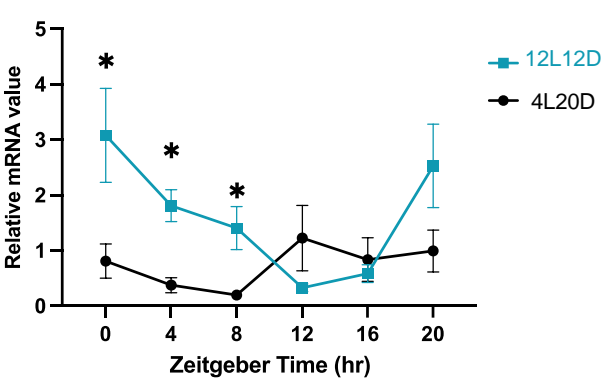

Supplement: S16 Fig — The relative Clk mRNA level measured by RT-qPCR under different photoperiod length at each time point. The average value of WT under 12L12D is set to 1. Data are presented as the mean ± SEM (n = 4; Two-tailed Mann–Whitney U test for unpaired comparisons. *p < 0.05). Underlying data for this figure can be found in S1 Data. (PDF) [file pbio.3003173.s016.pdf]

—●— WT

—■— *Clk-uORF-KO*

### Male heads

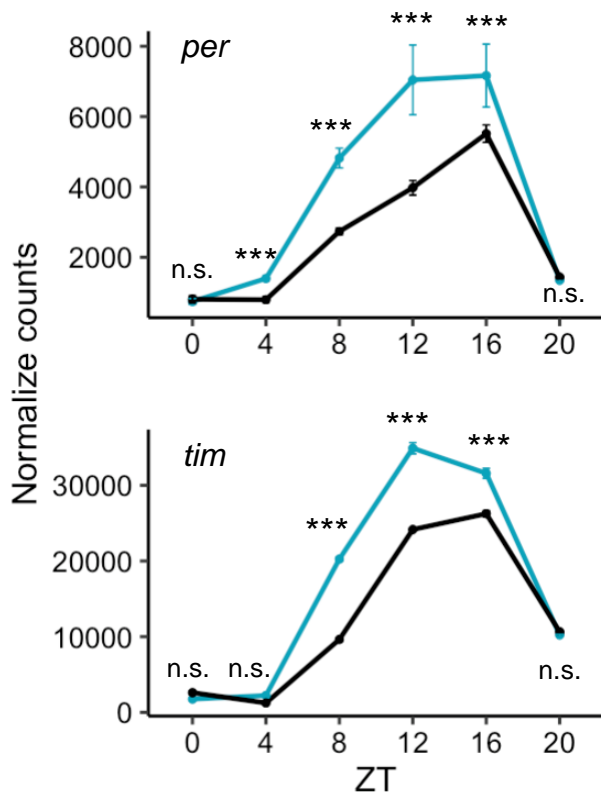

### Female heads

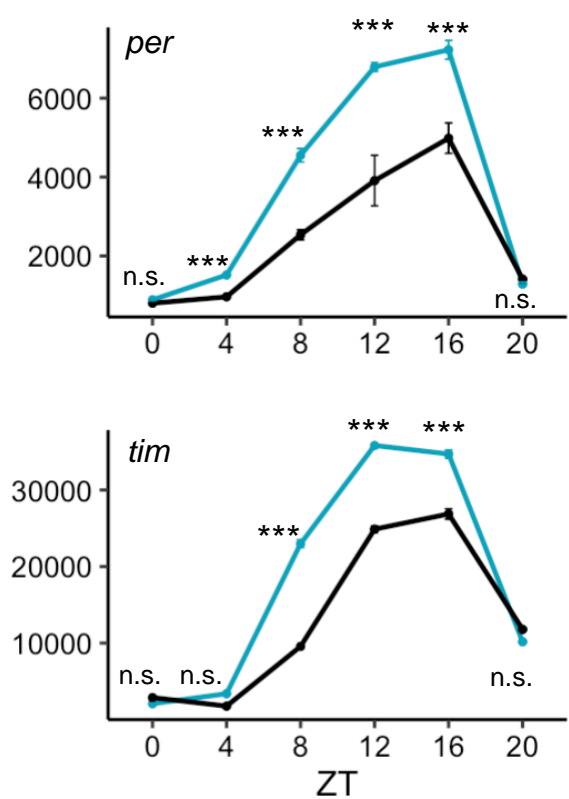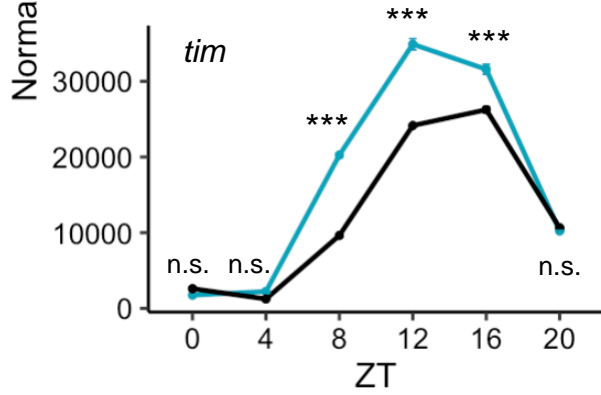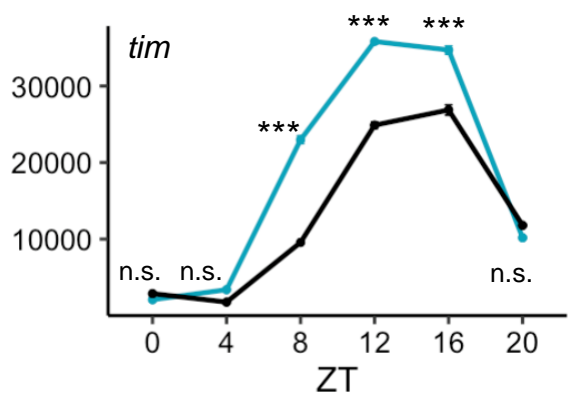

Supplement: S17 Fig — Data are reported as the mean ± SEM (n = 3; Wald test; ***p < 0.001; n.s., p > 0.05). Underlying data for this figure can be found in S1 Data. (PDF) [file pbio.3003173.s017.pdf]

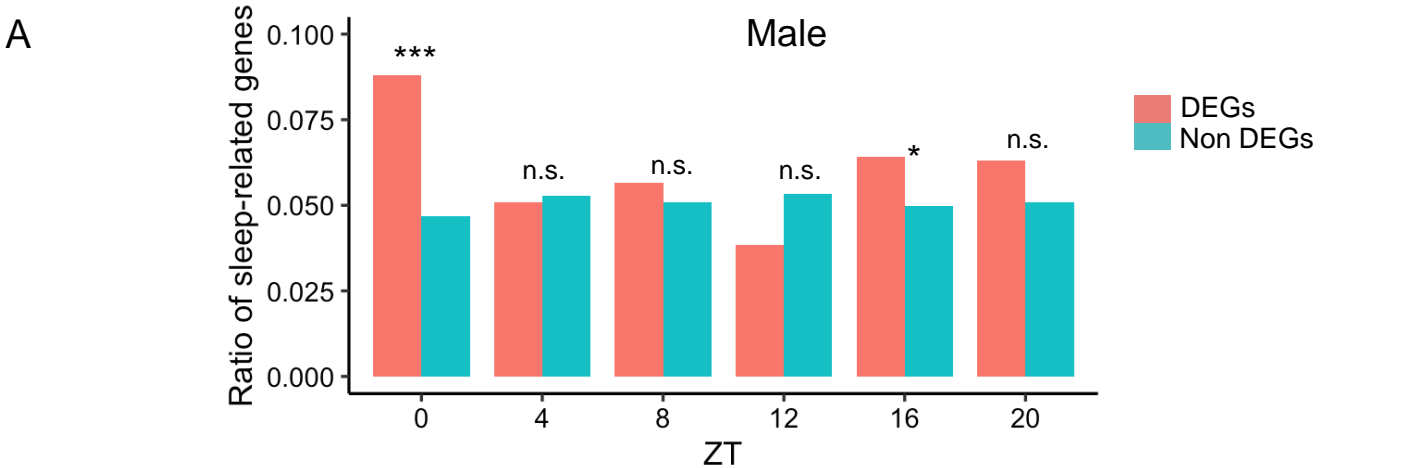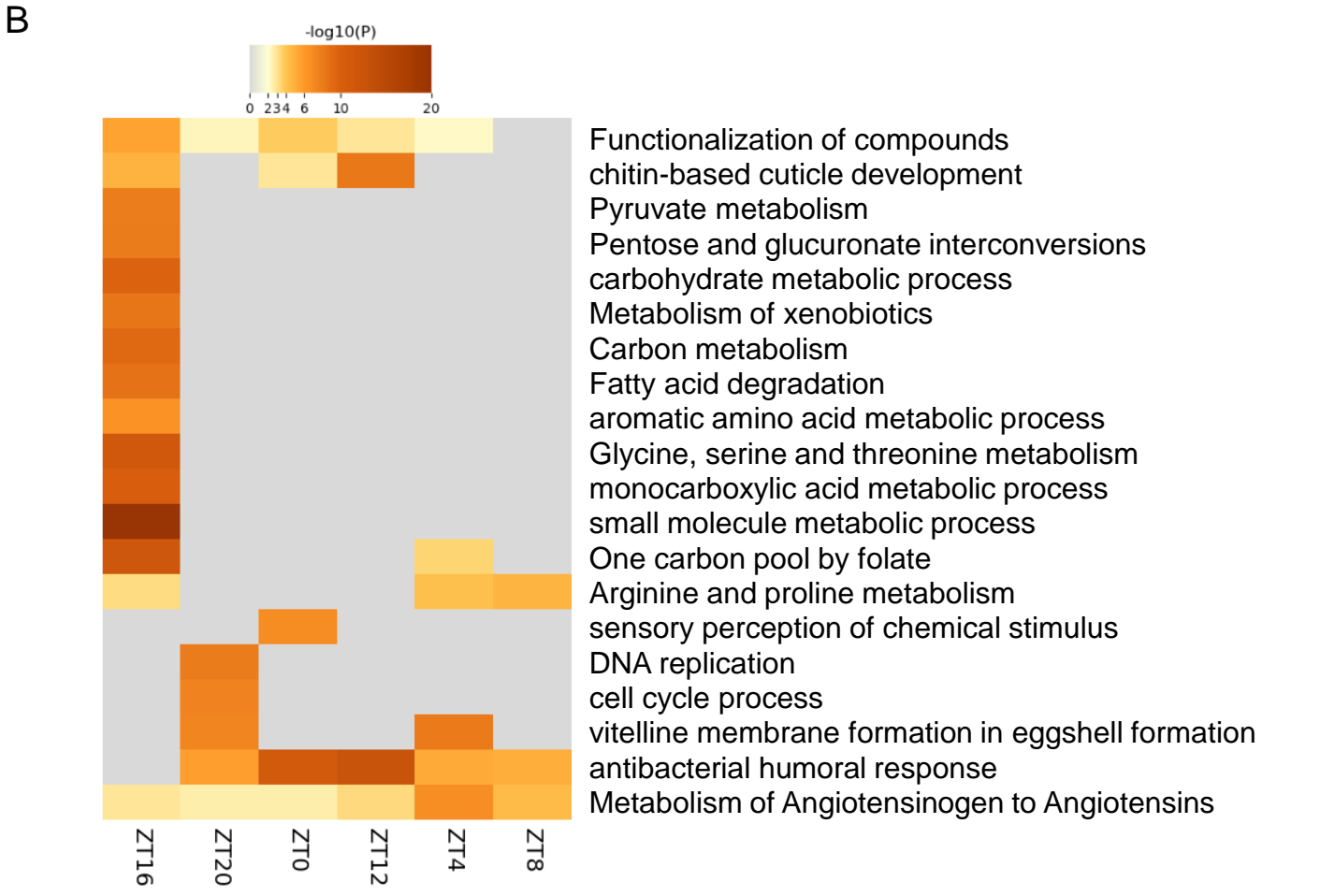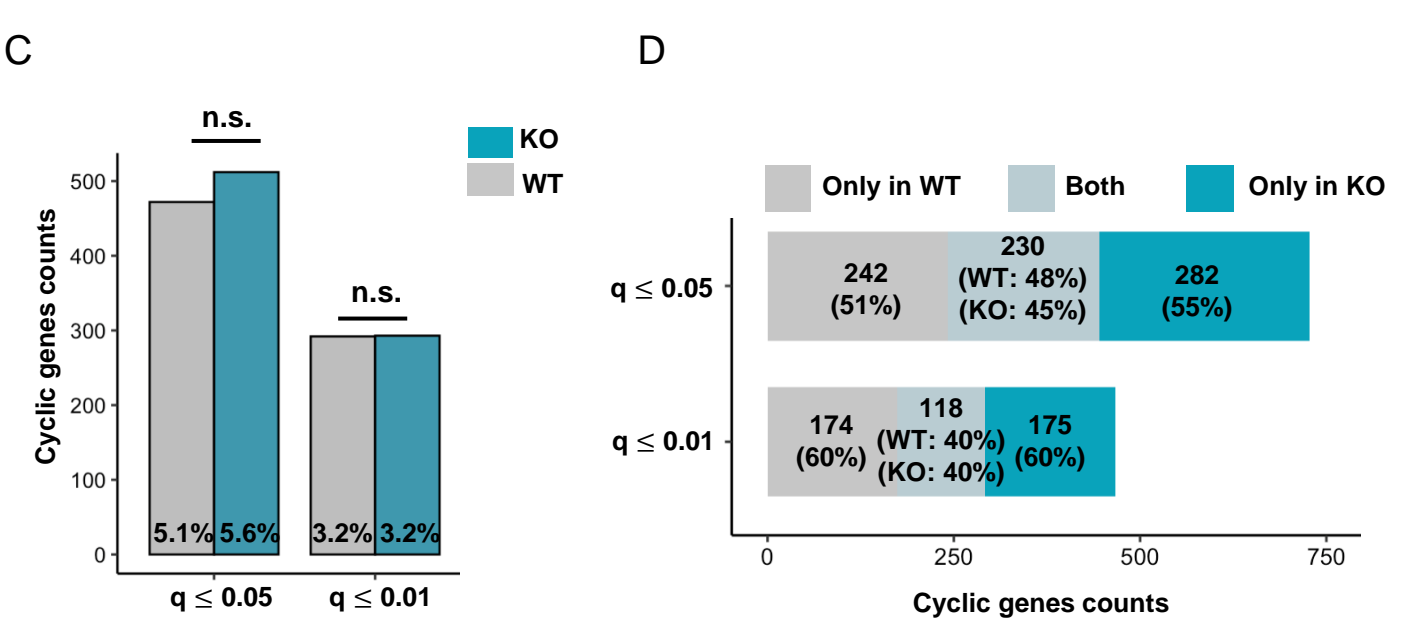

Supplement: S18 Fig — (A) The ratio of sleep-related genes among differentially expressed genes (DEGs) and non-DEGs at each time point in male WT and Clk-uORF-KO. Fisher’s exact tests were applied for comparisons at each ZT. n.s., p > 0.05; *p < 0.05; ***p < 0.001. (B) Heatmap showing the GO terms enriched in male DEGs for each ZT. The heatmap cells were colored by their p-values, while gray cells indicate lack of enrichment for that term in the corresponding gene sets. (C) The number of cycling genes from whole-head RNA-seq data of male WT and Clk-uORF-KO flies were determined by MetaCycle under two q-value cutoffs (JTK and ARS, FDR [q value] ≤ 0.05 or 0.01). The percentage of cycling genes out of a total of 9,202 expressed genes is indicated at the bottom of each bar. Fisher’s exact tests; n.s., p > 0.05. (D) The number of cycling genes shared between male WT and Clk-uORF-KO under two q-value cutoffs (q value ≤ 0.05, or 0.01). The percentage is also indicated below the gene number. Underlying data for this figure can be found in S1 Data. (PDF) [file pbio.3003173.s018.pdf]

A

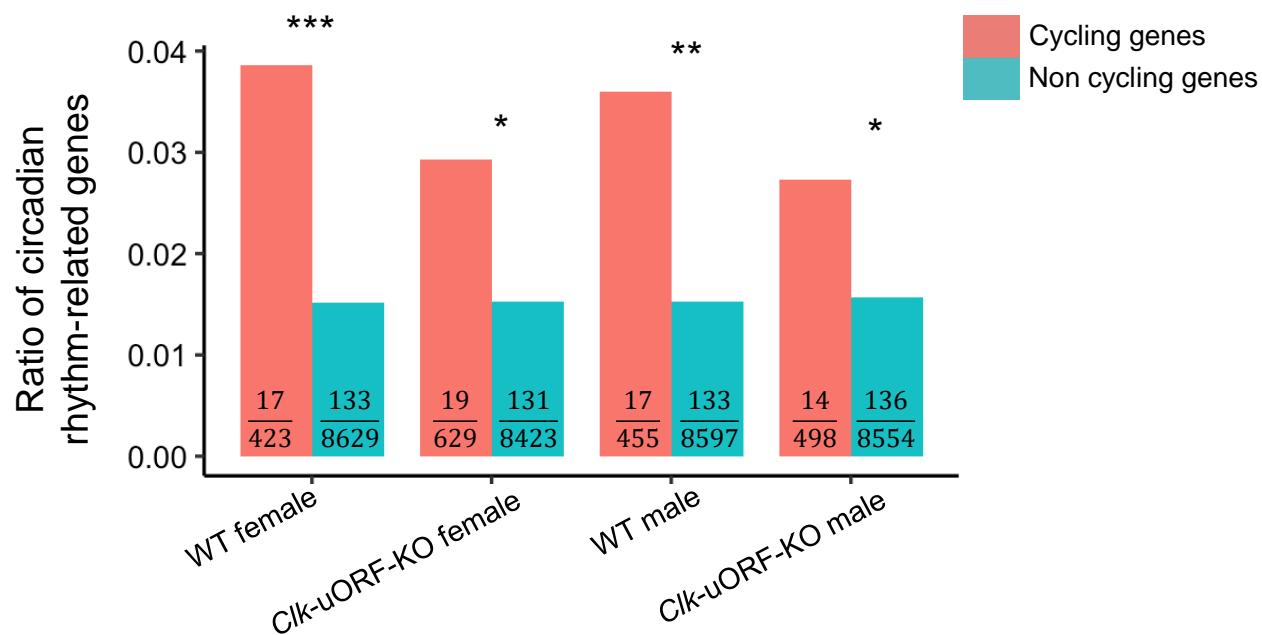

B

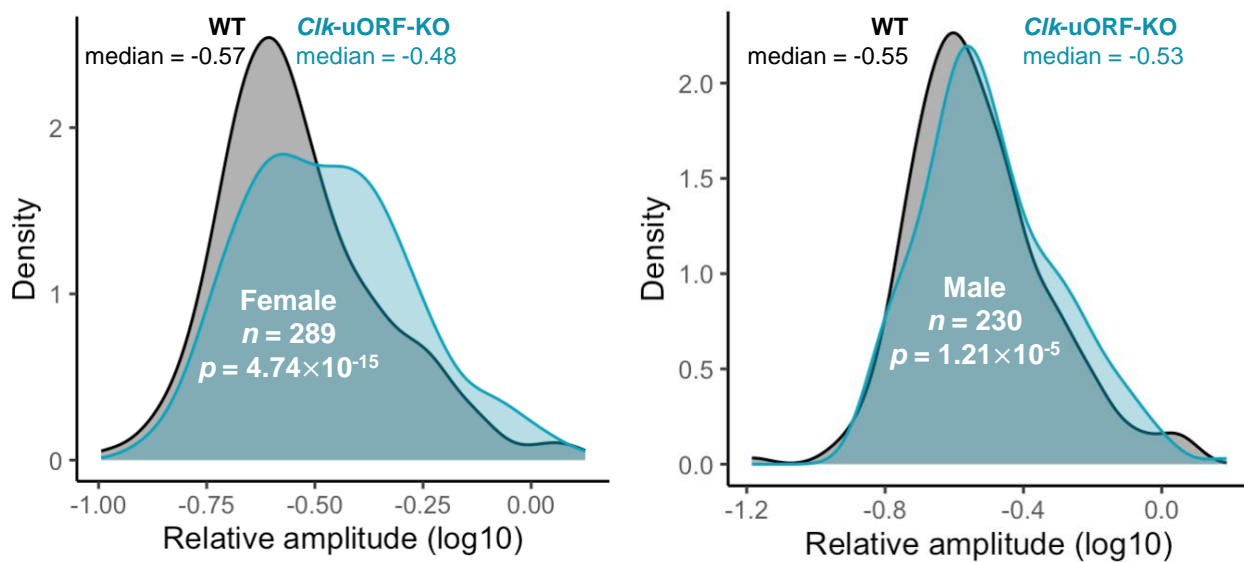

Supplement: S19 Fig — (A) The ratio of circadian rhythm-related genes among cycling genes and non-cycling genes in females and male WT and Clk-uORF-KO. The number of circadian rhythm-related genes and cycling genes or non-cycling genes are denoted at the bottom of each bar. Fisher’s exact tests were applied for comparisons at each ZT. *p < 0.05; **p < 0.01; ***p < 0.001. (B) The distribution of relative amplitudes (rAMP, log10 transformed) for cycling genes in the heads of WT and KO flies (n = 289, p = 4.74 × 10^−15^ for females; n = 230, p = 1.21 × 10^−5^ for males; Wilcoxon signed-rank test). Underlying data for this figure can be found in S1 Data. (PDF) [file pbio.3003173.s019.pdf]

### Females

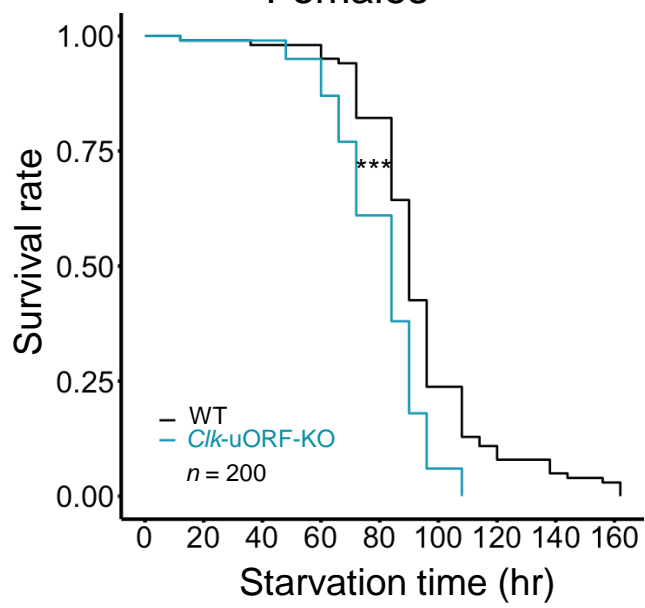

### Males

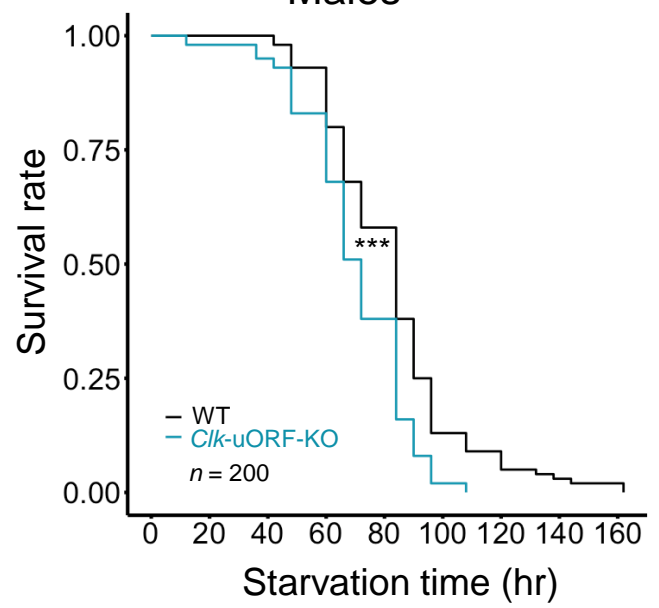

Supplement: S21 Fig — Survival curves of female and male flies under starvation condition (n = 200; log-rank test; ***p < 0.001). Underlying data for this figure can be found in S1 Data. (PDF) [file pbio.3003173.s021.pdf]

**A****Mated females**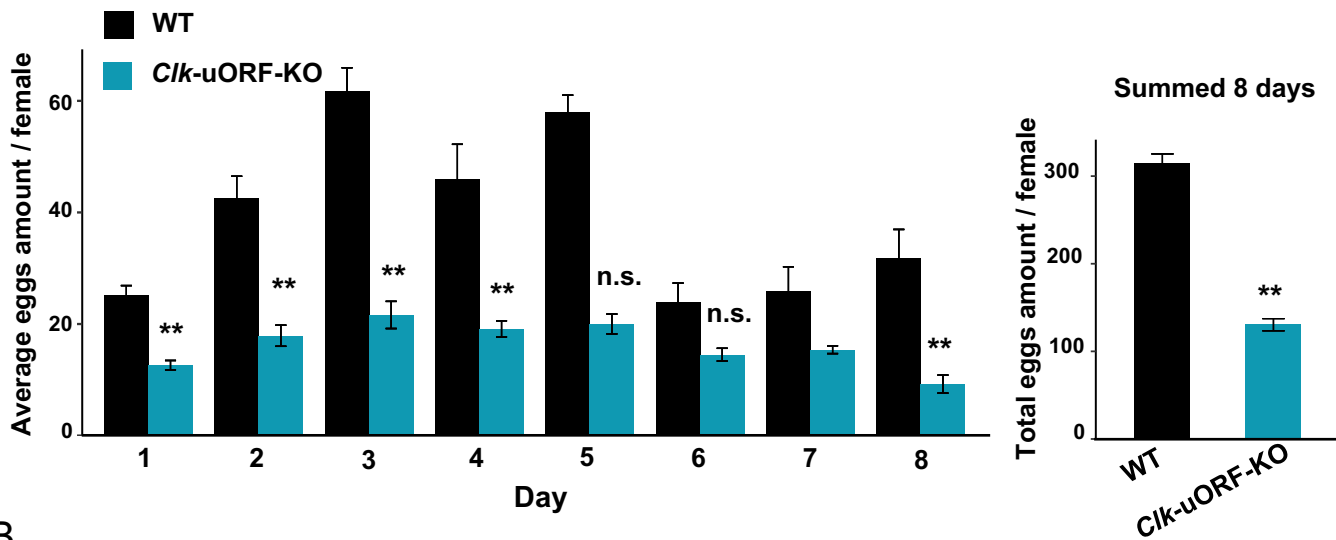**B****Virgin females**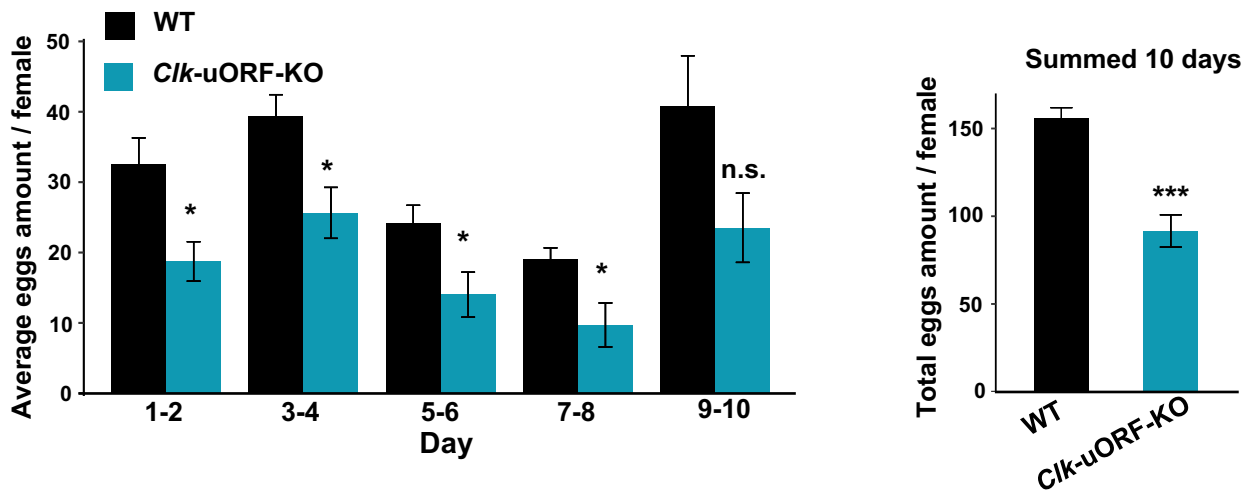**C**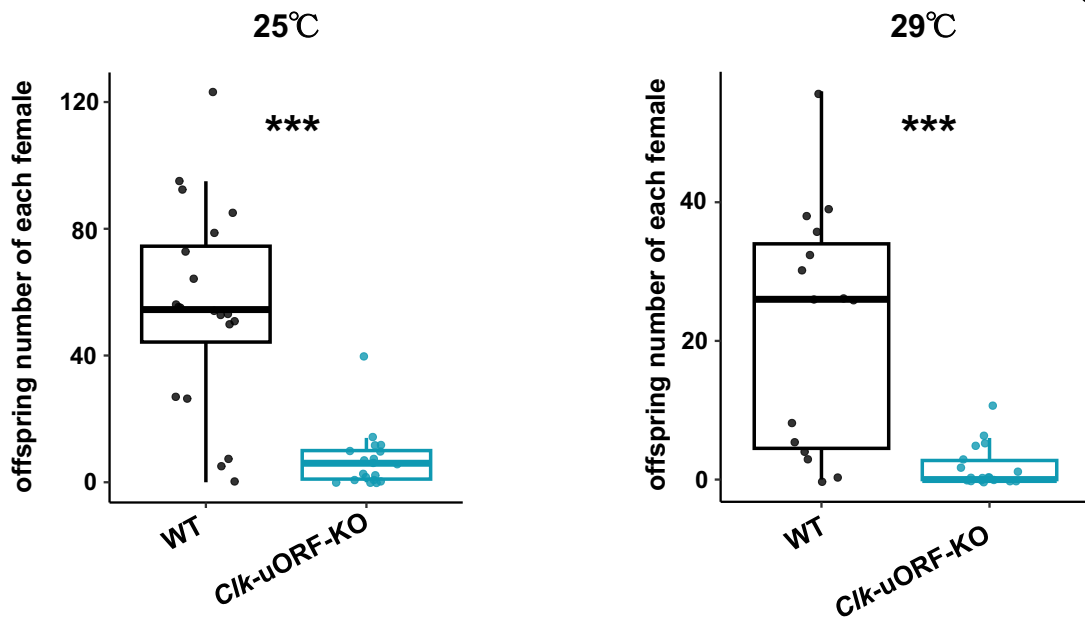

Supplement: S22 Fig — (A) The average 1-day egg number (left) and summed 8-day number (right) laid by a mated female of Clk-uORF-KO and WT over 8 consecutive days. Data are presented as the mean ± SEM (n = 50; Wilcoxon rank-sum test; **p < 0.01; n.s., p > 0.05). (B) The average 2-day egg number (left) and summed 10-day number (right) laid by a virgin of Clk-uORF-KO and WT over 10 consecutive days. Data are presented as the mean ± SEM (n = 50; Wilcoxon rank-sum test; *p < 0.05; ***p < 0.001; n.s., p > 0.05). (C) The offspring number per female parent of Clk-uORF-KO and WT over 10 days at 25°C (left) and 29°C (right) (n = 20; Wilcoxon rank-sum test; ***p < 0.001). Underlying data for this figure can be found in S1 Data. (PDF) [file pbio.3003173.s022.pdf]

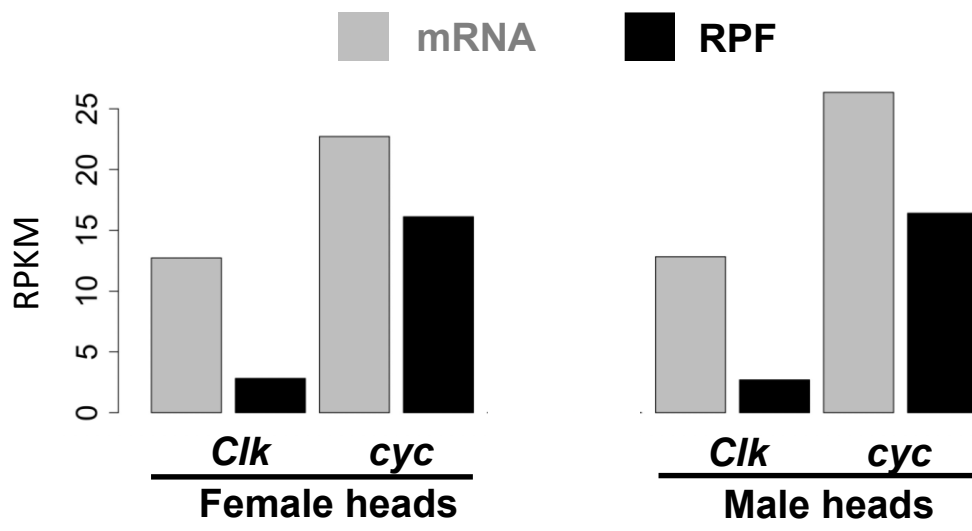

Supplement: S23 Fig — In female heads, Clk exhibits RPKM values of 12.73 in mRNA-Seq and 2.82 in Ribo-Seq, while cyc has values of 22.72 and 16.12, respectively. In male heads, Clk’s RPKM values are 12.83 in mRNA-Seq and 2.70 in Ribo-Seq, compared to cyc’s 26.35 and 6.41, respectively. Analysis was based on our previously published transcriptome and translatome data [39]. (PDF) [file pbio.3003173.s023.pdf]

A

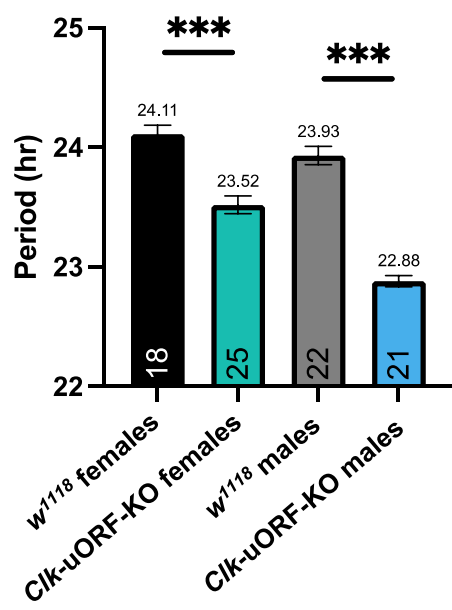

B

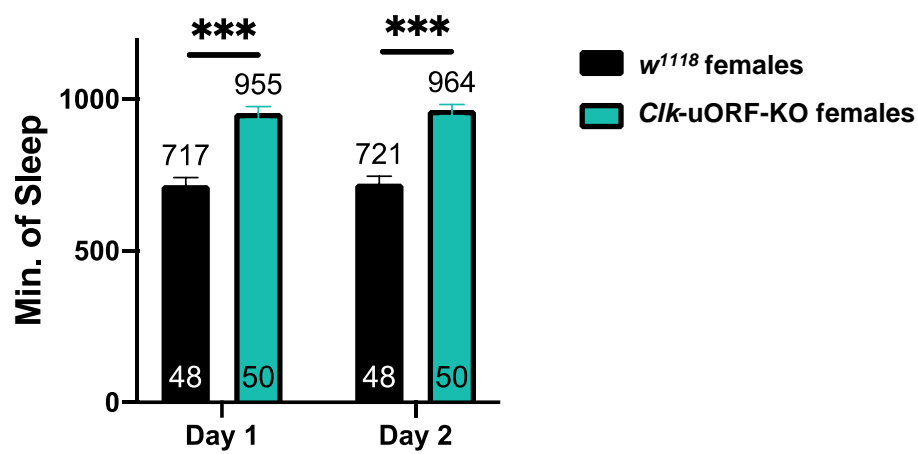

C

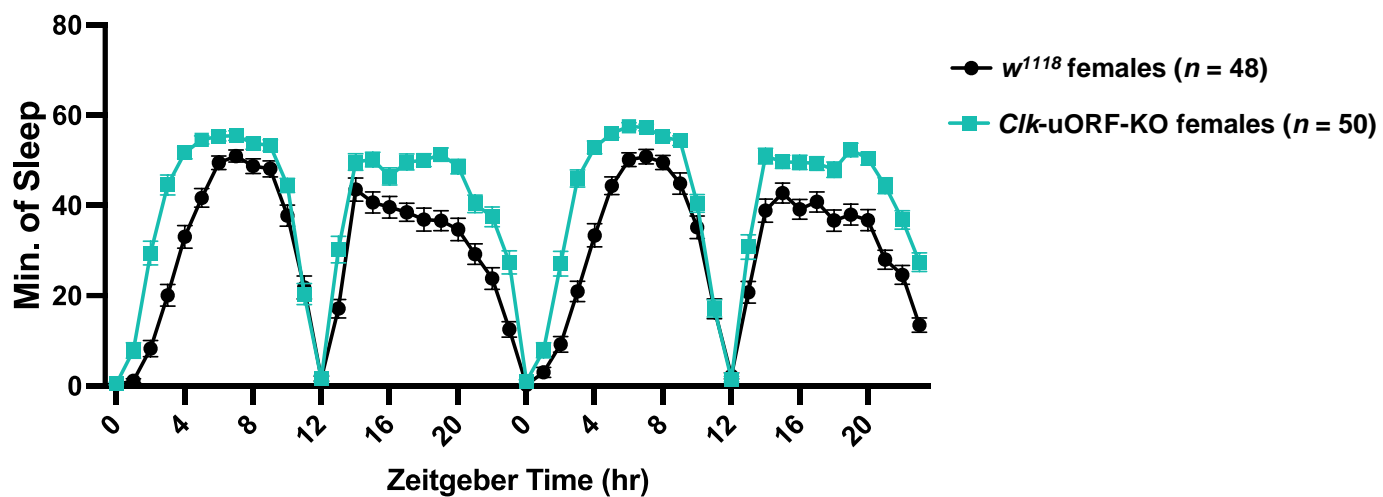

Supplement: S24 Fig — (A) Period length of locomotor rhythms of Clk-uORF-KO flies and w1118 flies under DD. The period lengths (hr) and the number of flies tested (n) are displayed above and below each bar, respectively. (B) Sleep duration over two consecutive days of female Clk-uORF-KO and w1118 flies under LD. The sleep duration (min) of each day and the number of flies tested (n) are displayed above and below each bar, respectively. (C) Sleep profile of female Clk-uORF-KO and w1118 flies under LD conditions. Data are presented as the mean ± SEM. Asterisks indicate statistical significance (Two-tailed Student t test. ***p < 0.001). Underlying data for this figure can be found in S1 Data. (PDF) [file pbio.3003173.s024.pdf]

A

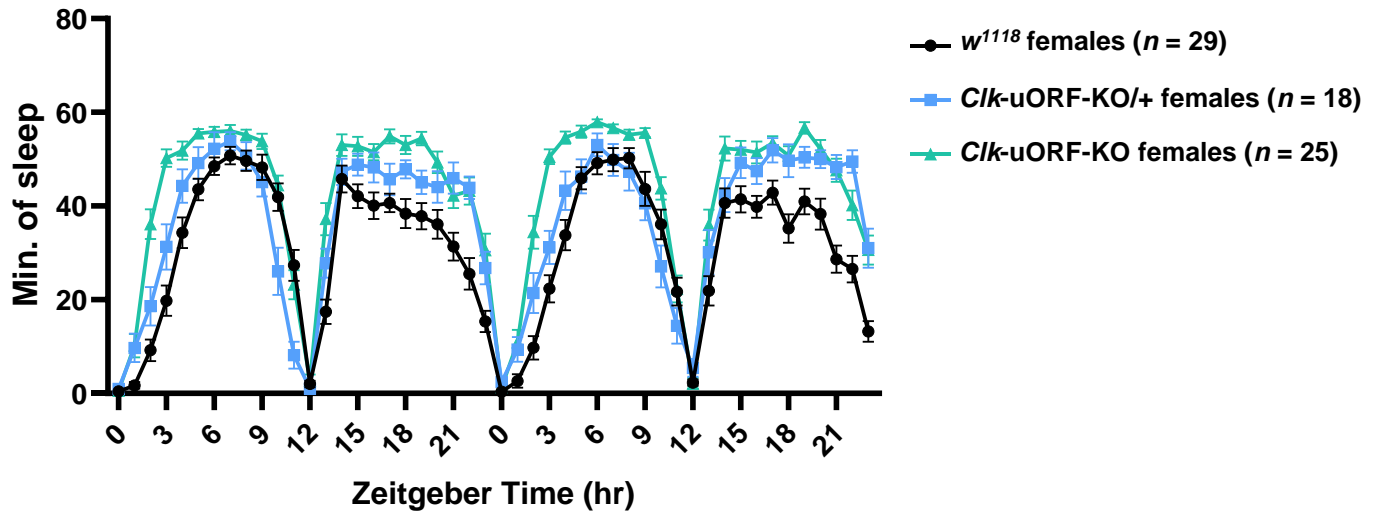

B

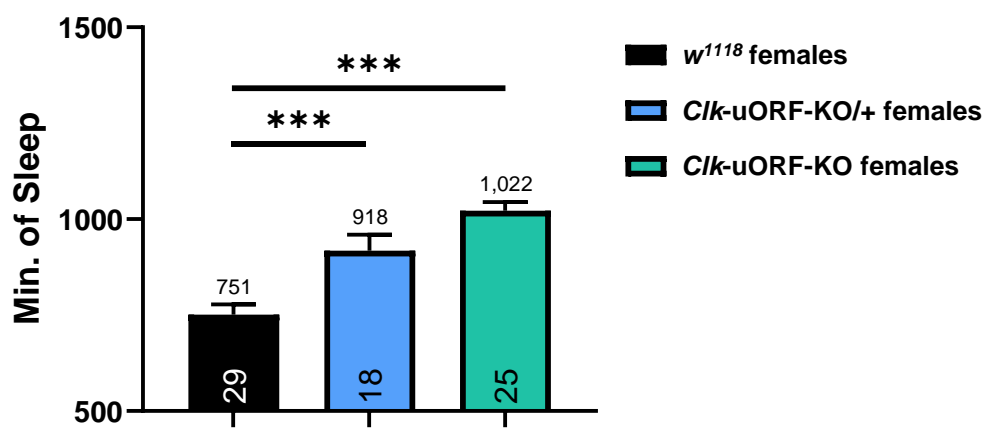

Supplement: S25 Fig — (A) Sleep profile of female w1118, heterozygous and homozygous Clk-uORF-KO under LD conditions. (B) Sleep duration over two consecutive days of indicated flies in (A). The sleep duration (min) of each day and the number of flies tested (n) are displayed above and below each bar, respectively. Data are presented as the mean ± SEM. Asterisks indicate statistical significance (Two-tailed Student t test. ***p < 0.001). Underlying data for this figure can be found in S1 Data. (PDF) [file pbio.3003173.s025.pdf]

A

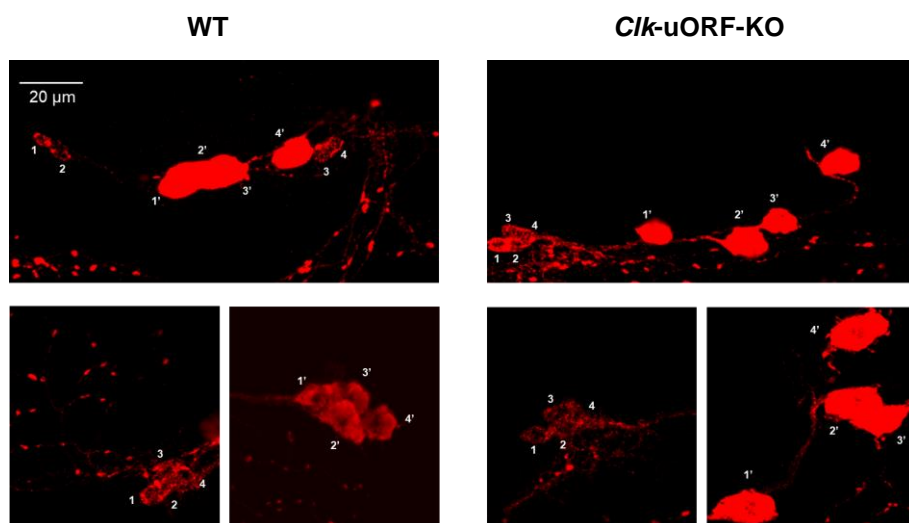

B

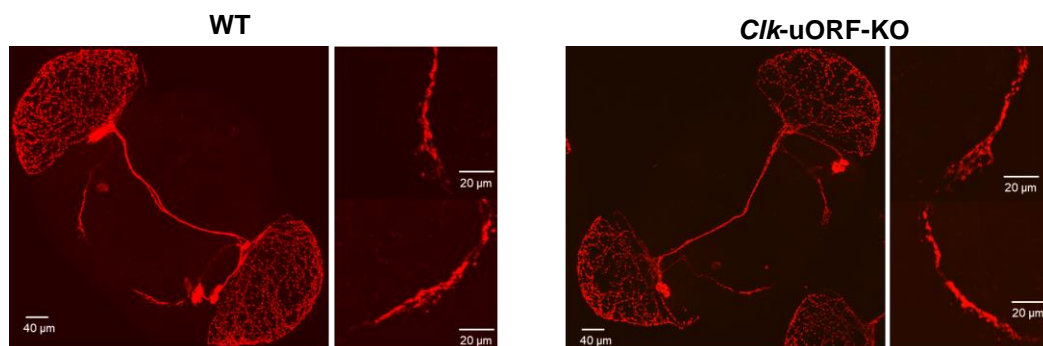

C

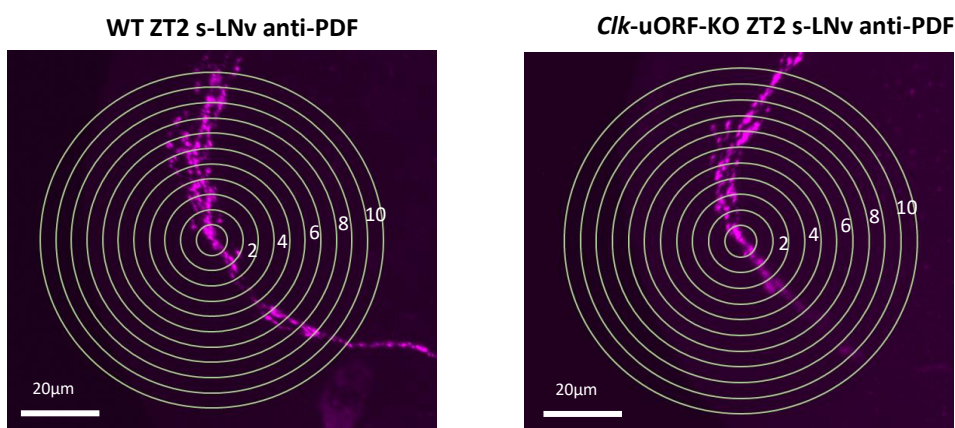

D

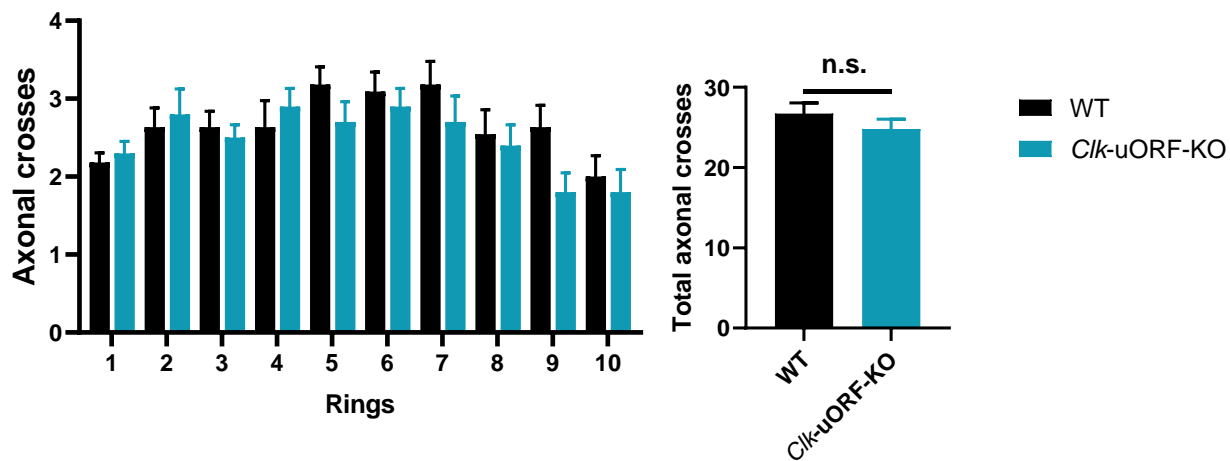

Supplement: S26 Fig — (A and B) Representative example of s-LNvs (1, 2, 3 and 4) and l-LNvs (1′, 2′, 3′ and 4′) soma (A) and the morphology of PDF projections (B) in female WT and Clk-uORF-KO. The scale bars are represented respectively. (C) Sholl analysis of the complexity of the s-LNvs axonal arbor stained by PDF antibody. Concentric circles separated by 5 μm were centered at the point of intersection, where the projections of the s-LNvs intersect. (D) Quantification of the axonal cross with concentric circles in (C). Data are reported as the mean ± SEM (n = 6; Two-tailed Mann–Whitney U test for unpaired comparisons. n.s., p > 0.05). Underlying data for this figure can be found in S1 Data. (PDF) [file pbio.3003173.s026.pdf]

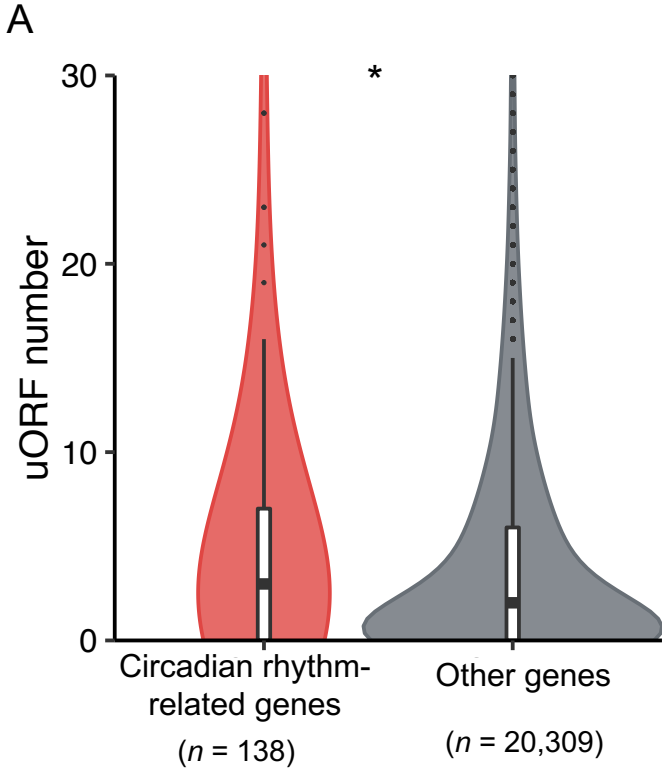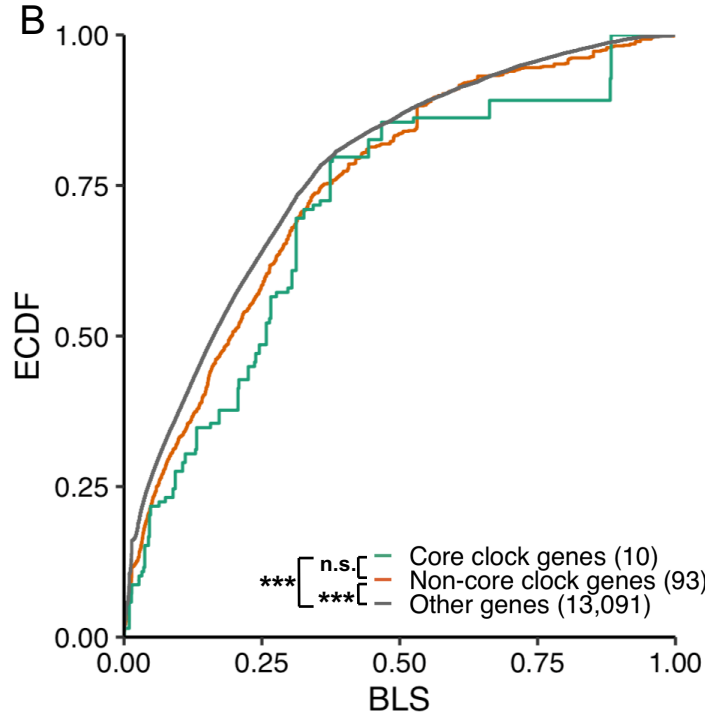

Supplement: S27 Fig — (A) The distribution of uORF number in circadian rhythm-related genes and other genes in humans. The gene number (n) in each class are denoted at the bottom (Wilcoxon rank-sum test, p = 0.033). (B) ECDF of the BLSs for uATGs in core clock genes, non-core clock genes and other genes, respectively. The gene number in each class is denoted in the parentheses (Wilcoxon rank-sum test, p = 1.92 × 10^−5^ for non-core clock genes versus other genes; p = 0.08 for core clock gene versus non-core clock genes; p = 0.00046 for core clock gene versus other genes. n.s., p > 0.05; *p < 0.05; ***p < 0.001). Underlying data for this figure can be found in S1 Data. (PDF) [file pbio.3003173.s027.pdf]
