# Supplementary material for: Upstream open reading frames dynamically modulate CLOCK protein translation to regulate circadian rhythms and sleep
Source: PLoS Biol. 2025 May 12;23(5):e3003173. doi: 10.1371/journal.pbio.3003173 (PMC12121920; doi:10.1371/journal.pbio.3003173)
Supplement: S5 Fig — (A) Diagram illustrating the separation of monosomes and polysomes in a sucrose density gradient (10−45%). The P-to-M ratio is the ratio of mRNA abundance in the polysome fraction to that in the monosome fraction. (B) P-to-M ratios of cyc mRNA from whole heads of male Clk-uORF-KO compared to that in male WT flies, sampled at indicated Zeitgeber times at 4-hr intervals. Data are expressed as mean ± SEM (n = 6; Wilcoxon signed-rank test; *p < 0.05; **p < 0.01; ***p < 0.001; n.s., p > 0.05). Underlying data for this figure can be found in S1 Data. (PDF) [file pbio.3003173.s005.pdf]

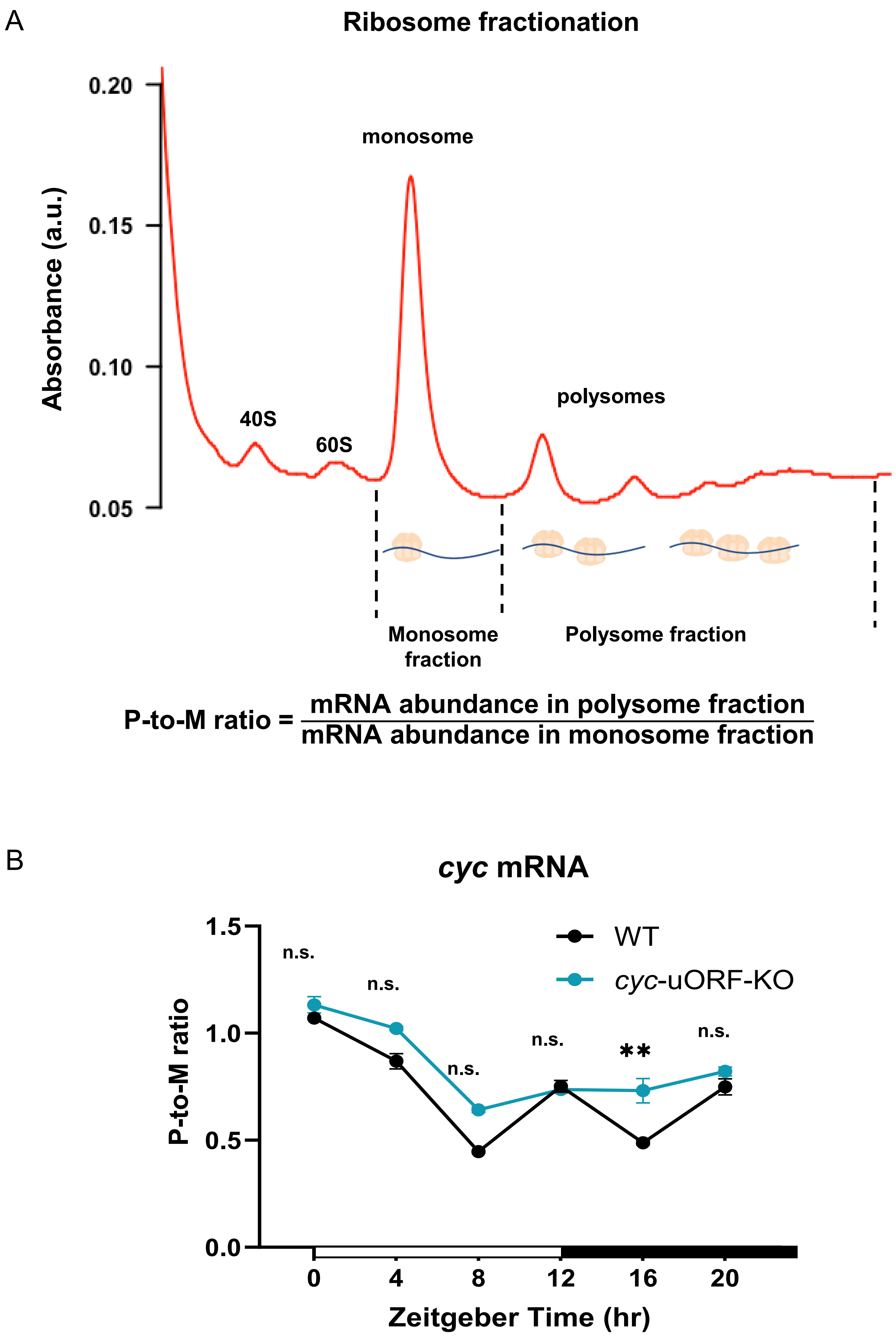
A
Ribosome fractionation
Absorbance (a.u.)
0.20
0.15
0.10
0.05
monosome
40S
60S
polysomes
Monosome fraction
Polysome fraction
P-to-M ratio = mRNA abundance in polysome fraction / mRNA abundance in monosome fraction
B
cyc mRNA
P-to-M ratio
1.5
1.0
0.5
0.0
WT
cyc-uORF-KO
n.s.
n.s.
n.s.
n.s.
**
n.s.
0
4
8
12
16
20
Zeitgeber Time (hr)
