# Supplementary material for: Upstream open reading frames dynamically modulate CLOCK protein translation to regulate circadian rhythms and sleep
Source: PLoS Biol. 2025 May 12;23(5):e3003173. doi: 10.1371/journal.pbio.3003173 (PMC12121920; doi:10.1371/journal.pbio.3003173)
Supplement: S20 Fig — (A) The overlap of cycling genes in WT female and WT male. (B) The overlap of cycling genes in Clk-uORF-KO female and Clk-uORF-KO male. (C) The overlap of genes that gain rhythmic expression in female and male Clk-uORF-KO. (D) The overlap of genes that loss rhythmic expression in female and male Clk-uORF-KO. (E) Heatmap showing the GO terms enriched among genes that gained/lost rhythmic expression in females and males. The heatmap cells were colored by their p-values, while gray cells indicate lack of enrichment for that term in the corresponding gene sets. (PDF) [file pbio.3003173.s020.pdf]

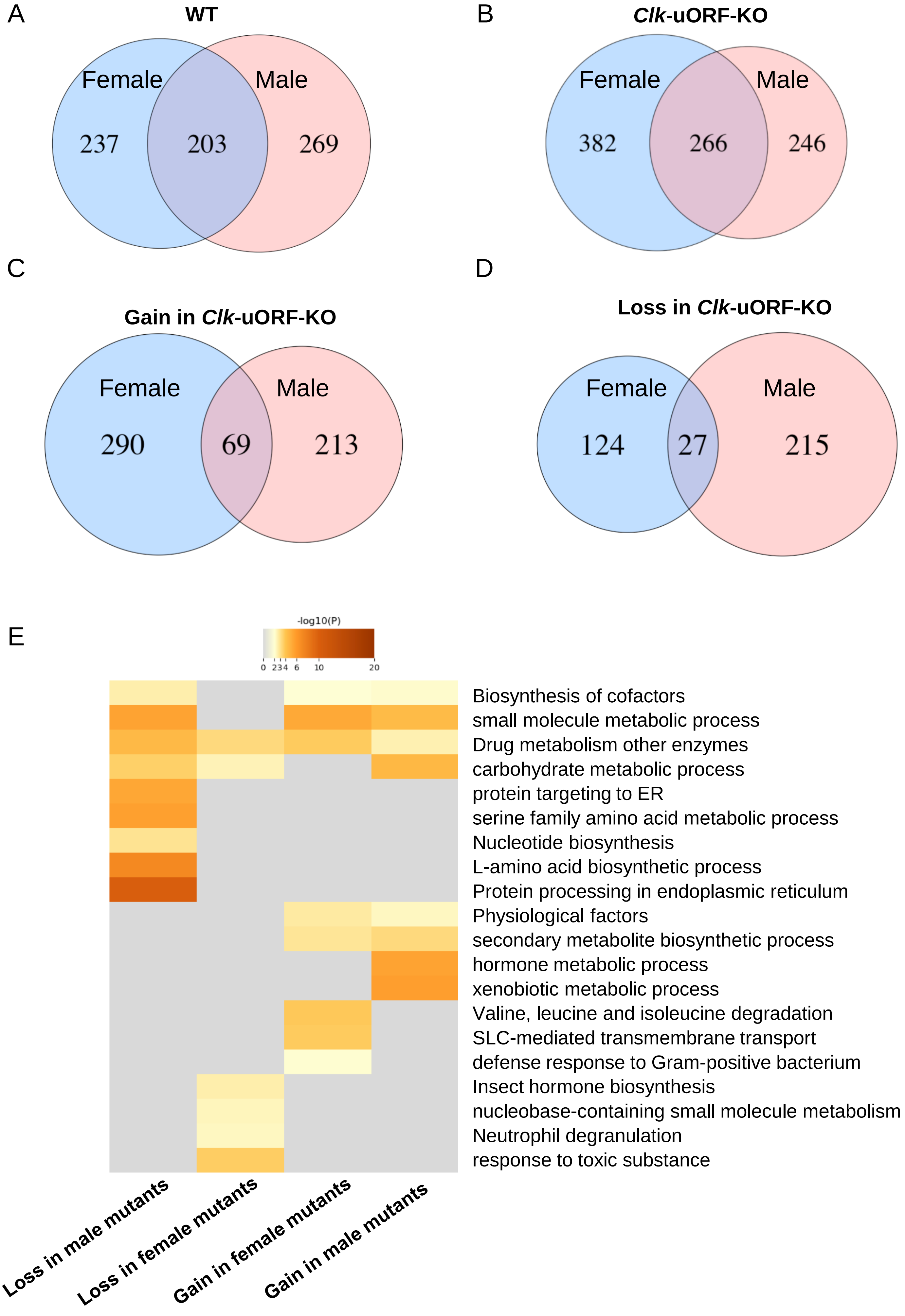

A
WT
Female
Male
237
203
269
B
Clk-uORF-KO
Female
Male
382
266
246
C
Gain in Clk-uORF-KO
Female
Male
290
69
213
D
Loss in Clk-uORF-KO
Female
Male
124
27
215
E
-log10(P)
0 2 3 4 6 10 20
Biosynthesis of cofactors
small molecule metabolic process
Drug metabolism other enzymes
carbohydrate metabolic process
protein targeting to ER
serine family amino acid metabolic process
Nucleotide biosynthesis
L-amino acid biosynthetic process
Protein processing in endoplasmic reticulum
Physiological factors
secondary metabolite biosynthetic process
hormone metabolic process
xenobiotic metabolic process
Valine, leucine and isoleucine degradation
SLC-mediated transmembrane transport
defense response to Gram-positive bacterium
Insect hormone biosynthesis
nucleobase-containing small molecule metabolism
Neutrophil degranulation
response to toxic substance
Loss in male mutants
Loss in female mutants
Gain in female mutants
Gain in male mutants
