# Supplementary material for: Upstream open reading frames dynamically modulate CLOCK protein translation to regulate circadian rhythms and sleep
Source: PLoS Biol. 2025 May 12;23(5):e3003173. doi: 10.1371/journal.pbio.3003173 (PMC12121920; doi:10.1371/journal.pbio.3003173)
Supplement: S1 Text — (DOCX) [file pbio.3003173.s028.docx]

**Ribosome fraction analysis**

The heads obtained from WT and mutant flies were collected and homogenized in a Dounce homogenizer with lysis buffer [50 mM Tris pH 7.5, 150 mM NaCl, 5 mM MgCl_2_, 1% Triton X-100, 2 mM dithiothreitol (DTT), 20 U/ml SuperaseIn (Ambion), 0.5 tablets of proteinase inhibitor (Roche), 100 μg/ml emetine (Sigma Aldrich), and 50 μM guanosine 5′-[β,γ-imido]triphosphate trisodium salt hydrate (GMP-PNP) (Sigma Aldrich)] at 4°C. The lysates were clarified by centrifugation at 4°C and 20,000×g for 10 min, and the supernatants were transferred to new 1.5 ml tubes. 10-45% sucrose gradients were prepared in buffer (250 mM NaCl, 50 mM Tris pH 7.5, 15 mM MgCl_2_, 0.5 mM DTT, 12 U/ml RNaseOUT, 0.5 tablets of protease inhibitor, and 20 μg/ml emetine) using a Gradient Master (Biocomp Instruments) in ULTRA-CLEAR Thinwall Tubes (Beckman Coulter). A sample volume of up to 500 μl was applied to the top of each gradient. After ultracentrifugation with a Hitachi P40ST rotor at 35,000 × rpm for 3 hr at 4°C, the monosome and polysome fractions were collected, flash-frozen in liquid nitrogen, and stored at -80°C until further use.

**Model simulation for the outcome of enhanced *Clk* translation**

Briefly, assuming that genes or proteins *j* $\in$ *{1,, n}* regulate the generation of gene/protein *i*, the subsequent type of differential equations as a general model were employed:

$$\frac{{dx}_{i}(t)}{dx} = \rho_{i}g\left( \sum_{j=1}^{n} \lambda_{ji}x_{j}\left( t \right) - \delta_{i}x_{i}\left( t \right) \right)x_{i}\left( t \right)\left( s_{i}-x_{i}\left( t \right) \right), 1\leq j \leq n, (1)$$

where the state vector $x_{i}$ represents the concentration of molecule *i* at its site of action. The parameters $\lambda_{ji}$ represent regulatory weights that indicate the influence of molecule *j* on the production rate of molecule *i*. Positive and negative values of $\lambda_{ji}$ indicate activating or repressing effects, respectively. The larger absolute value of $\lambda_{ji}$, the stronger effect. It is worth noting that $\lambda_{Clk \to\mathrm{CLK}}$ in the model is applicable to the regulation of *Clk* mRNA to CLK protein (i.e., translation efficiency) in our study. To mimic the enhanced CLK translation caused by uORFs removal, we increased the original $\lambda_{Clk \to\mathrm{CLK}}$ from 0.026 (baseline) to three larger arbitrary values: 0.027, 0.028 and 0.030, respectively.

An odd sigmoid function modulates the cumulative regulatory influences, g: $\mathbb{R}$→$\mathbb{R}$, of the form

$$g\left( \mu\right)=\frac{\mu}{\sqrt{1+\mu^{2}}}=\tanh\left( ln(\mu+\sqrt{1+\mu^{2}}) \right),$$

together with a parameter $\rho_{i}$ > 0 that indicates the maximum rate of *i* production. The model incorporates logistic terms $x_{i}\left( s_{i}-x_{i} \right)$, where constants $s_{i}$ ≥ 0 indicate the saturation level of molecule *i*. The real parameter $\delta_{i}$ is the decay rate of *i*.

Simulations were performed on MATLAB (The MathWorks, Natick, MA). The system of ordinary differential equations is solved numerically by the subroutine ‘‘ode45’’. The actual equations and parameters are the same as those provided in the original model [1]. During each simulation cycle, the mRNA and protein levels of each molecule in the circadian network were recorded. We calculated the time intervals between the peaks of *tim* mRNA, serving as the proxy of circadian period length under the different regulatory weights.

Reference

1. Fathallah-Shaykh HM, Bona JL, Kadener S. Mathematical model of the Drosophila circadian clock: loop regulation and transcriptional integration. Biophys J. 2009;97(9):2399-408. Epub 2009/11/04. doi: 10.1016/j.bpj.2009.08.018. PubMed PMID: 19883582; PubMed Central PMCID: PMCPMC2770617.
