# Supplementary material for: Upstream open reading frames dynamically modulate CLOCK protein translation to regulate circadian rhythms and sleep
Source: PLoS Biol. 2025 May 12;23(5):e3003173. doi: 10.1371/journal.pbio.3003173 (PMC12121920; doi:10.1371/journal.pbio.3003173)
Supplement: S1 Raw Images — (PDF) [file pbio.3003173.s038.pdf]

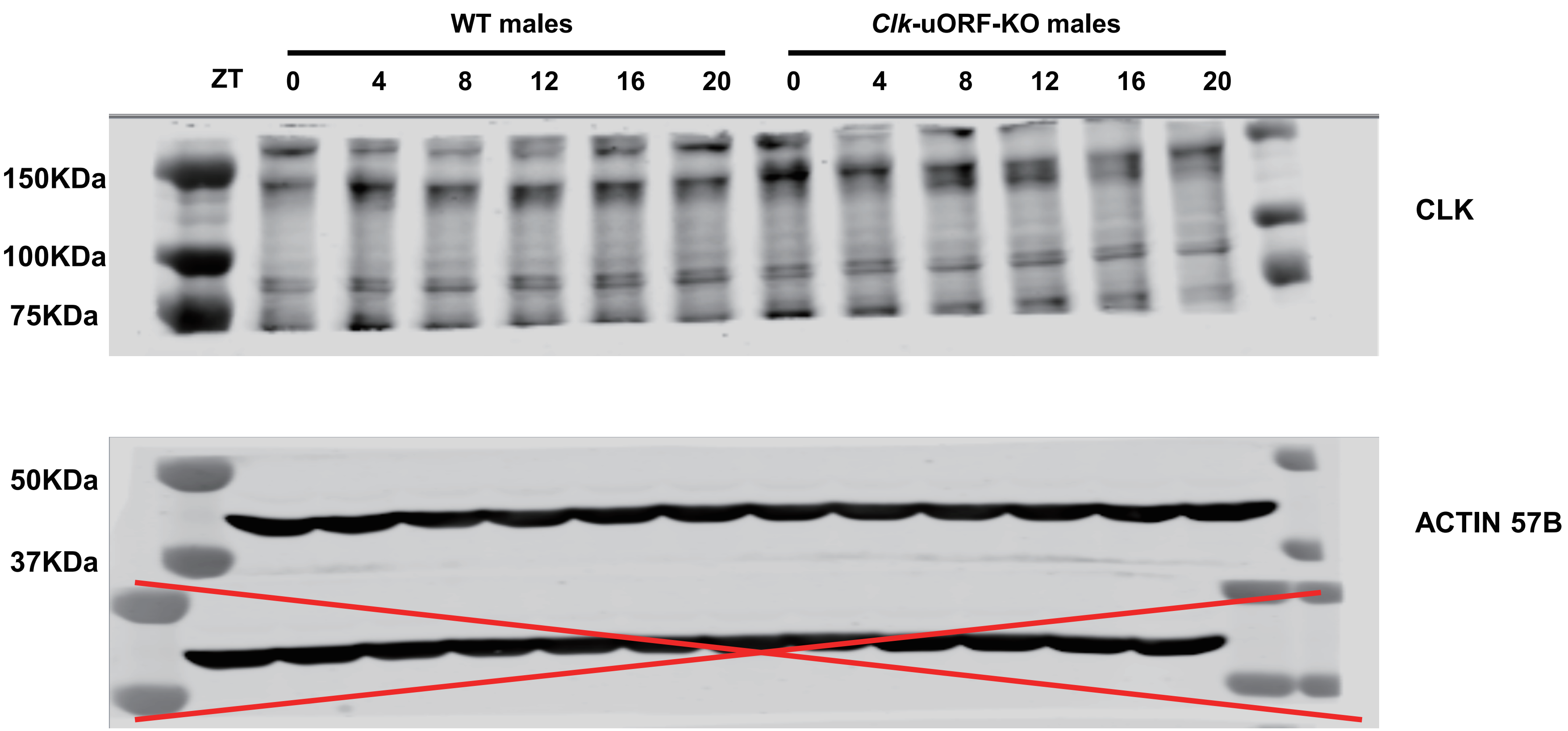

Scans Figure 3C

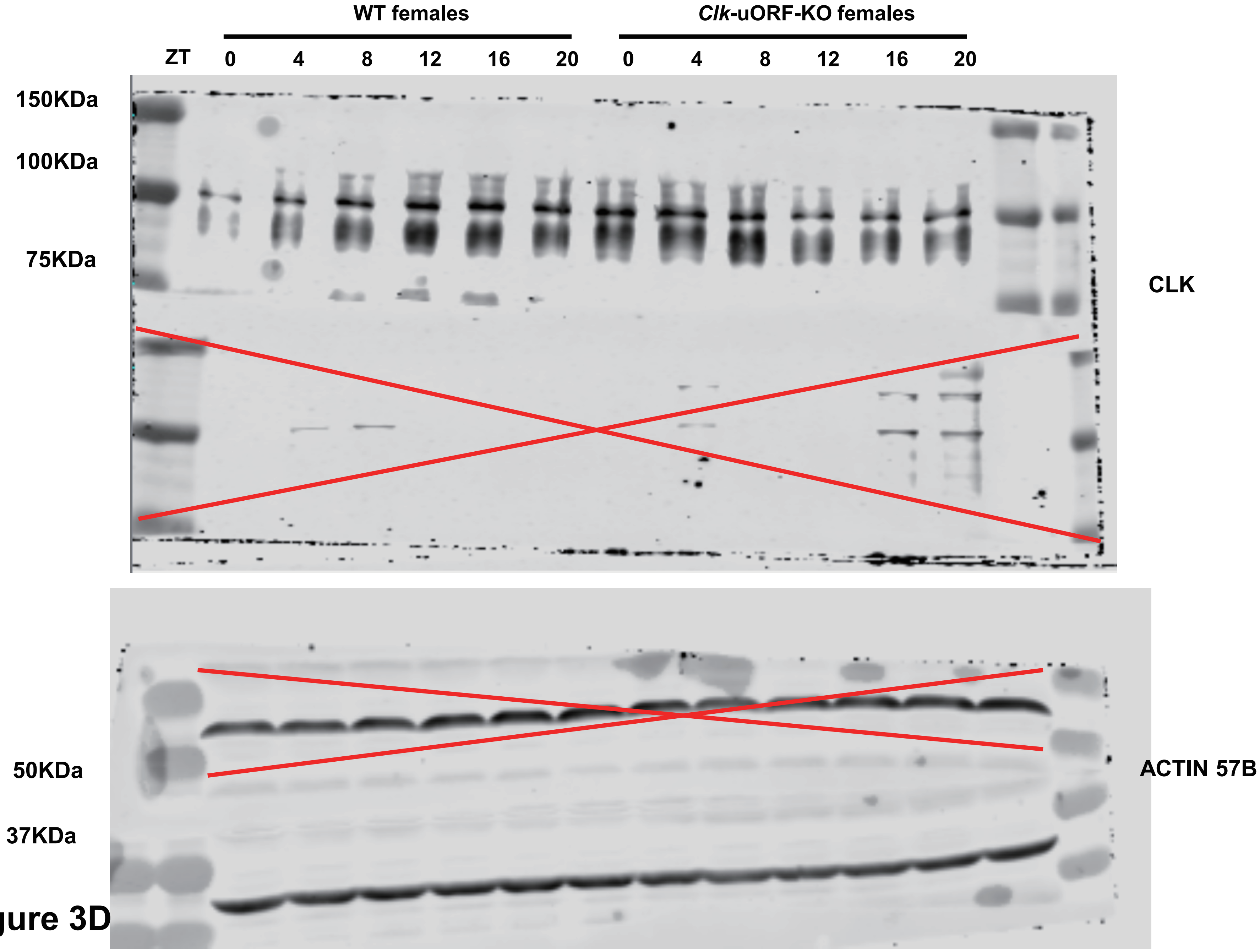

Scans Figure 3D

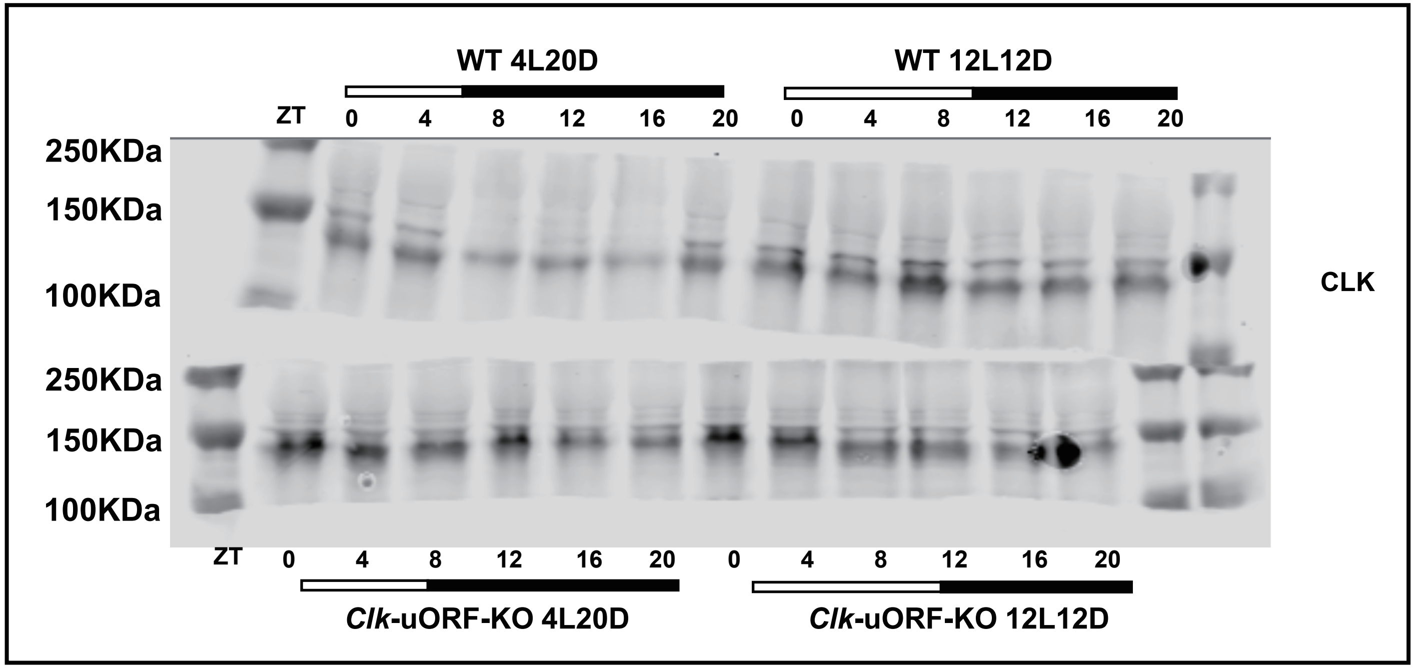

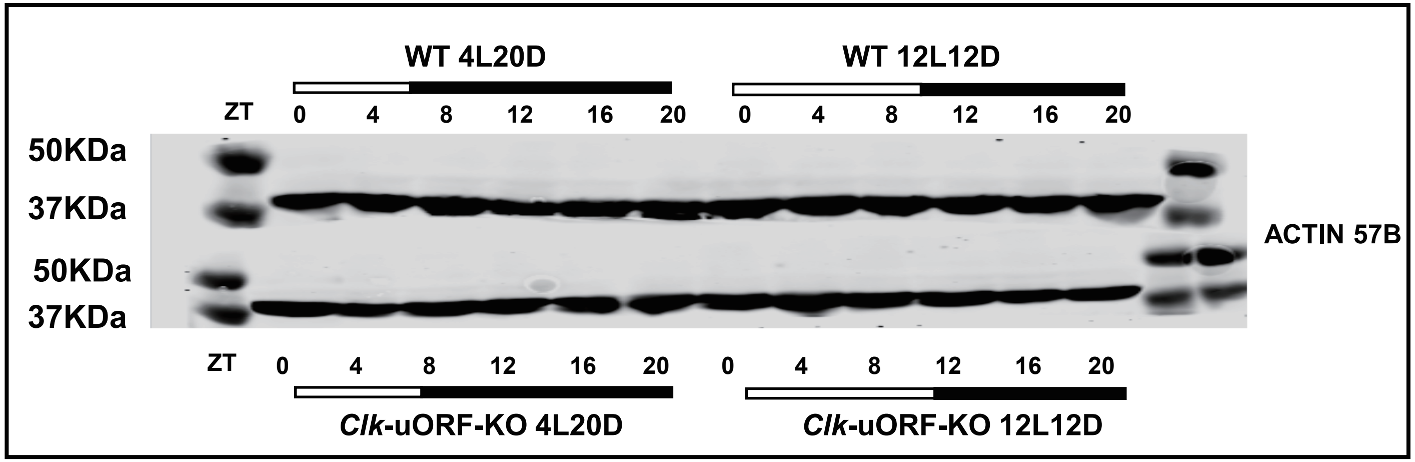

Scans Figure 7E

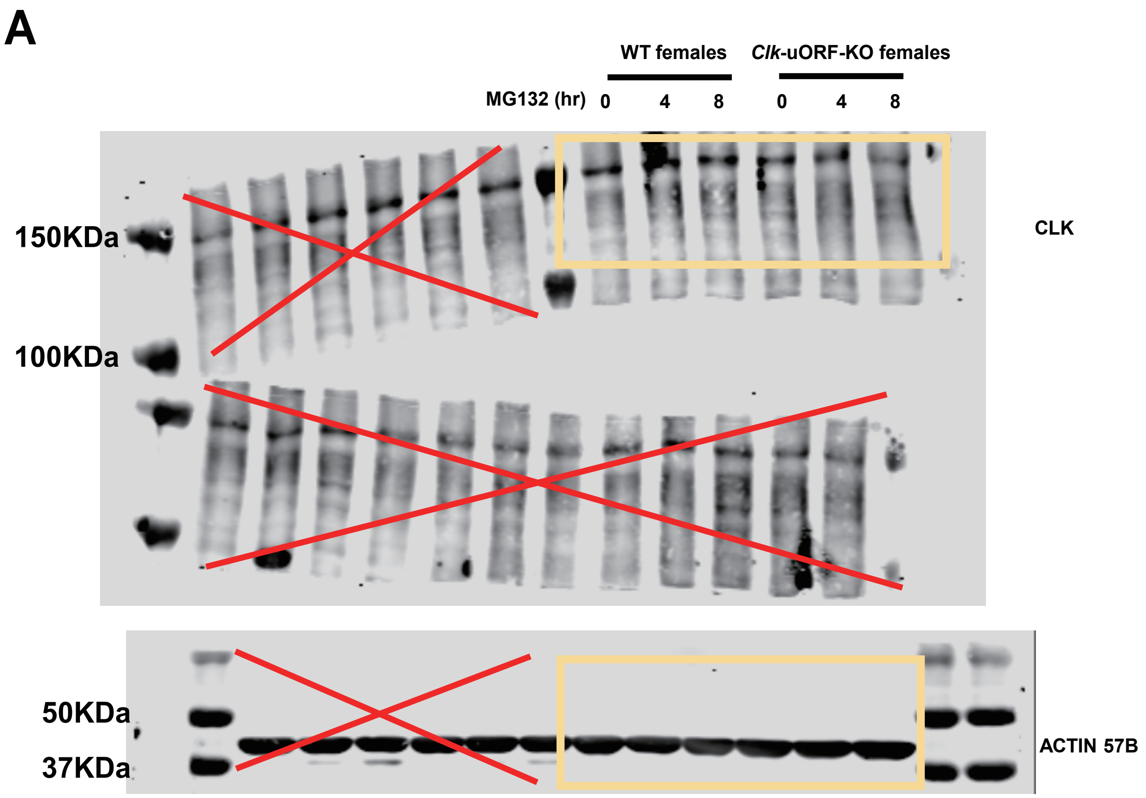

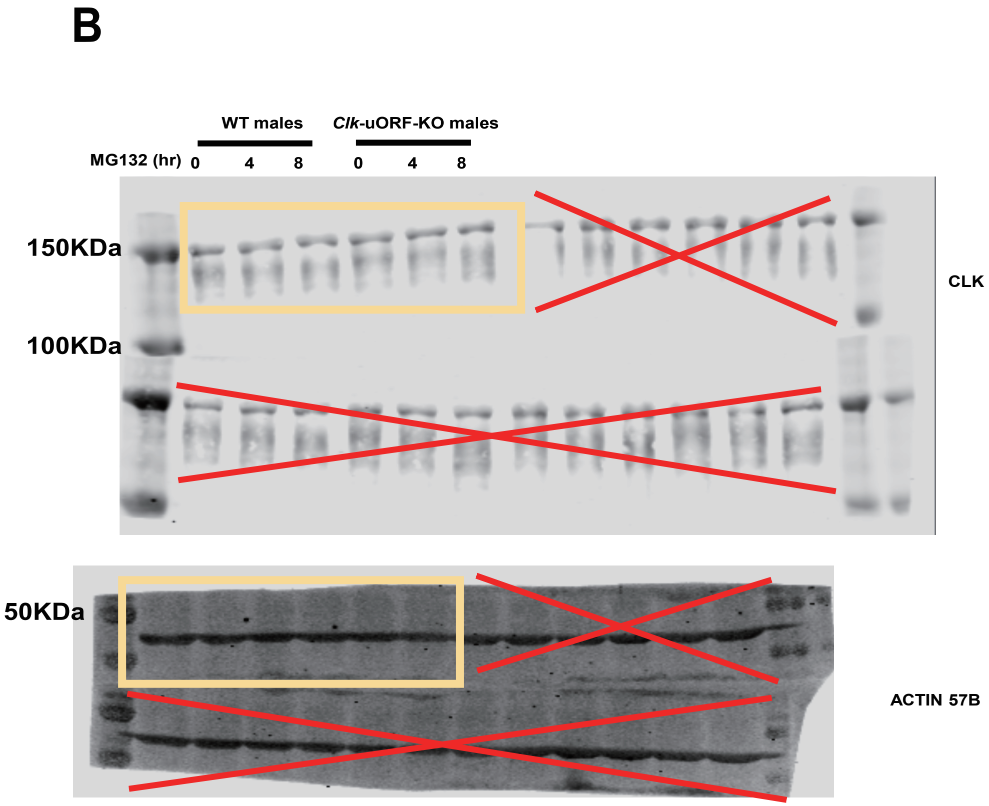

A. Scans Figure S7A

B. Scans Figure S7C

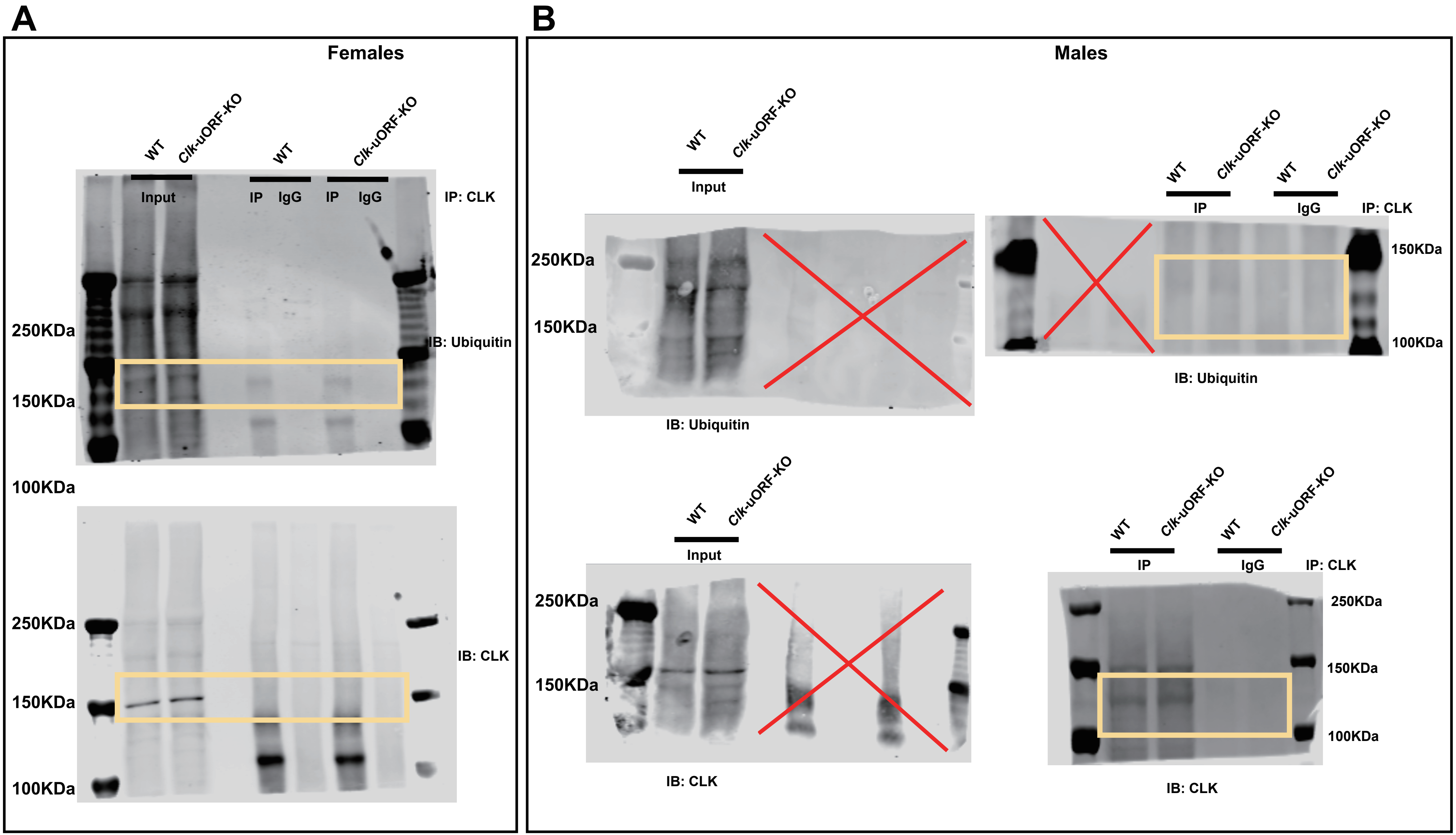

A. Scans Figure S7E

B. Scans Figure S7F

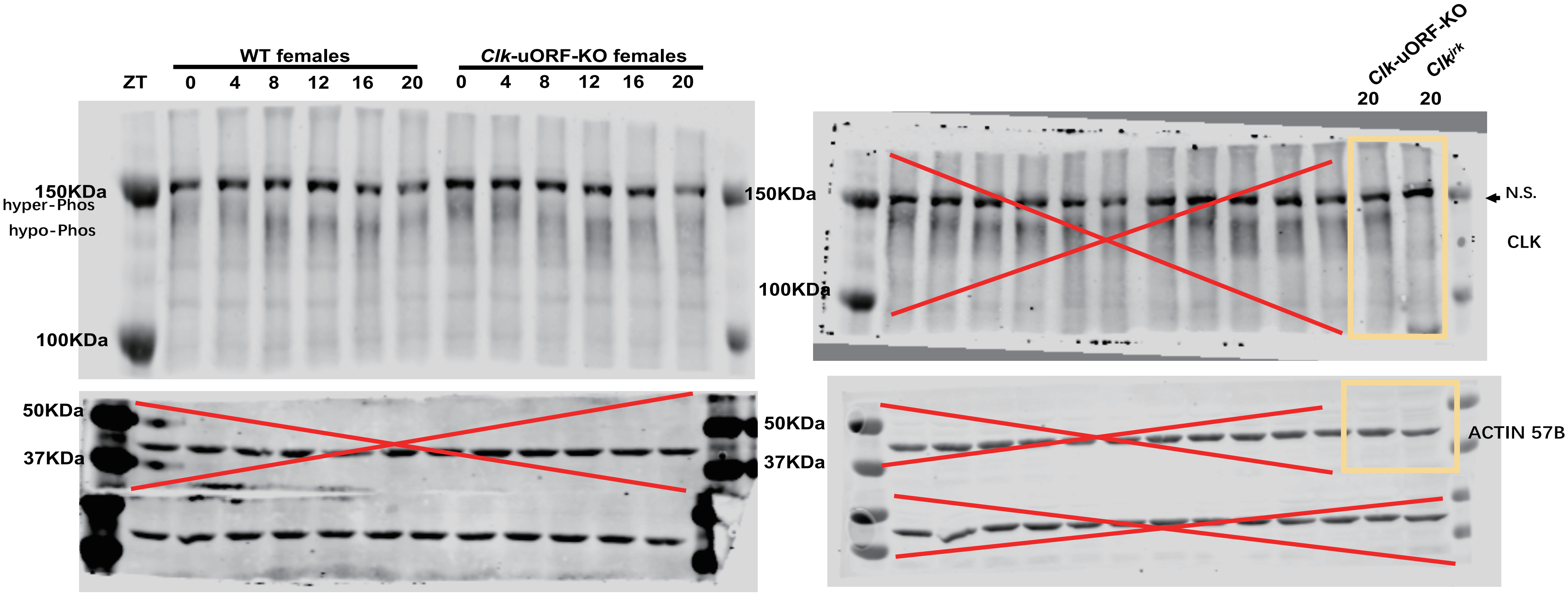

Scans Figure S8A

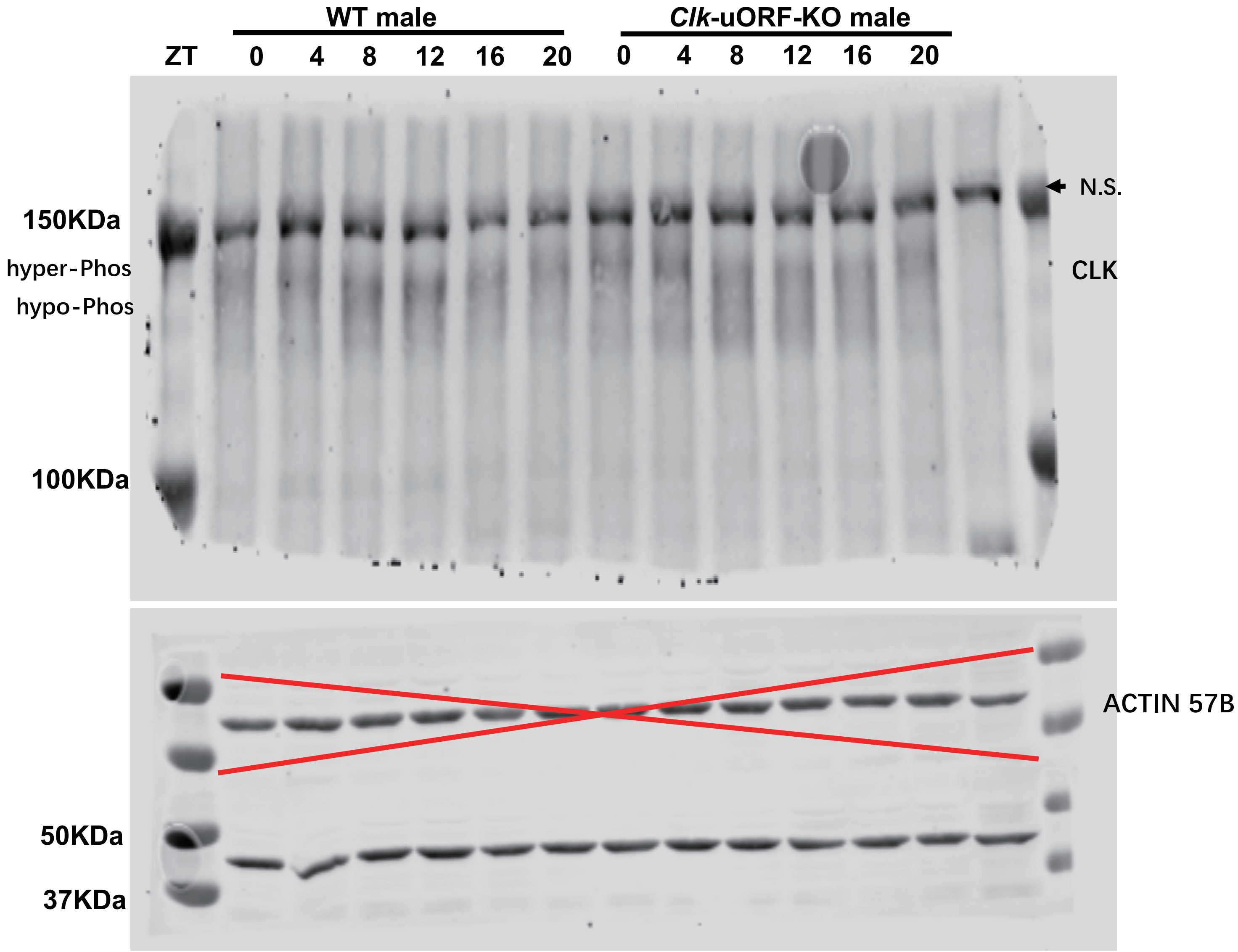

Scans Figure S8C

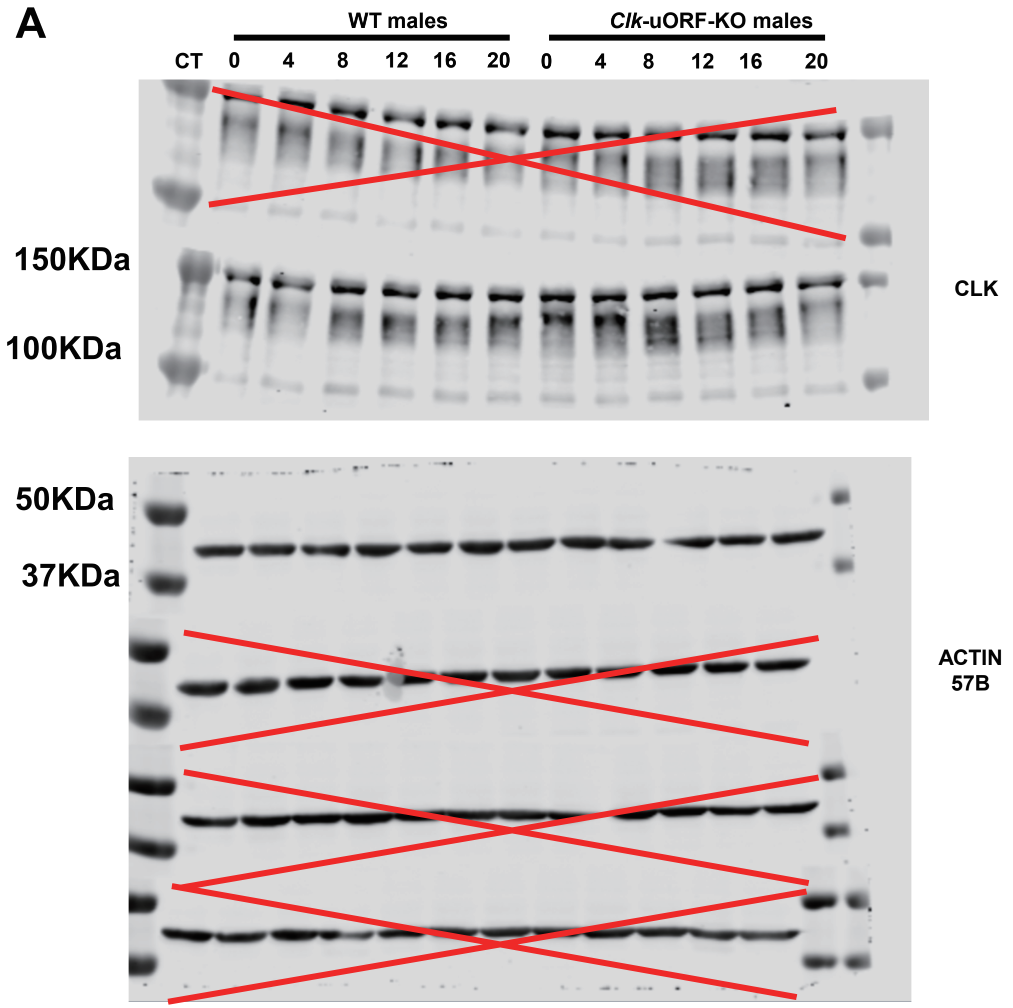

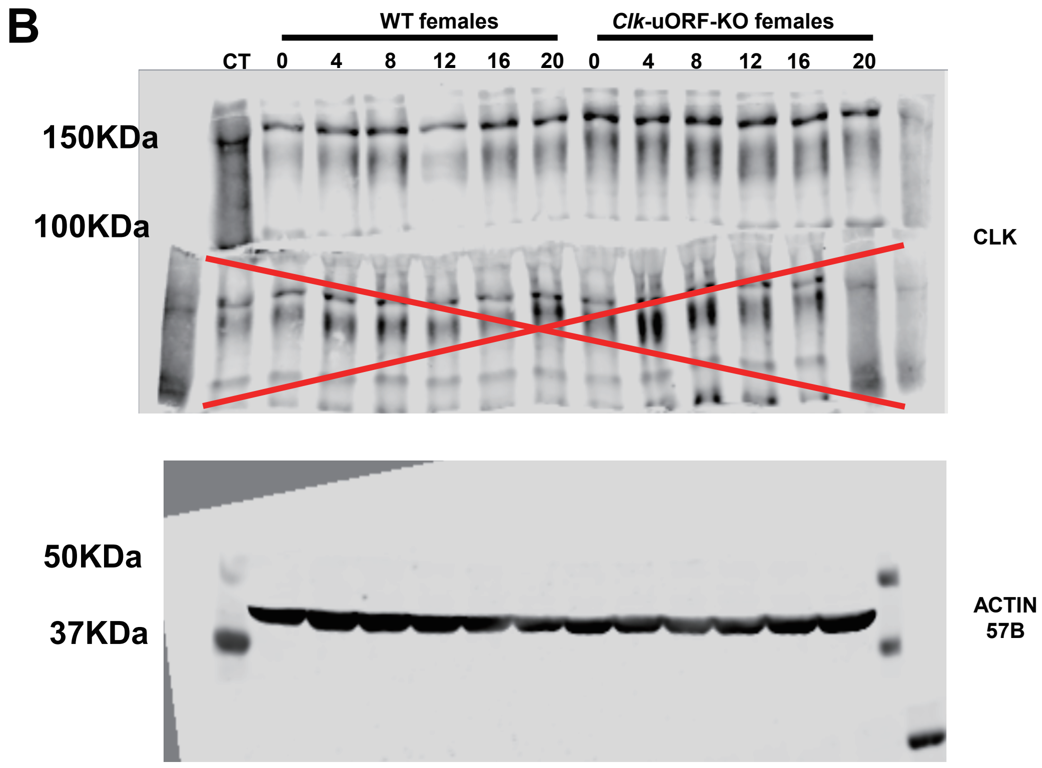

A. Scans Figure S9A

B. Scans Figure S9C

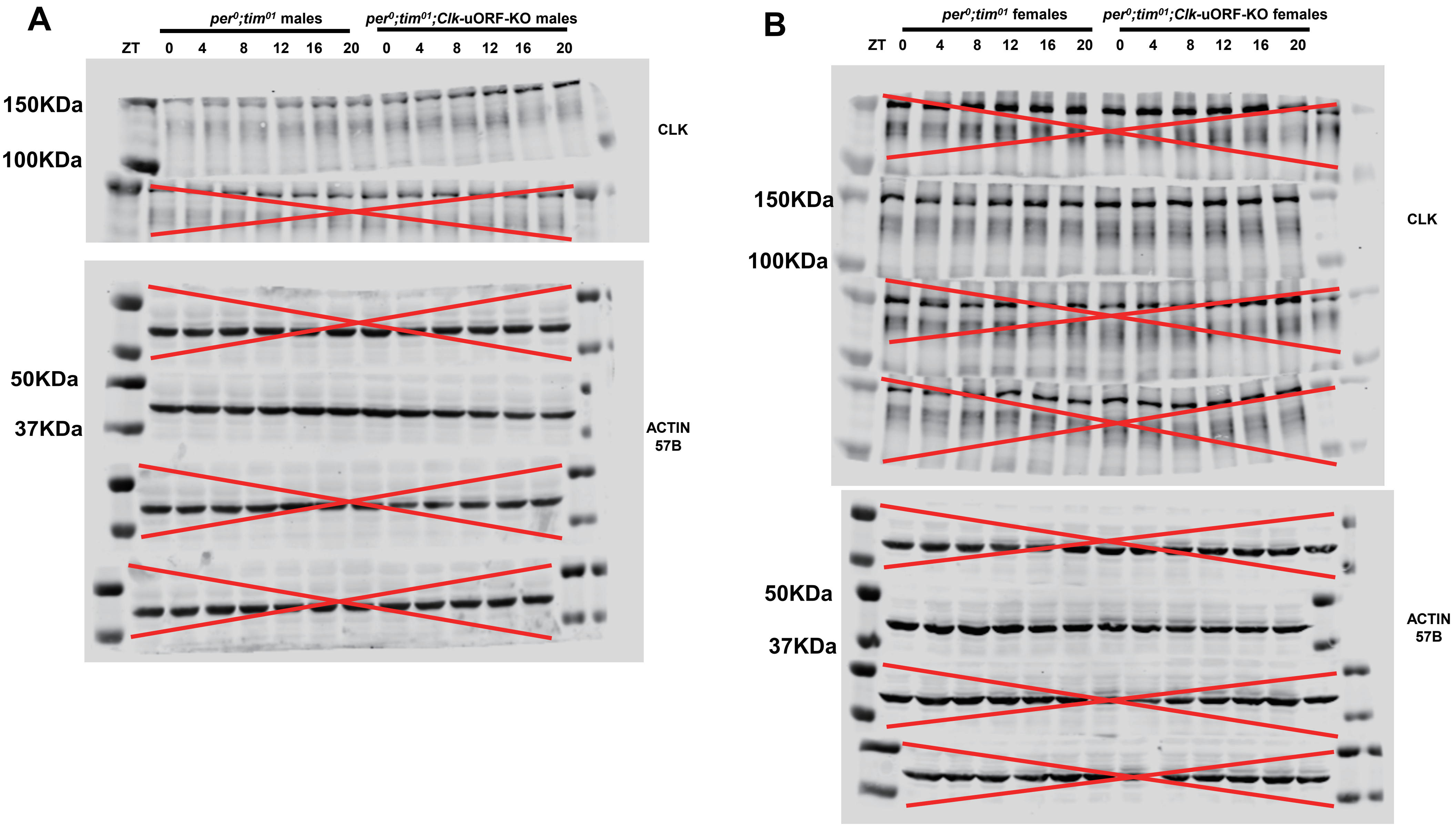

A. Scans Figure S9E

B. Scans Figure S9G
